# Highly specific gene silencing in a monocot species by artificial microRNAs derived from chimeric *miRNA* precursors

**DOI:** 10.1111/tpj.12835

**Published:** 2015-05-20

**Authors:** Alberto Carbonell, Noah Fahlgren, Skyler Mitchell, Kevin L. Cox, Kevin C. Reilly, Todd C. Mockler, James C. Carrington

**Affiliations:** ^1^ Donald Danforth Plant Science CenterSt. LouisMO63132USA; ^2^ Present address: Department of Plant Pathology and MicrobiologyInstitute for Plant Genomics and BiotechnologyTexas A&M UniversityCollege StationTX77843USA

**Keywords:** RNA silencing, artificial microRNA, *MIRNA* precursor, *Brachypodium distachyon*, monocot, *Arabidopsis thaliana*, technical advance

## Abstract

Artificial microRNAs (amiRNAs) are used for selective gene silencing in plants. However, current methods to produce amiRNA constructs for silencing transcripts in monocot species are not suitable for simple, cost‐effective and large‐scale synthesis. Here, a series of expression vectors based on *Oryza sativa MIR390* (*OsMIR390*) precursor was developed for high‐throughput cloning and high expression of amiRNAs in monocots. Four different amiRNA sequences designed to target specifically endogenous genes and expressed from *OsMIR390‐*based vectors were validated in transgenic *Brachypodium distachyon* plants. Surprisingly, amiRNAs accumulated to higher levels and were processed more accurately when expressed from chimeric *OsMIR390*‐based precursors that include distal stem–loop sequences from *Arabidopsis thaliana MIR390a (AtMIR390a)*. In all cases, transgenic plants displayed the predicted phenotypes induced by target gene repression, and accumulated high levels of amiRNAs and low levels of the corresponding target transcripts. Genome‐wide transcriptome profiling combined with 5′‐RLM‐RACE analysis in transgenic plants confirmed that amiRNAs were highly specific.

## Significance Statement

A series of amiRNA vectors based on *Oryza sativa MIR390* (*OsMIR390*) precursor were developed for simple, cost‐effective and large‐scale synthesis of amiRNA constructs to silence genes in monocots. Unexpectedly, amiRNAs produced from chimeric *OsMIR390*‐based precursors including *Arabidopsis thaliana MIR390a* distal stem‐loop sequences accumulated elevated levels of highly effective and specific amiRNAs in transgenic *Brachypodium distachyon* plants.

## Introduction

MicroRNAs (miRNAs) are a class of ≈21 nt long endogenous small RNAs that posttranscriptionally regulate gene expression in eukaryotes (Bartel, 2004). In plants, DICER‐LIKE1 processes *MIRNA* precursors with imperfect self‐complementary foldback structures into miRNA/miRNA* duplexes (Bologna and Voinnet, 2014). Typically, one strand of the miRNA duplex is sorted into an ARGONAUTE (AGO) protein according to the identity of the 5′‐terminal nucleotide (nt) of the miRNA (Mi *et al*., 2008; Montgomery *et al*., 2008; Takeda *et al*., 2008) and/or to other sequence or structural properties of the miRNA duplex (Zhu *et al*., 2011; Endo *et al*., 2013; Zhang *et al*., 2014). Plant miRNAs target transcripts with highly complementary sequence through direct AGO‐mediated endonucleolytic cleavage, or through other cleavage‐independent mechanisms (Axtell, 2013).

Artificial miRNAs (amiRNAs) can be produced accurately by modifying the miRNA/miRNA* sequence within a functional *MIRNA* precursor (Alvarez *et al*., 2006; Schwab *et al*., 2006). AmiRNAs have been used in plants to selectively and effectively knockdown reporter and endogenous genes, non‐coding RNAs and viruses (Ossowski *et al*., 2008; Tiwari *et al*., 2014). Recently, cost‐ and time‐effective methods to generate large numbers of amiRNA constructs were developed and validated for eudicot species (Carbonell *et al*., 2014). These included a series of eudicot amiRNA vectors based on *Arabidopsis thaliana MIR390a* (*AtMIR390a*) precursor, whose relatively short distal stem–loop allows the cost‐effective synthesis and cloning of the amiRNA inserts into ‘B/c’ expression vectors (Carbonell *et al*., 2014). In monocots, *OsMIR528* precursor has been used successfully to express amiRNAs for silencing endogenous genes in rice (Warthmann *et al*., 2008; Butardo *et al*., 2011; Chen *et al*., 2012a,b). However, *OsMIR528*‐based cloning methods have not been optimized for efficient generation of monocot amiRNA constructs.

In this report, a series of amiRNA expression vectors for high‐throughput cloning and high‐level expression in monocot species are described and tested. These vectors contain a truncated sequence from *Oryza sativa MIR390* (*OsMIR390*) precursor in a configuration that allows the direct cloning of amiRNAs. *OsMIR390*‐based amiRNAs were generally more accurately processed and accumulated to higher levels in transgenic *Brachypodium distachyon* (Brachypodium) when processed from chimeric precursors (*OsMIR390‐AtL*) containing *Arabidopsis thaliana* (Arabidopsis) *MIR390a* (*AtMIR390a*) distal stem–loop sequences. Functionality of *OsMIR390‐AtL*‐based amiRNAs was confirmed in Brachypodium transgenic plants that displayed the predicted phenotypes, accumulated high levels of amiRNAs and low levels of the corresponding target transcripts. Moreover, genome‐wide transcriptome profiling in combination with 5′‐RLM‐RACE analysis confirmed that the amiRNAs were highly specific. We also describe a cost‐optimized alternative to generate amiRNA constructs for eudicots, as amiRNAs produced from chimeric *AtMIR390a*‐based precursors including *AtMIR390a* basal stem and *OsMIR390* short distal stem–loop sequences are highly expressed, accurately processed, and effective in target gene knockdown in *A. thaliana*.

## Results and Discussion

### AmiRNA vectors based on the *OsMIR390* precursor

**Figure 1:**
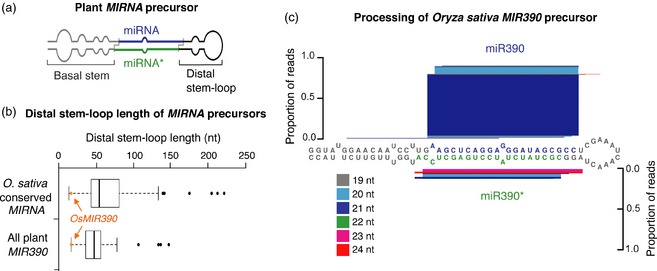
*Oryza sativa MIR390* (*OsMIR390*) is an accurately processed, conserved *MIRNA* precursor with a particularly short distal stem–loop. (a) Diagram of a canonical plant *MIRNA* precursor (adapted from Cuperus *et al*., 2011). miRNA guide and miRNA* strands are highlighted in blue and green, respectively. Distal stem–loop and basal stem regions are highlighted in black and grey, respectively. (b) Distal stem–loop length of *O. sativa* conserved *MIRNA* precursors and of all plant catalogued *MIR390* precursors. Box‐plot showing the distal stem–loop length of *O. sativa* conserved *MIRNA* precursors and all catalogued *MIR390* precursors. The distal stem–loop length of *OsMIR390* is highlighted with an orange dot and indicated with an orange arrow. Outliers are represented with black dots. (c) *OsMIR390* precursor processing diagram. miR390 and miR390* nucleotides are highlighted in blue and green, respectively. Proportion of small RNA reads for the entire *OsMIR390* precursor are plotted as stacked bar graphs. Small RNAs are color‐coded by size. Publicly available small RNA data sets from rice grains, roots, shoots, leaves and inflorescences (Heisel *et al*., 2008; Zhu *et al*., 2008; Johnson *et al*., 2009; Zhou *et al*., 2009; He *et al*., 2010) were analyzed.

Previously, the short *AtMIR390a* precursor was selected as the backbone for high‐throughput cloning of amiRNAs in a series of vectors for eudicot species (Carbonell *et al*., 2014). These vectors allow a zero‐background, oligonucleotide cloning strategy that requires no enzymatic modifications, PCR steps, restriction digestions, or DNA fragment isolation (Carbonell *et al*., 2014). The short distal stem–loop (Figure 1a) of *AtMIR390a* precursor provides a cost advantage by reducing the length of synthetic oligonucleotides corresponding to the amiRNA precursor sequence. To develop a comparable system for monocot species, a search for conserved, short *Oryza sativa* (rice) *MIRNA* (*OsMIRNA*) precursors that could be adapted for amiRNA vectors was done. Rice *MIRNA* precursors were analyzed as they have been subjected to extensive prior analysis (Arikit *et al*., 2013). The distal stem–loop length of 142 *OsMIRNA* precursor sequences (median length = 54 nt, Figure 1b) from 23 conserved miRNA families (Table S1) revealed that the *OsMIR390* precursor was one of the shortest (16 nt). Moreover, *OsMIR390* contains the shortest distal stem–loop of all 51 sequenced *MIR390* precursors from 36 species (median length = 47 nt; Figure 1b and Table S2), including those from maize (*ZmaMIR390a* and *ZmaMIR390b*), sorghum (*SbiMIR390a*) and *B. distachyon* (*BdiMIR390*) with lengths of 137, 148, 134 and 107 nt respectively. The *MIR390* family is among the most deeply conserved miRNA families in plants (Axtell *et al*., 2006; Cuperus *et al*., 2011).

Publicly available small RNA data sets from rice (Heisel *et al*., 2008; Zhu *et al*., 2008; Johnson *et al*., 2009; Zhou *et al*., 2009; He *et al*., 2010) were analyzed to assess the *OsMIR390* precursor processing accuracy. Approximately 70% of reads mapping to the *OsMIR390* foldback correspond to the authentic 21‐nt miR390 guide strand (Figure 1c). Given the short distal stem–loop sequence and relatively accurate precursor processing characteristics, *OsMIR390* was selected as the backbone for amiRNA vector development.

A set of amiRNA cloning vectors based on *OsMIR390* and named ‘*OsMIR390‐B/c*’ (from *OsMIR390*‐*B* *sa*I**/** *c* *cd*B) was developed for rapid cloning of amiRNAs (Figure S1 and Table 1). *OsMIR390‐B/c* vectors include a truncated *OsMIR390* precursor sequence whose miRNA/distal stem–loop/amiRNA* region was substituted by a DNA cassette containing the counter‐selectable *ccd*B gene (Bernard and Couturier, 1992) flanked by two *Bsa*I sites. AmiRNA inserts corresponding to amiRNA/*OsMIR390*‐distal‐stem–loop/amiRNA* sequences are synthesized using two overlapping and partially complementary 60‐base oligonucleotides (Figure S2). Forward and reverse oligonucleotides must have 5′‐CTTG and 5′‐CATG overhangs, respectively, for direct cloning into *OsMIR390*‐based vectors (Figure S2).

**Table 1:** *OsMIR390‐BsaI/ccdB* (‘B/c’) vectors for direct cloning of amiRNAs

Vector	Bacterial antibiotic resistance	Plant antibiotic resistance	GATEWAY use	Backbone	Promoter	Terminator	Plant species tested
*pENTR‐OsMIR390‐B/c*	Kanamycin	–	Donor	*pENTR*	–	–	–
*pMDC123SB‐OsMIR390‐B/c*	Kanamycin	BASTA	–	*pMDC123*	*CaMV* 2x35S	*nos*	*N. benthamiana*
*pMDC32B‐OsMIR390‐B/c*	Kanamycin Hygromycin	Hygromycin	–	*pMDC32*	*CaMV* 2x35S	*nos*	*N. benthamiana* *B. distachyon*
*pH7WG2B‐OsMIR390‐B/c*	Spectinomycin	Hygromycin	–	*pH7WG2*	*Os Ubiquitin*	*CaMV*	*B. distachyon*

*OsMIR390‐B/c* vectors include *pMDC32B‐OsMIR390‐B/c*,* pMDC123SB‐OsMIR390‐B/c* and *pH7WG2B‐OsMIR390‐B/c* plant expression vectors, each of which contains an exclusive combination of regulatory sequences and plant and bacterial antibiotic resistance genes (Figure S1 and Table 1). Additionally, a GATEWAY‐compatible entry vector named *pENTR‐OsMIR390‐B/c* was developed for rapid amiRNA insert cloning and posterior recombination into the GATEWAY expression vector of choice (Figure S1 and Table 1).

### High accumulation of amiRNAs derived from chimeric precursors in Brachypodium calli

**Figure 2:**
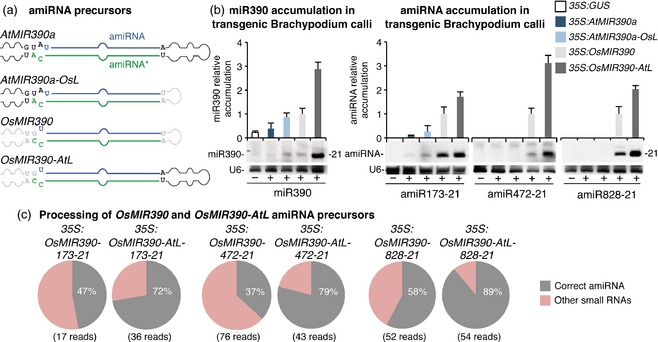
Comparative analysis of accumulation and processing of several amiRNAs produced from *AtMIR390a*,* AtMIR390a‐OsL*,* OsMIR390* and *OsMIR390‐AtL* precursors in Brachypodium transgenic calli. (a) Diagrams of *AtMIR390a*,* AtMIR390a‐OsL*,* OsMIR390* and *OsMIR390‐AtL* precursors. Nucleotides corresponding to the miRNA guide strand are in blue, and nucleotides of the miRNA* strand are in green. Other nucleotides from *AtMIR390a* and *OsMIR390* precursors are in black and grey, respectively. Shapes of *AtMIR390a* and *OsMIR390* precursors are in black and grey, respectively. (b) Accumulation of miR390 (left) and of several 21‐nucleotide amiRNAs (right) expressed from the *AtMIR390a*,* AtMIR390a‐OsL*,* OsMIR390* or *OsMIR390‐AtL* precursors in Brachypodium transgenic calli. Mean (*n* = 3) relative amiRNA levels + standard deviation (SD) when expressed from the *OsMIR390* (light grey, amiRNA level = 1.0). Only one blot from three biological replicates is shown. U6 RNA blot is shown as loading control. (c) Processing analysis of *OsMIR390* and *OsMIR390‐AtL* amiRNA precursors. Pie charts show the percentage of reads corresponding to accurately processed 21‐nt mature amiRNAs (grey sectors) or to other small RNAs (pink sectors).

To test amiRNA expression from *OsMIR390* precursors, transformed *B. distachyon* calli containing amiRNA constructs expressing miR390 or modified versions of several miRNAs from Arabidopsis (amiR173‐21, amiR472‐21 or amiR828‐21) (Cuperus *et al*., 2010) were analyzed (Figure 2a). In addition, the same amiRNAs were expressed from a chimeric precursor (*OsMIR390‐AtL*) composed of the *OsMIR390* basal stem and *AtMIR390a* distal stem–loop (Figures 2a and S3). Each amiRNA was also expressed from the reciprocal chimeric precursors (*AtMIR390a‐OsL*) containing the *AtMIR390a* basal stem and *OsMIR390* distal stem–loop (Figures 2a and S4). A *35S:GUS* construct expressing the ß‐glucuronidase transcript was used as negative control.

Surprisingly, miR390 accumulated to highest levels when expressed from the chimeric *OsMIR390‐AtL* precursor compared with each of the other three precursors (*P* ≤ 0.001 for all pairwise *t*‐test comparisons; Figure 2b). Moreover, each amiRNA expressed from *OsMIR390‐AtL* chimeric precursors also accumulated to significantly higher levels when compared with the other precursors (*P* < 0.026 for all pairwise *t*‐test comparisons; Figure 2b). miR390 and each amiRNA derived from authentic *AtMIR390a* or chimeric *AtMIR390a‐OsL* precursors accumulated to low or non‐detectable levels, indicating that the *AtMIR390a* stem is suboptimal for the accumulation and/or processing of amiRNAs in Brachypodium.

To assess the accuracy of precursor processing, small RNA libraries from samples expressing *OsMIR390‐AtL*‐based amiRNAs were prepared and sequenced (Figure 2c). For comparative purposes, small RNA libraries from samples containing amiRNAs produced from authentic *OsMIR390* precursors were also analyzed. In each case, the majority of reads mapping to the chimeric *OsMIR390‐AtL* precursors corresponded to correctly processed 21 nt amiRNAs (Figure 2c). In contrast, processing of authentic *OsMIR390* precursors including amiRNA sequences was less accurate, as revealed in each case by a lower proportion of reads corresponding to correctly processed sequences (Figure 2c).

### Gene silencing in Brachypodium and Arabidopsis by amiRNAs derived from chimeric precursors

**Figure 3:**
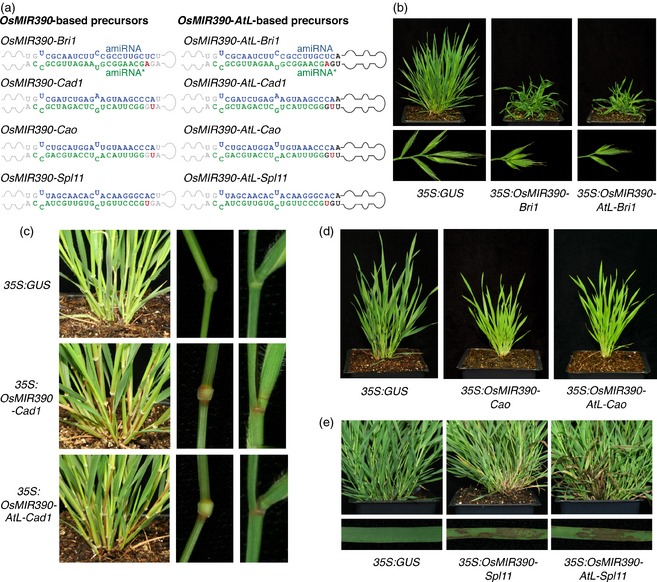
Functionality of amiRNAs produced from authentic *OsMIR390‐* or chimeric *OsMIR390‐AtL*‐based precursors in Brachypodium T0 transgenic plants. (a) *OsMIR390*‐ and *OsMIR390‐AtL*‐based precursors containing Bri1‐, Cad1‐, Cao and Spl11‐amiRNAs. Nucleotides corresponding to the miRNA guide and miRNA* strands are in blue and green, respectively; nucleotides from *AtMIR390a* or *OsMIR390* precursors are in black or grey, respectively, except those that were modified to preserve authentic *AtMIR390a* or *OsMIR390* precursor secondary structures (in red). (b–e) Representative images of plants expressing amiRNAs from *OsMIR390‐AtL* or *OsMIR390* precursors, or the control construct. (b) Adult control plant (left), or plants expressing *35S:OsMIR390‐Bri1* (center) or *35S:OsMIR390‐AtL‐Bri1* (right). (c) Adult control plant (left), or plants expressing *35S:OsMIR390‐Cad* (center) or *35S:OsMIR390‐AtL‐Cad1* (bottom). (d) Adult control plant (left), or plants expressing *35S:OsMIR390‐Cao* (center) or *35S:OsMIR390‐AtL‐Cao* (right). (e) Adult control plant (left), or plants expressing *35S:OsMIR390‐Spl11* (center) or *35S:OsMIR390‐AtL‐Spl11* (right).

To assess the functionality of *OsMIR390‐AtL*‐derived amiRNAs in silencing target transcripts in Brachypodium, *BRASSINOSTEROID‐INSENSITIVE 1* (*BdBRI1)*,* CINNAMYL ALCOHOL DEHYDROGENASE 1* (*BdCAD1*), *CHLOROPHYLLIDE A OXYGENASE* (*BdCAO*) and *SPOTTED LEAF 11* (*BdSPL11*) gene transcripts were targeted by amiRNAs expressed from the chimeric *OsMIR390‐AtL* and from authentic *OsMIR390* precursors (Figure 3a). The sequences for amiR‐BdBri1, amiR‐BdCad1, amiR‐BdCao and amiR‐BdSpl11 (Figure S5) were designed using the ‘P‐SAMS amiRNA Designer’ tool (http://p-sams.carringtonlab.org). Plants expressing *35S:GUS* were used as negative controls. Phenotypes of transgenic plants, amiRNA accumulation, processing of amiRNA precursors, and target transcript accumulation were analyzed in Brachypodium T0 transgenic lines.

Sixteen out of 20 and 11 out of 17 transgenic lines containing *35S:OsMIR390‐AtL‐Bri1* or *35S:OsMIR390‐Bri1*, respectively, which were predicted to have brassinosteroid signalling defects, had reduced height and altered architecture (Figures 3b and S6 and Table S3). Most organs, particularly leaves, exhibited a contorted phenotype since the earliest stages of development (Figure 3b). Inflorescences had reduced size (Figure 3b), and contained smaller seeds compared to control lines (Figure S6). AmiR‐BdBri1‐induced phenotypes were similar to those described for the Brachypodium *bri1* T‐DNA mutants from the BrachyTAG collection (Thole *et al*., 2012). These phenotypes are consistent with the expectation of plants with brassinosteroid signalling defects (Zhu *et al*., 2013). All 27 transgenic lines containing *35S:OsMIR390‐AtL‐Cad1*, and 52 out of 55 lines including *35S:OsMIR390‐Cad1*, exhibited reddish coloration of lignified tissues such as tillers, internodes and nodes (Figure 3c and Table S3), as expected from *Cad1* knockdown and loss of function mutant analyses (Bouvier d'Yvoire *et al*., 2013; Trabucco *et al*., 2013).

Each of 27 *35S:OsMIR390‐AtL‐Cao*‐expressing plants, and 12 of 12 of *35S:OsMIR390‐Cao*‐expressing plants exhibited light green color compared with control plants (Figure 3d and Table S3), as expected due to reduction in chlorophyllide *a* to *b* conversion during chlorophyll *b* synthesis (Tanaka *et al*., 1998; Oster *et al*., 2000; Philippar *et al*., 2007). Biochemical analysis of chlorophyll content in transgenic lines confirmed that chlorophyll *b* content in *35S:OsMIR390‐AtL‐Cao* and *35S:OsMIR390‐Cao* lines was reduced to approximately 57 and 67%, respectively, compared with levels measured in control plants (Figure S7). Carotenoid content was also notably reduced (to almost 50%) in lines expressing amiR‐BdCao from chimeric or authentic precursors (Figure S7), as observed before in Arabidopsis *cao* mutants (Philippar *et al*., 2007). Finally, 39 of 43 transgenic lines containing *35S:OsMIR390‐AtL‐Spl11*, and 22 of 24 *35S:OsMIR390‐Spl11*‐expressing plants displayed a spontaneous cell death phenotype characterized by the development of necrotic lesions in leaves (Figure 3e). This was consistent with expectations based on phenotypes of *SPL11*‐knockdown amiRNA rice lines (Zeng *et al*., 2004). Phenotypes induced by all four sets of amiRNAs were heritable in self‐pollinated T1 plants expressing *OsMIR390*‐ or *OsMIR390‐AtL*‐based amiRNA precursors from *pMC32B* vectors containing 35S regulatory sequences (Table S4).

**Figure 4:**
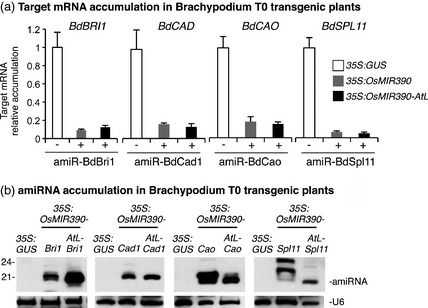
amiRNA and target mRNA accumulation analysis in Brachypodium T0 transgenic plants. (a) Mean relative level ± standard error (SE) of *B. distachyon BdBRI1*,* BdCAD1*,* BdCAO* and *BdSPL11* mRNAs after normalization to *BdSAMDC*,* BdUBC*,* BdUBI4* and *BdUBI10*, as determined by quantitative real‐time RT‐PCR (*35S:GUS *= 1.0 in all comparisons). (b) Accumulation of amiRNAs in Brachypodium transgenic plants. In each blot the amiRNA accumulation of a single independent transgenic line per construct is analyzed. U6 RNA blot is shown as a loading control.

Quantitative real‐time RT‐PCR (RT‐qPCR) assays were used to measure the accumulation of amiRNA‐target transcripts in Brachypodium transgenic lines expressing *OsMIR390‐AtL*‐ or *OsMIR390*‐based amiRNAs. All target transcripts were expressed to significantly reduced levels compared with control plants (*P* < 0.005 for all pairwise *t*‐test comparisons, Figure 4a) in transgenic lines expressing the specific amiRNA. No significant differences were observed in target mRNA levels between lines expressing *OsMIR390‐AtL*‐ or *OsMIR390*‐based amiRNAs.

AmiR‐BdBri1, amiR‐BdCao and amiR‐BdSpl11 produced from chimeric *OsMIR390‐AtL* precursors were also expressed using *pH7WG2B‐*based constructs that contain the rice ubiquitin (UBI) regulatory sequences. Each of the three UBI promoter‐driven amiRNAs induced the expected phenotypes in a relatively high proportion of Brachypodium T0 lines (Table S3), and in the one case tested (amiR‐BdSpl11), phenotypes were heritable in the T1 generation (Table S4).

Finally, we tested if the reciprocal chimeric *AtMIR390a‐OsL* precursor could be used to express amiRNAs efficiently in eudicots. The synthesis of *AtMIR390a‐OsL*‐based constructs requires shorter oligonucleotides than the generation of *AtMIR390a*‐based constructs, and therefore would be a further cost‐optimized alternative. As shown in *Nicotiana benthamiana* and Arabidopsis assays, *AtMIR390‐OsL* precursors are accurately processed (Appendix S1 and Figures S8–S10). Indeed, amiRNAs produced from chimeric *AtMIR390a‐OsL* precursors are highly expressed, accurately processed and highly effective in target gene knockdown in T1 Arabidopsis transgenic plants (Appendix S1, Figures S9–S11 and Table S5). Moreover, amiRNA‐induced phenotypes were still obvious in T2 plants confirming the heritability of the effects (Table S6). Therefore, the use of *AtMIR390a‐OsL* precursors may be an attractive alternative to express effective amiRNAs in eudicots in a cost‐optimized manner.

### Accuracy of processing of *OsMIR390* and *OsMIR390‐AtL* chimeric precursors in Brachypodium

The accumulation of each amiRNA from chimeric and *OsMIR390* precursors was analyzed by RNA blot analysis in T0 transgenic lines exhibiting phenotypes induced by amiRNAs (Figure 4b). In most cases, *OsMIR390‐AtL*‐derived amiRNAs accumulated to higher levels and as more uniform RNA species (Figure 4b). AmiRNAs from the *OsMIR390* precursor accumulated to rather low levels (except in transgenic lines containing *35S:OsMIR390‐Cao*) and generally as multiple species (Figure 4b).

**Figure 5:**
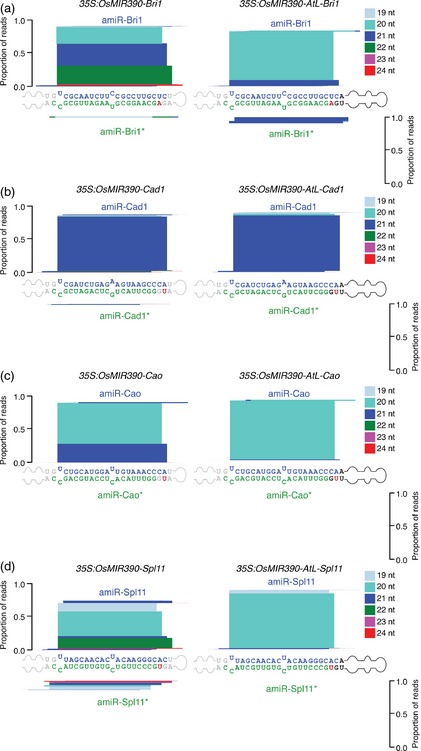
Mapping of amiRNA reads from *OsMIR390‐AtL*‐ or *OsMIR390*‐based precursors expressed in Brachypodium T0 transgenic plants. Analysis of amiRNA and amiRNA* reads in plants expressing (a) amiR‐BdBri1, (b) amiR‐BdCad1, (c) amiR‐BdCao or (d) amiR‐BdSpl11. amiRNA guide and amiRNA* strands are highlighted in blue and green, respectively. Nucleotides from the *AtMIR390a* or *OsMIR390* precursors are in black and grey, respectively, except those that were modified to preserve the corresponding authentic precursor secondary structure (in red). Proportion of small RNA reads are plotted as stacked bar graphs. Small RNAs are color‐coded by size.

Small RNA libraries from transgenic lines expressing amiRNAs from chimeric *OsMIR390‐AtL* or authentic *OsMIR390* precursors were prepared to further analyze processing and accumulation of the amiRNA species (Figure 5). Three of the four amiRNAs produced from chimeric *OsMIR390‐AtL* precursors accumulated predominantly as 20‐nt species (Figure 5a,c,d); only amiR‐BdCad1 accumulated mainly as a 21 nt RNA (Figure 5b). Processing of authentic *OsMIR390* precursors generally resulted in a high proportion of small RNAs of diverse sizes, except for *OsMIR390‐Cad1* precursors (Figure 5).

The reasons explaining the accumulation of *OsMIR390a‐AtL*‐based amiRNAs that are 1 nt‐shorter than expected are not clear. AmiRNAs shorter than expected and differing on their 3′ end were also described using *AtMIR319a* precursors in Arabidopsis (Schwab *et al*., 2006). Importantly, a recent study has shown that amiRNA efficacy is not affected by the loss of the base‐pairing at the 5′ end of the target site (Liu *et al*., 2014). Regardless, the inaccurate processing of an amiRNA precursor leading to the accumulation of diverse small RNA populations could conceivably induce undesired off‐target effects. This potential complication argues against using authentic *OsMIR390* precursors to express amiRNAs in Brachypodium and possibly other monocot species.

Reads from the amiRNA* strands from each of the *OsMIR390* and *OsMIR390‐AtL*‐derived precursors were under‐represented, relative to the amiRNA strands (Figure 5). The rational P‐SAMS design tool uniformly specifies an amiRNA* strand containing an AGO‐non‐preferred 5′ G residue, which likely promotes amiRNA* degradation.

### High specificity of amiRNA derived from chimeric precursors in Brachypodium

**Figure 6:**
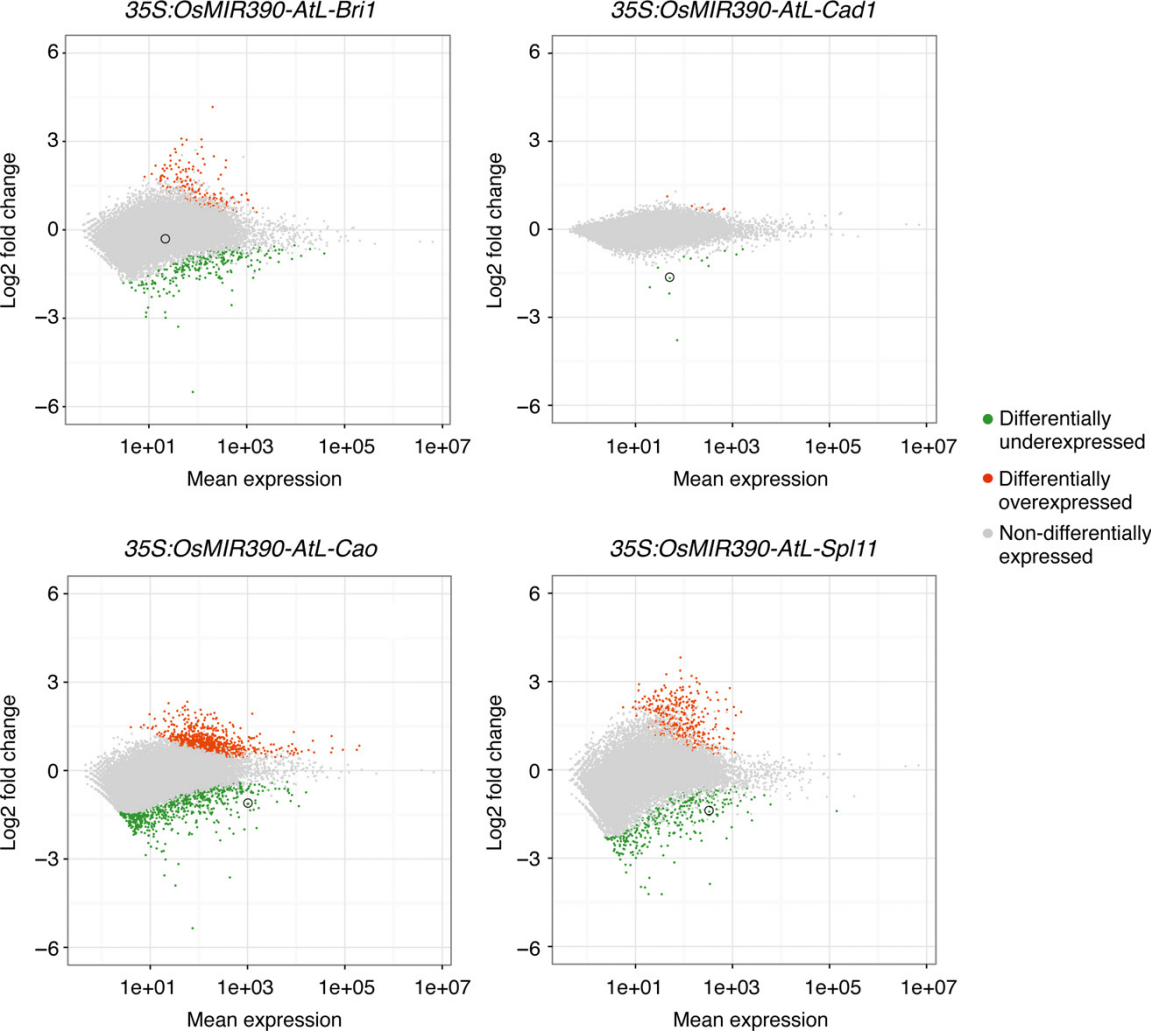
Transcriptome analysis of transgenic Brachypodium plants expressing amiRNAs from chimeric *OsMIR390‐AtL* precursors. MA plots show log2 fold change versus mean expression of genes for each *35S:OsMIR390‐AtL* amiRNA line compared with the control lines (*35S:GUS*). Green, red and grey dots represent differentially underexpressed, differentially overexpressed or non‐differentially expressed genes, respectively, in each amiRNA versus control comparison. The position of expected amiRNA targets is indicated with a circle.

To assess amiRNA‐target specificity at a genome‐wide level, transcript libraries from control (*35S:GUS*) and amiRNA‐expressing lines were generated and analyzed. Only lines expressing amiRNAs from the more accurately processed *OsMIR390‐AtL* precursors were analyzed. Differential gene expression analyses were done by comparing, in each case, the transcript libraries obtained from four independent control lines with those obtained from four independent amiRNA‐expressing lines exhibiting the expected phenotypes. In total, 494, 1847 and 818 genes were differentially expressed in plants expressing amiR‐BdBri1, amiR‐BdCao and amiR‐BdSpl11, respectively (Figure 6 and Data S1). In contrast, only 21 genes were differentially expressed in plants expressing amiR‐BdCad1 (Figure 6 and Data S1). The high number of differentially expressed genes in amiR‐BdBri1‐, amiR‐BdCao‐ and amiR‐BdSpl11‐expressing lines may reflect the complexity of the corresponding targeted gene pathways involving hormone signalling, photosynthesis and cell death/pathogen resistance respectively. As expected, *BdCAD1*,* BdCAO* and *BdSPL11* were differentially underexpressed in plants expressing amiR‐BdCad1, amiR‐BdCao and amiR‐BdSpl11, respectively (*q* < 0.01, Wald test) (Figure 6 and Data S1). However, *BdBRI1* was not called as differentially expressed (*q* = 0.42, Wald test) (Figure 6 and Data S1) despite being notably downregulated in *35S:OsMIR390‐AtL‐Bri1* plants as shown by RT‐qPCR analysis (Figure 4a). Because the power of statistical tests involving count data decreases with lower count numbers (Rapaport *et al*., 2013), this result could be explained by the low accumulation of *BdBRI1* even in control plants (Figure S12 and Data S2). Therefore, the differential expression analysis on RNA‐Seq data approach may not be appropriate to evaluate the differential expression of genes with genuine low expression and/or low coverage, as suggested before (Rapaport *et al*., 2013).

To assess potential off‐target effects of the amiRNAs, targetfinder (Fahlgren and Carrington, 2010) was used to generate a genome‐wide list of potential candidate targets that share relatively high sequence complementarity with each amiRNA. targetfinder ranks the potential amiRNA targets based on a Target Prediction Score (TPS) assigned to each amiRNA‐target interaction. Scores range from 1 to 11, that is, from highest to lowest levels of sequence complementarity between the small RNA and putative target RNA. Indeed, when designing amiRNAs with the ‘P‐SAMS amiRNA Designer’ tool, ‘optimal’ amiRNAs are selected when: (i) their interaction with the desired target has a TPS = 1; and (ii) no other amiRNA‐target interactions have a TPS < 4. Therefore, direct off‐target effects with amiRNAs described here can only occur through amiRNA‐target RNA interactions with a TPS in the [4, 11] interval. It was hypothesized that off‐target effects, if due to base‐pairing between amiRNAs and the affected transcripts, would be reflected by the presence of differentially underexpressed genes corresponding to target RNAs with lower TPS scores in the [4, 11] interval. Therefore, we next analyzed for all targetfinder‐predicted targets for each amiRNA if their corresponding genes were differentially underexpressed in amiRNA‐expressing lines versus controls.

**Figure 7:**
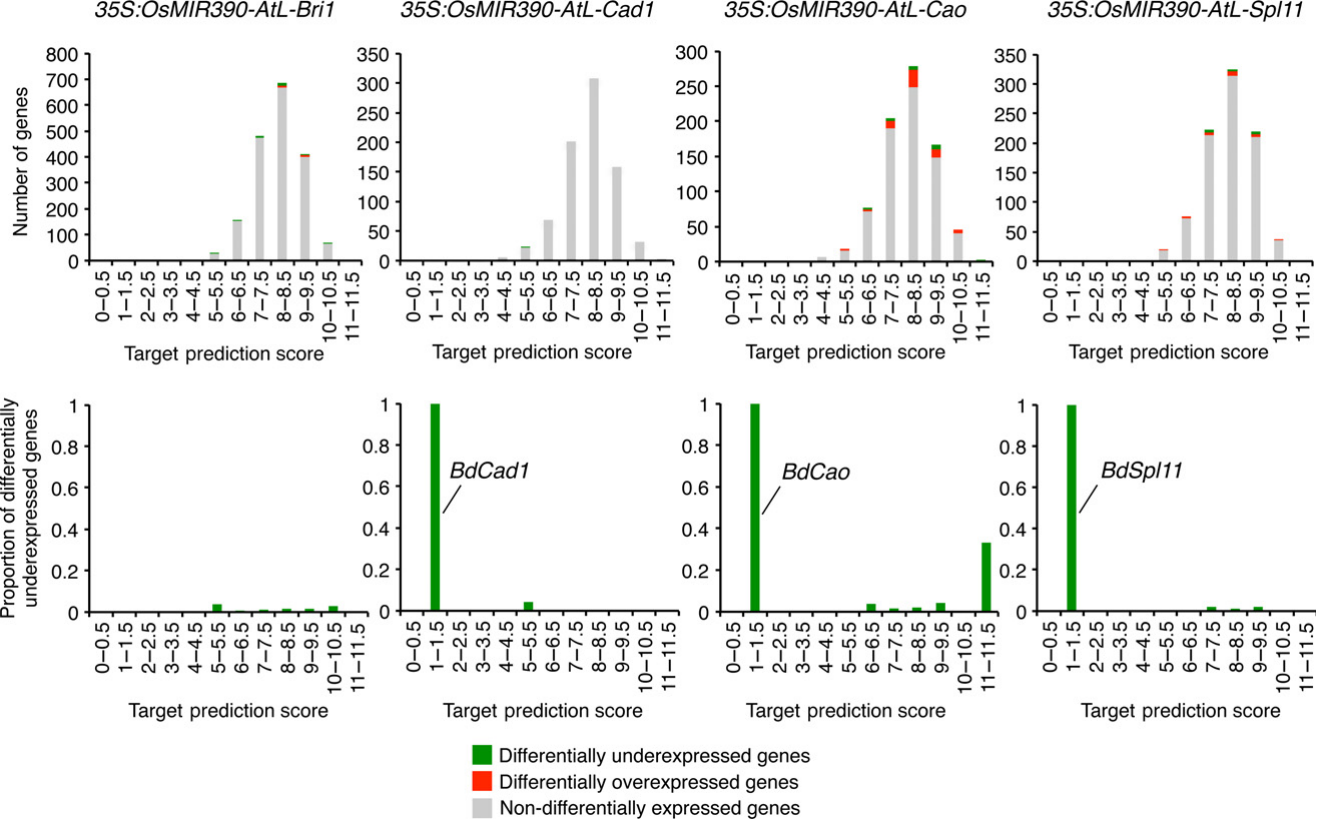
Differential expression analysis of targetfinder‐predicted off‐targets for each amiRNA versus control comparison. Histograms show the total number of genes (top panels) or the proportion of differentially underexpressed genes (bottom panels) in each target prediction score bin. Green, red and grey bars represent differentially underexpressed, differentially overexpressed or non‐differentially expressed genes, respectively. In bottom panels, the name of the expected target gene is indicated when the target gene is the only gene differentially underexpressed in the corresponding bin.

As expected from P‐SAMS design, *BdCad1*,* BdCao* and *BdSpl11* were the only genes differentially underexpressed in the [1, 4] TPS interval in plants expressing amiR‐BdCad1, amiR‐BdCao and amiR‐BdSpl11, respectively (Figure 7, Data S3). On the other hand, 2958, 1290, 1528 and 1533 genes corresponded to target RNAs with calculated TPS scores in the [4, 11] interval in targetfinder analyses including amiR‐BdBri1, amiR‐BdCad1, amiR‐BdCao and amiR‐BdSpl11, respectively (Figure 7). In all cases, the number of differentially underexpressed genes corresponding to predicted targets with a TPS in the [4, 11] interval was low (Figure 7, upper panels). Moreover, in each of the four cases the proportion of differentially underexpressed genes among targetfinder‐predicted targets was also low in the [4, 11] TPS interval (Figure 7, bottom panels). Indeed, in this same interval, 0.84, 1.31 and 0.78% of the genes were differentially underexpressed in amiR‐BdBri1‐, amiR‐BdCao‐, and amiR‐BdSpl11‐expressing lines, respectively. In each case, this percentage was lower than the percentage of differentially underexpressed genes from transcripts with a TPS not included in the [4, 11] interval in the same samples (1.12, 3.74 and 1.55% respectively). In amiR‐BdCad‐expressing lines, although the percentage of genes differentially expressed in the [4, 11] interval (0.07%) was higher compared to the percentage of genes differentially underexpressed in the [4, 11] interval (0.04%), this difference was not statistically significant (*P* = 0.45, Fisher exact test). Together, these results indicate that globally targetfinder‐predicted targets were not preferentially downregulated in the amiRNA‐expressing lines.

**Figure 8:**
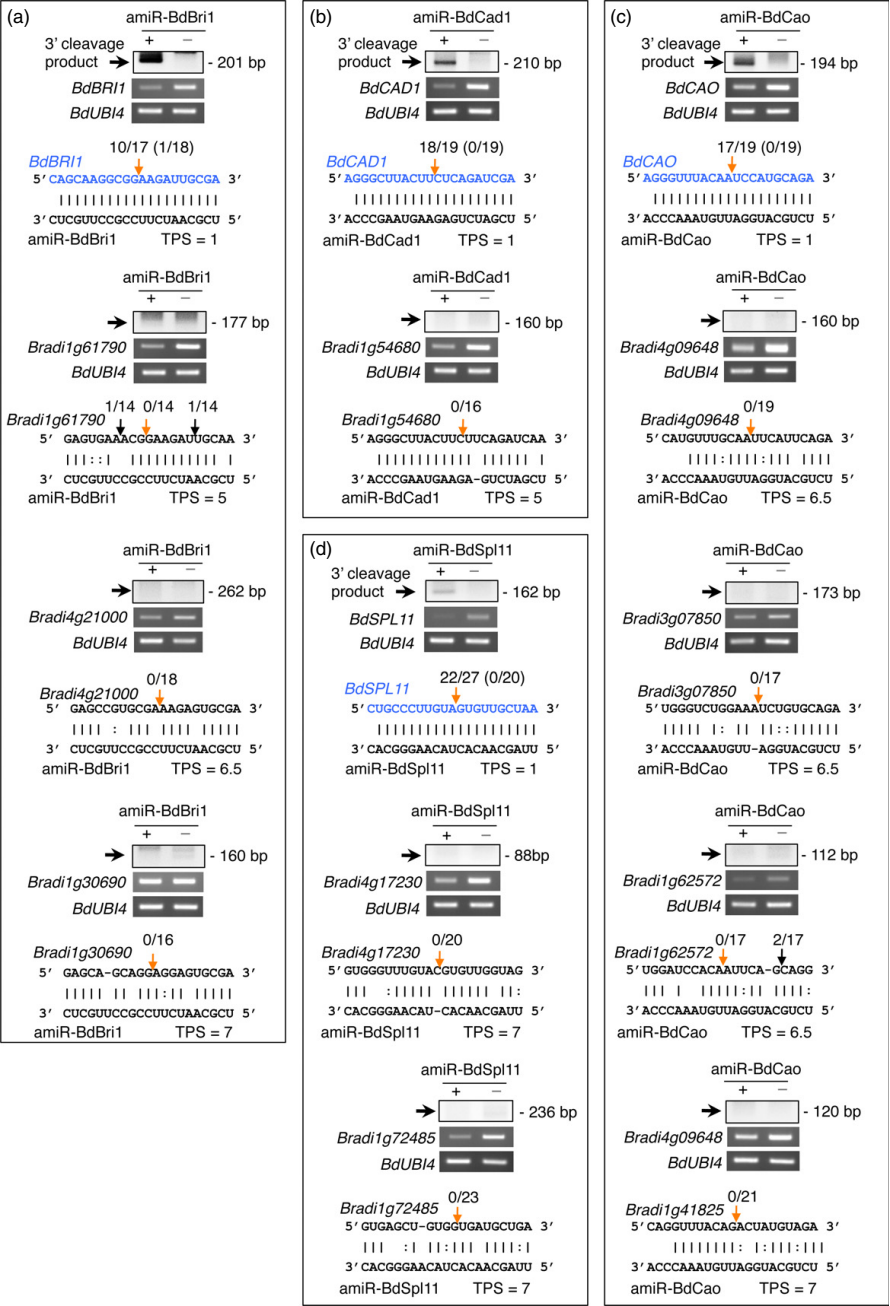
5′ RLM‐RACE mapping of target and potential off‐target cleavage guided by amiRNAs in plants expressing (a) amiR‐BdBri1, (b) amiR‐BdCad1, (c) amiR‐BdCao and (d) amiR‐BdSpl11. At the top of each panel, ethidium bromide‐stained gels show 5′‐RLM‐RACE products corresponding to the 3′ cleavage product from amiRNA‐guided cleavage (top gel), and RT‐PCR products corresponding to the gene of interest (middle gel) or control *BdUBI4* gene (bottom gel). The position and size of the expected amiRNA‐based 5′‐RLM‐RACE products are indicated. At the bottom of each panel, the predicted base‐pairing between amiRNAs and prospective target RNAs is shown. The sequence and the name of authentic target mRNAs are in blue. For each authentic or predicted target mRNA, the expected amiRNA‐based cleavage site is indicated by an orange arrow. Other sites are indicated with a black arrow. The proportion of cloned 5′‐RLM‐RACE products at the different cleavage sites is shown for amiRNA‐expressing lines, with that of control plants expressing *35S:GUS* shown in brackets. TPS refers to ‘Target Prediction Score’.

Next, we used 5′‐RLM‐RACE to test for amiRNA‐directed off‐target cleavage of under‐represented transcripts. This analysis detects 3′ cleavage products expected from small RNA‐guided cleavage events. Only targetfinder‐predicted targets with a TPS ≤ 7 were included in the analysis, as targets with higher score are not considered likely to be cleaved, according to previous studies (Addo‐Quaye *et al*., 2008). For all specific targets, 3′ cleavage products of the expected size were detected in samples expressing the corresponding amiRNA, but not in control samples expressing *35S:GUS* (Figure 8). Sequencing analysis confirmed that the majority of sequences comprising these products, in each case, contained a canonical 5′ end position predicted for small RNA‐guided cleavage (Figure 8). In contrast, for all potential off‐target transcripts, no obvious amiRNA‐guided cleavage products were detected in either amiRNA‐expressing or *35S:GUS* lines (Figure 8). Additionally, sequencing analysis failed to detect even low‐level amiRNA‐guided cleavage products among potential off‐targets (Figure 8).

High amiRNA specificity was previously indicated for *AtMIR319a*‐derived amiRNAs in Arabidopsis based on genome‐wide expression profiling (Schwab *et al*., 2006). However, a recent and systematic processing analysis of *AtMIR319a*‐based amiRNA precursors in petunia (Guo *et al*., 2014) showed that multiple small RNA variants are generated from different regions of the precursor, and that many of these small RNAs meet the required criteria for amiRNA design (Schwab *et al*., 2006). Here, the fact that chimeric *OsMIR390‐AtL* precursors produce high levels of accurately processed amiRNAs not only in Brachypodium (Figures 2, 4 and 5) but also in a eudicot species such as *N. benthamiana* (Figure S8), strongly suggests that these precursors will be functional in a wide range of species.

## Conclusions

We have developed and validated a series of expression vectors based on the *OsMIR390* precursor for high‐throughput cloning and high expression of amiRNAs in monocots. *OsMIR390‐B/c*‐based vectors allow the direct cloning of amiRNAs in a zero‐background strategy that does not require oligonucleotide modifications, PCRs, restriction digestions, or isolation of DNA fragments. Thus, *OsMIR390‐B/c*‐based vectors are particularly attractive for generating large‐scale amiRNA construct libraries for silencing genes in monocots.

‘P‐SAMS amiRNA Designer’ tool was used to design four different amiRNAs, each of which was aimed to target specifically one Brachypodium gene transcript. We show that chimeric *OsMIR390‐AtL* precursors including *OsMIR390* basal stem and *AtMIR390a* distal stem–loop were processed more accurately, and the resulting amiRNAs generally accumulated to higher levels than amiRNAs derived from authentic *OsMIR390* precursors in Brachypodium transgenic plants. Each P‐SAMS‐designed amiRNA induced the expected phenotypes, and specifically decreased expression of the expected target gene. Chimeric *OsMIR390‐AtL* precursors designed using P‐SAMS, therefore, are likely to be highly effective and specific in silencing genes in monocot species.

## Experimental Procedures

### Plant materials and growth conditions

*Arabidopsis thaliana* Col‐0 and *N. benthamiana* plants were grown as described (Carbonell *et al*., 2014). *Brachypodium distachyon* 21‐3 plants were grown in a chamber under long day conditions (16/8 h photoperiod at 200 μmol m^−2^ s^−1^) and 24°C/18°C temperature cycle.

*Arabidopsis thaliana* plants were transformed and grown as described (Carbonell *et al*., 2014). Embryogenic calli from *B. distachyon* 21‐3 plants were transformed as described (Vogel and Hill, 2008). Photographs of plants were taken as described (Carbonell *et al*., 2014).

### DNA constructs

*pENTR‐OsMIR390‐BsaI* construct was generated by ligating into pENTR (Life Technologies; http://www.lifetechnologies.com) the DNA insert resulting from the annealing of oligonucleotides BsaI‐OsMIR390‐F and BsaI‐OsMIR390‐R. Rice ubiquitin 2 promoter and maize ubiquitin promoter‐hygromycin cassettes were transferred into the GATEWAY binary destination vector *pH7WG2* (Karimi *et al*. 2002) to generate *pH7WG2‐OsUbi. pH7WG2‐OsMIR390‐BsaI*,* pMDC123SB‐OsMIR390‐BsaI* and *pMDC32‐OsMIR390‐BsaI* were obtained by LR recombination between *pENTR‐OsMIR390‐BsaI* and *pH7WG2‐OsUbi*,* pMDC32B* (Carbonell *et al*., 2014) and *pMDC123SB* (Carbonell *et al*., 2014), respectively. A modified *ccd*B cassette (Carbonell *et al*., 2014) was inserted between the *Bsa*I sites of *pENTR‐OsMIR390‐BsaI*,* pMDC123SB‐OsMIR390‐BsaI*,* pMDC32B‐OsMIR390‐BsaI* and *pH7WG2‐OsMIR390‐BsaI* to produce *pENTR‐OsMIR390‐B/c*,* pMDC123SB‐OsMIR390‐B/c*,* pMDC32B‐OsMIR390‐B/c* and *pH7WG2‐OsMIR390‐B/c*, respectively. Finally, an undesired *Bsa*I site was disrupted in *pH7WG2‐OsMIR390‐B/c* to generate *pH7WG2B‐OsMIR390‐B/c*. The sequences of the *OsMIR390‐B/c*‐based amiRNA vectors are in Appendix S2. The following amiRNA vectors for monocots are available from Addgene (http://www.addgene.org/): *pENTR‐OsMIR390‐B/c* (Addgene plasmid 61468), *pMDC32B‐OsMIR390‐B/c* (Addgene plasmid 61467) *pMDC123SB‐OsMIR390‐B/c* (Addgene plasmid 61466) and *pH7WG2B‐OsMIR390‐B/c* (Addgene plasmid 61465). *pMDC32B‐AtMIR390a‐B/c* (Addgene plasmid 51776) was described before (Carbonell *et al*., 2014).

AmiRNA constructs including *pMDC32B‐AtMIR390a‐OsL‐173‐21*,* pMDC32B‐AtMIR390a‐OsL‐472‐21*,* pMDC32B‐AtMIR390a‐OsL‐828‐21*,* pMDC32B‐AtMIR390a‐OsL‐Ch42*,* pMDC32B‐AtMIR390a‐OsL‐Ft*,* pMDC32B‐AtMIR390a‐OsL‐Trich*,* pMDC32B‐OsMIR390*,* pMDC32B‐OsMIR390‐AtL*,* pMDC32B‐OsMIR390‐173‐21*,* pMDC32B‐OsMIR390‐173‐21‐AtL*,* pMDC32B‐OsMIR390‐472‐21*,* pMDC32B‐OsMIR390‐AtL‐472‐21*,* pMDC32B‐OsMIR390‐828‐21*,* pMDC32B‐OsMIR390‐AtL‐828‐21*,* pMDC32B‐OsMIR390‐Bri1*,* pMDC32B‐OsMIR390‐AtL‐Bri1*,* pMDC32B‐OsMIR390‐Cao*,* pMDC32B‐OsMIR390‐AtL‐Cao*,* pMDC32B‐OsMIR390‐Cad1*,* pMDC32B‐OsMIR390‐AtL‐Cad1*,* pMDC32B‐OsMIR390‐Spl11*,* pMDC32B‐OsMIR390‐AtL‐Spl11*,* pH7WG2B‐OsMIR390‐Bri1‐AtL*,* pH7WG2B‐OsMIR390‐Cao‐AtL*, and *pH7WG2B‐OsMIR390‐Spl11‐AtL* were generated as detailed in the following section. Control construct *pH7WG2‐GUS* was generated by LR recombination between *pENTR‐GUS* (Life technologies) and *pH7GW2‐OsUbi. pMDC32‐GUS* construct was used before (Montgomery *et al*., 2008). The sequence of all amiRNA precursors used here are in Appendix S3. The sequence of all oligonucleotides used are in Table S7.

### amiRNA oligonucleotide design and cloning

Sequences of the amiRNAs expressed in *A. thaliana* have been described previously (Schwab *et al*., 2006; Felippes and Weigel, 2009; Liang *et al*., 2012; Carbonell *et al*., 2014). Sequences of both the amiRNAs expressed in Brachypodium and the oligonucleotides used for cloning in *OsMIR390‐B/c* vectors, were designed with the ‘P‐SAMS amiRNA Designer’ tool (http://p-sams.carringtonlab.org). The predicted targets for all the amiRNAs used in this study are shown in Table S8.

The generation of constructs to express amiRNAs from authentic *AtMIR390a* precursors was described before (Carbonell *et al*., 2014). Detailed oligonucleotide design for amiRNA cloning in *OsMIR390*,* OsMIR390‐AtL* and *AtMIR390a‐OsL* precursors is given in Figures S2, S3 and S4, respectively. The amiRNA cloning procedure is described in Appendix S4. The name and sequence of the oligonucleotides used for cloning amiRNA sequences are in Table S7.

### Transient expression assays

*N. benthamiana* leaves were agroinfiltrated with *A. tumefaciens* GV3101 strain as described (Carbonell *et al*., 2014).

### RNA blot assays

Total RNA extraction from Arabidopsis, Brachypodium or *N. benthamiana* and subsequent RNA blot assays were done as described (Cuperus *et al*., 2010). Table S7 includes the name and sequences of the oligonucleotides used as probes in small RNA blots.

### Quantitative real‐time RT‐qPCR

RT‐qPCR reactions and analyses were done as described (Carbonell *et al*., 2014). The oligonucleotides used for RT‐qPCR are listed in Table S7 (and are named with the prefix ‘q’). The expression levels of target transcripts were calculated relative to four *A. thaliana* (*AtACT2*,* AtCPB20*,* AtSAND* and *AtUBQ10*) or *B. distachyon* (*BdSAMDC*,* BdUBC18*,* BdUBI4* and *BdUBI10*) reference genes as described (Carbonell *et al*., 2014).

### 5′‐RLM‐RACE

5′ RNA ligase‐mediated rapid amplification of cDNA ends (5′‐RLM‐RACE) was done using the GeneRacer^™^ kit (Life Technologies) but omitting the dephosphorylation and decapping steps. Total RNA (2 μg) was ligated to the GeneRacer RNA Oligo Adapter. The GeneRacer Oligo dT primer was then used to prime first strand cDNA synthesis in reverse transcription reaction. An initial PCR was done by using the GeneRacer 5′ and 3′ primers. The 5′ end of cDNA specific to each mRNA was amplified with the GeneRacer 5′ Nested primer and a gene specific reverse primer. For each gene, control PCR reactions were done using gene specific forward and reverse primers. Oligonucleotides used are listed in Table S7. 5′‐RLM‐RACE products were treated as described (Cuperus *et al*., 2010).

### Chlorophyll and carotenoid extraction and analysis

Pigments from Brachypodium leaf tissue (40 mg of fresh weight) were extracted with 5 ml 80% (v/v) acetone in the dark at room temperature for 24 h, and centrifuged at 1000 ***g*** for 2 min. One hundred microlitres of supernatant was diluted 1:2 with 80% (v/v) acetone and loaded to flat bottom 96‐well plates. Absorbance was measured from 400 to 750 nm wavelengths in a SpectrMax M2 microplate reader (Molecular Devices, Sunnyvale, CA, USA) using the software softmax pro 5 (Molecular Devices). Content in chlorophyll *a*, chlorophyll *b*, and carotenoids was calculated with the following formulas: chlorophyll *a* (mg/L in extract) = 12.21 * Absorbance_663 nm_ − 2.81 * Absorbance_647 nm_; chlorophyll *b* (mg/L in extract) = 20.13 * Absorbance_647 nm_ − 5.03 * Absorbance_663 nm_; carotenoid (mg/L in extract) = [1000 * Absorbance_470 nm_ − 3.27 * chlorophyll *a* (mg/L) − 104 * Chlorophyll *b* (mg/L)]/227.

### Preparation of small RNA libraries

Approximately 50–100 μg of Arabidopsis, Brachypodium *or Nicotiana* total RNA were treated essentially as before (Carbonell *et al*., 2012; Gilbert *et al*., 2014), with the difference that small RNA libraries were barcoded at the amplicon PCR reaction step with the standard 5′PCR oligonucleotide (P5) and an indexed 3′ PCR oligonucleotide (i1‐i8, i10 or i11) (Table S7). Library multiplexing was followed by sequencing analysis using a HiSeq 2000 sequencer (Illumina, http://www.illumina.com/).

### Small RNA sequencing data analysis

Small RNA sequencing data analysis was done as described (Carbonell *et al*., 2014). Custom scripts to process small RNA data sets are available at https://github.com/carringtonlab/srtools. Small RNA sequencing libraries from transgenic Arabidopsis inflorescences and Brachypodium calli or leaves, and from *N. benthamiana* agroinfiltrated leaves, are described in Table S9. *O. sativa* small RNA data sets used in the processing analysis of authentic *OsMIR390* presented in Figure 1(b) were described previously (Cuperus *et al*., 2010).

### Preparation of strand‐specific transcript libraries

Ten microgram of total RNA extracted from four independent lines per construct were treated with TURBO DNase I (Life Technologies). Ribosomal RNAs were depleted from samples by Ribo‐Zero Magnetic Kit ‘Plant Leaf’ (Epicentre, http://www.epibio.com/) treatment. cDNA synthesis followed by strand‐specific transcript library preparation were made as described with minor modifications (Wang *et al*., 2011; Carbonell *et al*., 2012). These included the fragmentation with metal ions during 4 min at 95°C of Ribo‐Zero treated RNAs, and the use of 14 cycles in the linear PCR reaction. Y‐shape adaptors were generated by annealing DNA adaptors 1 and 2, and PE‐F oligonucleotide was combined with one indexed oligonucleotide (PE‐R‐N701 to PE‐R‐N710) in the linear PCR (Table S7). DNA amplicon analysis, quantification and sequencing were done as described (Carbonell *et al*., 2014).

### Transcriptome analysis

FASTQ files were de‐multiplexed with the parseFastq.pl perl script (https://github.com/carringtonlab/srtools). Sequencing reads from each de‐multiplexed transcript library were mapped to *B. distachyon* transcriptome (v2.1; Phytozome 10, http://phytozome.jgi.doe.gov/pz/portal.html) using Butter (Axtell, 2014) and allowing one mismatch. Differential gene expression analysis was done using DESeq2 (Love *et al*., 2014) with a false discovery rate of 1%. For each *35S:GUS* versus *35S:OsMIR390‐AtL* pairwise comparison, genes having no expression (0 gene counts) in at least five of the eight samples were removed from the analysis. Differential gene expression analysis results are shown in Data S1.

targetfinder v1.7 (https://github.com/carringtonlab/TargetFinder) (Fahlgren and Carrington, 2010) was used to obtain a ranked list of potential off‐targets for each amiRNA. RNA‐Seq libraries from transgenic Brachypodium leaves are described in Table S10.

## Accession Numbers

*A. thaliana* genes and corresponding locus identifiers are: *AtACT2* (AT3G18780), *AtCBP20* (AT5G44200), *AtCH42* (AT4G18480), *AtCPC* (AT2G46410), *AtETC2* (AT2G30420), *AtFT* (AT1G65480), *AtSAND* (AT2G28390), *AtTRY* (AT5G53200) and *AtUBQ10* (AT4G05320). *B. distachyon* genes and corresponding locus identifiers are: *BdBRI1* (Bradi2g48280), *BdCAD1* (Bradi3g06480), *BdCAO* (Bradi2g61500), *BdSAMDC* (Bradi5g14640), *BdSPL11* (Bradi4g04270), *BdUBC18* (Bradi4g00660), *BdUBI4* (Bradi3g04730) and *BdUBI10* (Bradi1g32860). The miRBase (http://mirbase.org) (Kozomara and Griffiths‐Jones, 2014) locus identifiers of the conserved rice *MIRNA* precursors and plant *MIR390* precursors (Figure 1b) are detailed in Tables S1 and S2, respectively. High‐throughput sequencing data were deposited in the Sequence Read Archive (http://www.ncbi.nlm.nih.gov/sra) under accession number SRP052754.

## Supporting information


**Figure S1.** *OsMIR390‐B/c* vectors for direct cloning of amiRNAs.

**Figure S2.** Generation of constructs to express amiRNAs from authentic *OsMIR390* precursors.

**Figure S3.** Generation of constructs to express amiRNAs from chimeric *OsMIR390‐AtL* precursors.

**Figure S4.** Generation of constructs to express amiRNAs from chimeric *AtMIR390a‐OsL* precursors.

**Figure S5**. Base‐pairing of amiRNAs and Brachypodium target mRNAs.

**Figure S6**. Plant height and seed length analyses in Brachypodium T0 transgenic plants expressing amiR‐BdBri1 from authentic *OsMIR390* or chimeric *OsMIR390‐AtL* precursors.

**Figure S7**. Quantification of amiR‐BdCao‐induced phenotype in Brachypodium *35S:OsMIR390‐AtL‐Cao*,* 35S:OsMIR390‐Cao* and *35S:GUS* T0 transgenic lines.

**Figure S8**. Comparative analyses of the accumulation and processing of several amiRNAs derived from *AtMIR390a*,* AtMIR390a‐OsL*,* OsMIR390* and *OsMIR390‐AtL* precursors in *Nicotiana benthamiana* leaves.

**Figure S9.** Base‐pairing of amiRNAs and Arabidopsis target transcripts.

**Figure S10.** Functionality in Arabidopsis T1 transgenic plants of amiRNAs derived from *AtMIR390a*‐based chimeric precursors containing *Oryza sativa* distal stem‐loop sequences (*AtMIR390a‐OsL*).

**Figure S11.** AmiRNA‐induced phenotype quantification in Arabidopsis transgenic plants expressing amiR‐AtFt (left) and amiR‐AtCh42 (right) from *AtMIR390a* or chimeric *AtMIR390a‐OsL* precursors.

**Figure S12**. Target accumulation determined by RNA‐Seq analysis in transgenic Brachypodium plants including *35S:OsMIR390‐AtL*‐based or *35S:GUS* constructs.

**Table S1.** MiRbase locus identifiers of *Orzya sativa* conserved *MIRNA* precursors.

**Table S2.** MiRbase locus identifiers of plant *MIR390* precursors.

**Table S3.** AmiRNA phenotypic penetrance in Brachypodium T0 transgenic plants.

**Table S4.** AmiRNA phenotypic penetrance in Brachypodium T1 transgenic plants.

**Table S5.** AmiRNA phenotypic penetrance in Arabidopsis T1 transgenic plants.

**Table S6.** AmiRNA phenotypic penetrance in Arabidopsis T2 transgenic plants.

**Table S7.** DNA, LNA and RNA oligonucleotides.

**Table S8.** Sequences and predicted targets for all amiRNAs analyzed.

**Table S9.** High‐throughput small RNA libraries from Arabidopsis, Brachypodium or *Nicotiana benthamiana* plants.

**Table S10.** High‐throughput strand‐specific transcript RNA libraries from independent Brachypodium T0 transgenic lines.

**Data S1A.** Differential gene expression analysis between *35S:GUS* and *35S:OsMIR390‐AtL‐Bri1* Brachypodium samples.
**Data S1B.** Differential gene expression analysis between *35S:GUS* and *35S:OsMIR390‐AtL‐Cad1* Brachypodium samples.
**Data S1C.** Differential gene expression analysis between *35S:GUS* and *35S:OsMIR390‐AtL‐Cao* Brachypodium samples.
**Data S1D.** Differential gene expression analysis between *35S:GUS* and *35S:OsMIR390‐AtL‐Spl11* Brachypodium samples.

**Data S2.** Gene counts in RNA‐Seq libraries from *35S:GUS*,* 35S:0sMIR390‐AtL‐Bri1*,* 35S:OsMIR390‐AtL‐Cad1*,* 35S:0sMIR390‐AtL‐Cao* and *35S:OsMIR390‐AtL‐Spl11* transgenic Brachypodium lines.

**Data S3A.** amiR‐BdBri1 predicted off‐targets differentially underexpressed in *35S:OsMIR390‐AtL‐Bri1* transgenic Brachypodium plants.
**Data S3B.** amiR‐BdCad1 predicted off‐targets differentially underexpressed in *35S:OsMIR390‐AtL‐Cad1* transgenic Brachypodium plants.
**Data S3C.** amiR‐BdCao predicted off‐targets differentially underexpressed in *35S:OsMIR390‐AtL‐Cao* transgenic Brachypodium plants.
**Data S3D.** amiR‐BdSpl11 predicted off‐targets differentially underexpressed in *35S:OsMIR390‐AtL‐Spl11* transgenic Brachypodium plants.

**Appendix S1.** Characterization of *AtMIR390a‐OsL*‐based amiRNAs in eudicots.

**Appendix S2**. DNA sequence of B/c vectors used for direct cloning of amiRNAs in zero‐background vectors containing the *OsMIR390* sequence.

**Appendix S3.** FASTA sequences of all amiRNA‐producing *MIRNA* precursors analyzed.

**Appendix S4.** Protocol to clone amiRNAs in *Bsa*I/*ccd*B‐based (‘B/c’) vectors including the *OsMIR390* precursor.

## References

[tpj12835-bib-0001] Addo‐Quaye, C. , Eshoo, T.W. , Bartel, D.P. and Axtell, M.J. (2008) Endogenous siRNA and miRNA targets identified by sequencing of the Arabidopsis degradome. Curr. Biol. 18, 758–762. 18472421 10.1016/j.cub.2008.04.042 PMC2583427

[tpj12835-bib-0002] Alvarez, J.P. , Pekker, I. , Goldshmidt, A. , Blum, E. , Amsellem, Z. and Eshed, Y. (2006) Endogenous and synthetic microRNAs stimulate simultaneous, efficient, and localized regulation of multiple targets in diverse species. Plant Cell, 18, 1134–1151. 16603651 10.1105/tpc.105.040725 PMC1456869

[tpj12835-bib-0003] Arikit, S. , Zhai, J. and Meyers, B.C. (2013) Biogenesis and function of rice small RNAs from non‐coding RNA precursors. Curr. Opin. Plant Biol. 16, 170–179. 23466255 10.1016/j.pbi.2013.01.006

[tpj12835-bib-0004] Axtell, M.J. (2013) Classification and comparison of small RNAs from plants. Annu. Rev. Plant Biol. 64, 137–159. 23330790 10.1146/annurev-arplant-050312-120043

[tpj12835-bib-0005] Axtell, M.J. (2014) Butter: high‐precision genomic alignment of small RNA‐Seq data. bioRxiv, doi.org/10.1101/007427.

[tpj12835-bib-0006] Axtell, M.J. , Jan, C. , Rajagopalan, R. and Bartel, D.P. (2006) A two‐hit trigger for siRNA biogenesis in plants. Cell, 127, 565–577. 17081978 10.1016/j.cell.2006.09.032

[tpj12835-bib-0007] Bartel, D.P. (2004) MicroRNAs: genomics, biogenesis, mechanism, and function. Cell, 116, 281–297. 14744438 10.1016/s0092-8674(04)00045-5

[tpj12835-bib-0008] Bernard, P. and Couturier, M. (1992) Cell killing by the F plasmid CcdB protein involves poisoning of DNA‐topoisomerase II complexes. J. Mol. Biol. 226, 735–745. 1324324 10.1016/0022-2836(92)90629-x

[tpj12835-bib-0009] Bologna, N.G. and Voinnet, O. (2014) The diversity, biogenesis, and activities of endogenous silencing small RNAs in Arabidopsis. Annu. Rev. Plant Biol. 65, 473–503. 24579988 10.1146/annurev-arplant-050213-035728

[tpj12835-bib-0010] Bouvier d'Yvoire, M. , Bouchabke‐Coussa, O. , Voorend, W. ***et al.*** (2013) Disrupting the cinnamyl alcohol dehydrogenase 1 gene (BdCAD1) leads to altered lignification and improved saccharification in *Brachypodium distachyon* . Plant J. 73, 496–508. 23078216 10.1111/tpj.12053

[tpj12835-bib-0011] Butardo, V.M. , Fitzgerald, M.A. , Bird, A.R. ***et al.*** (2011) Impact of down‐regulation of starch branching enzyme IIb in rice by artificial microRNA‐ and hairpin RNA‐mediated RNA silencing. J. Exp. Bot. 62, 4927–4941. 21791436 10.1093/jxb/err188 PMC3193005

[tpj12835-bib-0012] Carbonell, A. , Fahlgren, N. , Garcia‐Ruiz, H. , Gilbert, K.B. , Montgomery, T.A. , Nguyen, T. , Cuperus, J.T. and Carrington, J.C. (2012) Functional analysis of three Arabidopsis ARGONAUTES using slicer‐defective mutants. Plant Cell, 24, 3613–3629. 23023169 10.1105/tpc.112.099945 PMC3480291

[tpj12835-bib-0013] Carbonell, A. , Takeda, A. , Fahlgren, N. , Johnson, S.C. , Cuperus, J.T. and Carrington, J.C. (2014) New generation of artificial microRNA and synthetic trans‐acting small interfering RNA vectors for efficient gene silencing in Arabidopsis. Plant Physiol. 165, 15–29. 24647477 10.1104/pp.113.234989 PMC4012576

[tpj12835-bib-0014] Chen, H. , Jiang, S. , Zheng, J. and Lin, Y. (2012a) Improving panicle exsertion of rice cytoplasmic male sterile line by combination of artificial microRNA and artificial target mimic. Plant Biotechnol. J. 11, 336–343. 23164055 10.1111/pbi.12019

[tpj12835-bib-0015] Chen, M. , Wei, X. , Shao, G. , Tang, S. , Luo, J. and Hu, P. (2012b) Fragrance of the rice grain achieved via artificial microRNA‐induced down‐regulation ofOsBADH2. Plant Breed. 131, 584–590.

[tpj12835-bib-0017] Cuperus, J.T. , Carbonell, A. , Fahlgren, N. , Garcia‐Ruiz, H. , Burke, R.T. , Takeda, A. , Sullivan, C.M. , Gilbert, S.D. , Montgomery, T.A. and Carrington, J.C. (2010) Unique functionality of 22‐nt miRNAs in triggering RDR6‐dependent siRNA biogenesis from target transcripts in Arabidopsis. Nat. Struct. Mol. Biol. 17, 997–1003. 20562854 10.1038/nsmb.1866 PMC2916640

[tpj12835-bib-0018] Cuperus, J.T. , Fahlgren, N. and Carrington, J.C. (2011) Evolution and functional diversification of MIRNA genes. Plant Cell, 23, 431–442. 21317375 10.1105/tpc.110.082784 PMC3077775

[tpj12835-bib-0019] Endo, Y. , Iwakawa, H.O. and Tomari, Y. (2013) Arabidopsis ARGONAUTE7 selects miR390 through multiple checkpoints during RISC assembly. EMBO Rep. 14, 652–658. 23732541 10.1038/embor.2013.73 PMC3701240

[tpj12835-bib-0020] Fahlgren, N. and Carrington, J.C. (2010) miRNA target prediction in plants. Methods Mol. Biol. 592, 51–57. 19802588 10.1007/978-1-60327-005-2_4

[tpj12835-bib-0021] Felippes, F.F. and Weigel, D. (2009) Triggering the formation of tasiRNAs in *Arabidopsis thaliana*: the role of microRNA miR173. EMBO Rep. 10, 264–270. 19180117 10.1038/embor.2008.247 PMC2658565

[tpj12835-bib-0022] Gilbert, K.B. , Fahlgren, N. , Kasschau, K.D. , Chapman, E.J. , Carrington, J.C. and Carbonell, A. (2014) Preparation of multiplexed small RNA libraries from plants. Bio Protoc. 4, e1275. 10.21769/bioprotoc.1275 PMC4675356 26661568

[tpj12835-bib-0023] Guo, Y. , Han, Y. , Ma, J. , Wang, H. , Sang, X. and Li, M. (2014) Undesired small RNAs originate from an artificial microRNA precursor in transgenic petunia (*Petunia hybrida*). PLoS ONE, 9, e98783. 24897430 10.1371/journal.pone.0098783 PMC4045805

[tpj12835-bib-0024] He, G. , Zhu, X. , Elling, A.A. ***et al.*** (2010) Global epigenetic and transcriptional trends among two rice subspecies and their reciprocal hybrids. Plant Cell, 22, 17–33. 20086188 10.1105/tpc.109.072041 PMC2828707

[tpj12835-bib-0025] Heisel, S.E. , Zhang, Y. , Allen, E. , Guo, L. , Reynolds, T.L. , Yang, X. , Kovalic, D. and Roberts, J.K. (2008) Characterization of unique small RNA populations from rice grain. PLoS ONE, 3, e2871. 18716673 10.1371/journal.pone.0002871 PMC2518513

[tpj12835-bib-0026] Johnson, C. , Kasprzewska, A. , Tennessen, K. , Fernandes, J. , Nan, G.L. , Walbot, V. , Sundaresan, V. , Vance, V. and Bowman, L.H. (2009) Clusters and superclusters of phased small RNAs in the developing inflorescence of rice. Genome Res. 19, 1429–1440. 19584097 10.1101/gr.089854.108 PMC2720183

[tpj12835-bib-0200] Karimi, M. , Inze, D. and Depicker, A. (2002) GATEWAY vectors for Agrobacterium‐mediated plant transformation. Trends Plant Science, 7, 193–195. 10.1016/s1360-1385(02)02251-3 11992820

[tpj12835-bib-0027] Kozomara, A. and Griffiths‐Jones, S. (2014) miRBase: annotating high confidence microRNAs using deep sequencing data. Nucleic Acids Res. 42, D68–D73. 24275495 10.1093/nar/gkt1181 PMC3965103

[tpj12835-bib-0028] Liang, G. , He, H. , Li, Y. and Yu, D. (2012) A new strategy for construction of artificial miRNA vectors in Arabidopsis. Planta, 235, 1421–1429. 22350768 10.1007/s00425-012-1610-5

[tpj12835-bib-0029] Liu, Q. , Wang, F. and Axtell, M.J. (2014) Analysis of complementarity requirements for plant microRNA targeting using a *Nicotiana benthamiana* quantitative transient assay. Plant Cell, 26, 741–753. 24510721 10.1105/tpc.113.120972 PMC3967037

[tpj12835-bib-0030] Love, M.I. , Huber, W. and Anders, S. (2014) Moderated estimation of fold change and dispersion for RNA‐Seq data with DESeq2. bioRxiv, doi.org/10.1101/002832. 10.1186/s13059-014-0550-8 PMC4302049 25516281

[tpj12835-bib-0031] Mi, S. , Cai, T. , Hu, Y. ***et al.*** (2008) Sorting of small RNAs into Arabidopsis argonaute complexes is directed by the 5′ terminal nucleotide. Cell, 133, 116–127. 18342361 10.1016/j.cell.2008.02.034 PMC2981139

[tpj12835-bib-0032] Montgomery, T.A. , Howell, M.D. , Cuperus, J.T. , Li, D. , Hansen, J.E. , Alexander, A.L. , Chapman, E.J. , Fahlgren, N. , Allen, E. and Carrington, J.C. (2008) Specificity of ARGONAUTE7‐miR390 interaction and dual functionality in TAS3 trans‐acting siRNA formation. Cell, 133, 128–141. 18342362 10.1016/j.cell.2008.02.033

[tpj12835-bib-0033] Ossowski, S. , Schwab, R. and Weigel, D. (2008) Gene silencing in plants using artificial microRNAs and other small RNAs. Plant J. 53, 674–690. 18269576 10.1111/j.1365-313X.2007.03328.x

[tpj12835-bib-0034] Oster, U. , Tanaka, R. , Tanaka, A. and Rudiger, W. (2000) Cloning and functional expression of the gene encoding the key enzyme for chlorophyll b biosynthesis (CAO) from *Arabidopsis thaliana* . Plant J. 21, 305–310. 10758481 10.1046/j.1365-313x.2000.00672.x

[tpj12835-bib-0035] Philippar, K. , Geis, T. , Ilkavets, I. , Oster, U. , Schwenkert, S. , Meurer, J. and Soll, J. (2007) Chloroplast biogenesis: the use of mutants to study the etioplast‐chloroplast transition. Proc. Natl Acad. Sci. USA, 104, 678–683. 17202255 10.1073/pnas.0610062104 PMC1766443

[tpj12835-bib-0036] Rapaport, F. , Khanin, R. , Liang, Y. , Pirun, M. , Krek, A. , Zumbo, P. , Mason, C.E. , Socci, N.D. and Betel, D. (2013) Comprehensive evaluation of differential gene expression analysis methods for RNA‐seq data. Genome Biol. 14, R95. 24020486 10.1186/gb-2013-14-9-r95 PMC4054597

[tpj12835-bib-0037] Schwab, R. , Ossowski, S. , Riester, M. , Warthmann, N. and Weigel, D. (2006) Highly specific gene silencing by artificial microRNAs in Arabidopsis. Plant Cell, 18, 1121–1133. 16531494 10.1105/tpc.105.039834 PMC1456875

[tpj12835-bib-0038] Takeda, A. , Iwasaki, S. , Watanabe, T. , Utsumi, M. and Watanabe, Y. (2008) The mechanism selecting the guide strand from small RNA duplexes is different among argonaute proteins. Plant Cell Physiol. 49, 493–500. 18344228 10.1093/pcp/pcn043

[tpj12835-bib-0039] Tanaka, A. , Ito, H. , Tanaka, R. , Tanaka, N.K. , Yoshida, K. and Okada, K. (1998) Chlorophyll *a* oxygenase (CAO) is involved in chlorophyll *b* formation from chlorophyll *a* . Proc. Natl Acad. Sci. USA, 95, 12719–12723. 9770552 10.1073/pnas.95.21.12719 PMC22897

[tpj12835-bib-0040] Thole, V. , Peraldi, A. , Worland, B. , Nicholson, P. , Doonan, J.H. and Vain, P. (2012) T‐DNA mutagenesis in *Brachypodium distachyon* . J. Exp. Bot. 63, 567–576. 22090444 10.1093/jxb/err333

[tpj12835-bib-0041] Tiwari, M. , Sharma, D. and Trivedi, P.K. (2014) Artificial microRNA mediated gene silencing in plants: progress and perspectives. Plant Mol. Biol. 86, 1–18. 25022825 10.1007/s11103-014-0224-7

[tpj12835-bib-0042] Trabucco, G.M. , Matos, D.A. , Lee, S.J. , Saathoff, A.J. , Priest, H.D. , Mockler, T.C. , Sarath, G. and Hazen, S.P. (2013) Functional characterization of cinnamyl alcohol dehydrogenase and caffeic acid *O*‐methyltransferase in *Brachypodium distachyon* . BMC Biotechnol. 13, 61. 23902793 10.1186/1472-6750-13-61 PMC3734214

[tpj12835-bib-0043] Vogel, J. and Hill, T. (2008) High‐efficiency *Agrobacterium*‐mediated transformation of *Brachypodium distachyon* inbred line Bd21‐3. Plant Cell Rep. 27, 471–478. 17999063 10.1007/s00299-007-0472-y

[tpj12835-bib-0044] Wang, L. , Si, Y. , Dedow, L.K. , Shao, Y. , Liu, P. and Brutnell, T.P. (2011) A low‐cost library construction protocol and data analysis pipeline for Illumina‐based strand‐specific multiplex RNA‐seq. PLoS ONE, 6, e26426. 22039485 10.1371/journal.pone.0026426 PMC3198403

[tpj12835-bib-0045] Warthmann, N. , Chen, H. , Ossowski, S. , Weigel, D. and Herve, P. (2008) Highly specific gene silencing by artificial miRNAs in rice. PLoS ONE, 3, e1829. 18350165 10.1371/journal.pone.0001829 PMC2262943

[tpj12835-bib-0046] Zeng, L.R. , Qu, S. , Bordeos, A. , Yang, C. , Baraoidan, M. , Yan, H. , Xie, Q. , Nahm, B.H. , Leung, H. and Wang, G.L. (2004) Spotted leaf11, a negative regulator of plant cell death and defense, encodes a U‐box/armadillo repeat protein endowed with E3 ubiquitin ligase activity. Plant Cell, 16, 2795–2808. 15377756 10.1105/tpc.104.025171 PMC520972

[tpj12835-bib-0047] Zhang, X. , Niu, D. , Carbonell, A. , Wang, A. , Lee, A. , Tun, V. , Wang, Z. , Carrington, J.C. , Chang, C.E. and Jin, H. (2014) ARGONAUTE PIWI domain and microRNA duplex structure regulate small RNA sorting in Arabidopsis. Nat. Commun. 5, 5468. 25406978 10.1038/ncomms6468 PMC4238042

[tpj12835-bib-0048] Zhou, X. , Sunkar, R. , Jin, H. , Zhu, J.K. and Zhang, W. (2009) Genome‐wide identification and analysis of small RNAs originated from natural antisense transcripts in *Oryza sativa* . Genome Res. 19, 70–78. 18971307 10.1101/gr.084806.108 PMC2612963

[tpj12835-bib-0049] Zhu, Q.H. , Spriggs, A. , Matthew, L. , Fan, L. , Kennedy, G. , Gubler, F. and Helliwell, C. (2008) A diverse set of microRNAs and microRNA‐like small RNAs in developing rice grains. Genome Res. 18, 1456–1465. 18687877 10.1101/gr.075572.107 PMC2527712

[tpj12835-bib-0050] Zhu, H. , Hu, F. , Wang, R. , Zhou, X. , Sze, S.H. , Liou, L.W. , Barefoot, A. , Dickman, M. and Zhang, X. (2011) Arabidopsis Argonaute10 specifically sequesters miR166/165 to regulate shoot apical meristem development. Cell, 145, 242–256. 21496644 10.1016/j.cell.2011.03.024 PMC4124879

[tpj12835-bib-0051] Zhu, J.Y. , Sae‐Seaw, J. and Wang, Z.Y. (2013) Brassinosteroid signalling. Development, 140, 1615–1620. 23533170 10.1242/dev.060590 PMC3621480

