# Supplementary material for: Highly specific gene silencing in a monocot species by artificial microRNAs derived from chimeric miRNA precursors
Source: Plant J. 2015 May 20;82(6):1061–75. doi: 10.1111/tpj.12835 (PMC4464980; doi:10.1111/tpj.12835)
Supplement: Supplementary file 1 — Figure S1. OsMIR390‐B/c vectors for direct cloning of amiRNAs. [file TPJ-82-1061-s001.pdf]

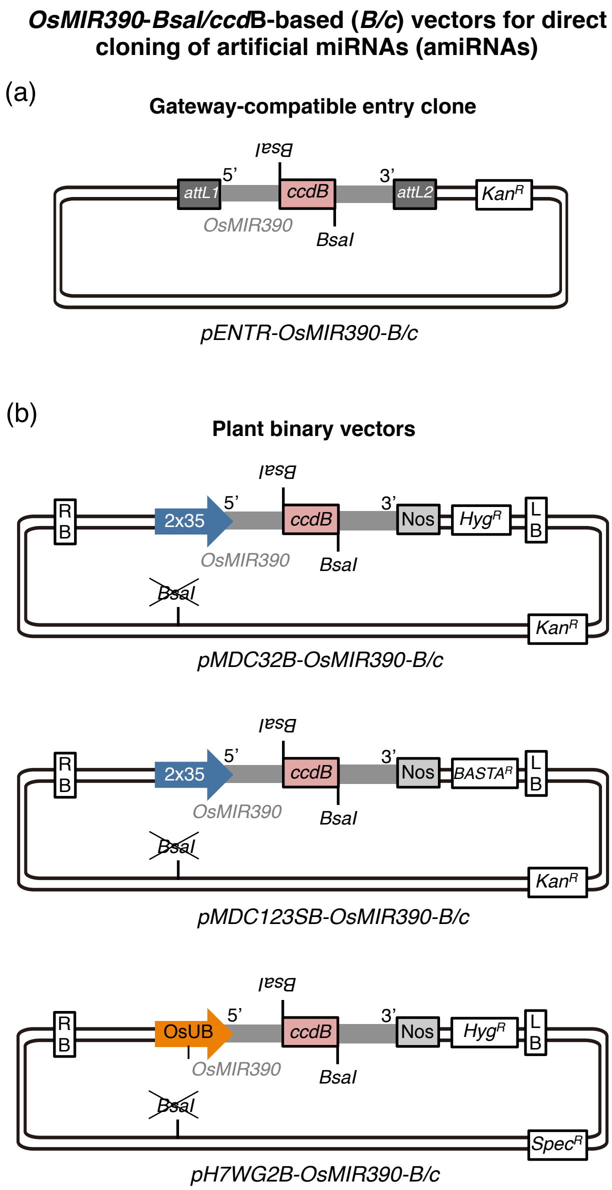

Figure S1. *OsMIR390-B/c* vectors for direct cloning of amiRNAs.
(a) Diagram of an *OsMIR390-B/c* Gateway-compatible entry vector (*pENTR-OsMIR390-B/c)*.
(b) Diagrams of *OsMIR390-B/c*-based binary vectors for expression of amiRNAs in monocot species (*pMDC32B-OsMIR390-B/c*, *pMDC123SB-OsMIR390-B/c* and *pH7WG2B-OsMIR390-B/c*). RB: right border; 35S: *Cauliflower mosaic virus* promoter; OsUbi: *Oryza sativa* ubiquitin 2 promoter; *Bsa*I: *Bsa*I recognition site, *ccd*B: gene encoding the *ccd*B toxin; LB: left border; attL1 and attL2: gateway recombination sites. *Kan^R*: kanamycin resistance gene; *Hyg^R*: hygromycin resistance gene; *Basta^R*: glufosinate resistance gene; *Spec^R*: spectinomycin resistance gene. Undesired *Bsa*I sites removed from the plasmid are crossed out.
