# Supplementary material for: Highly specific gene silencing in a monocot species by artificial microRNAs derived from chimeric miRNA precursors
Source: Plant J. 2015 May 20;82(6):1061–75. doi: 10.1111/tpj.12835 (PMC4464980; doi:10.1111/tpj.12835)
Supplement: Supplementary file 3 — Figure S3. Generation of constructs to express amiRNAs from chimeric OsMIR390‐AtL precursors. [file TPJ-82-1061-s003.pdf]

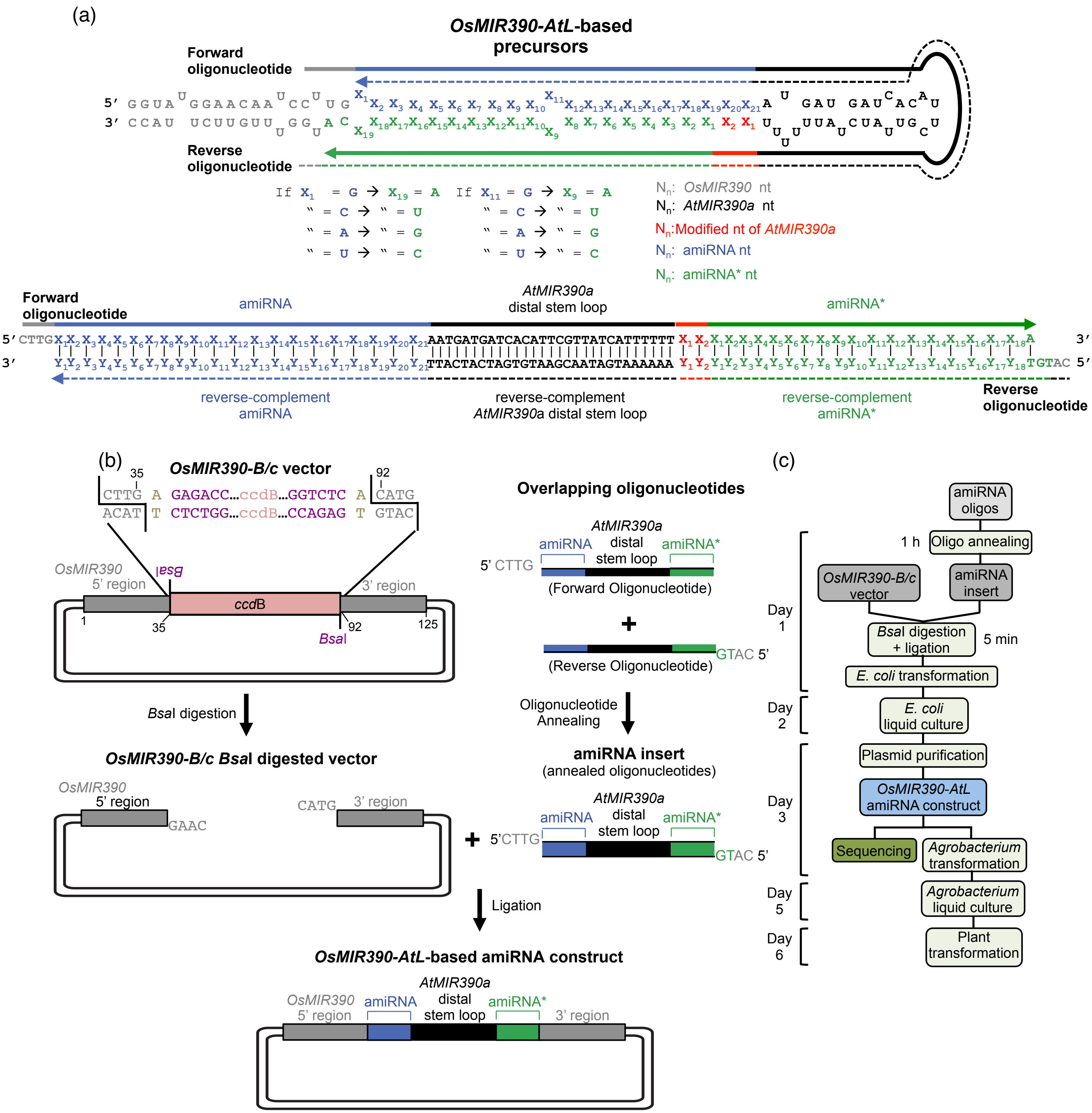

Figure S3. Generation of constructs to express amiRNAs from chimeric *OsMIR390-AtL* precursors.
(a) Design of the two overlapping oligonucleotides containing *OsMIR390aa* and *AtMIR390a* basal stem and distal stem loop sequences, respectively. Sequences covered by the forward and reverse oligonucleotides are represented with solid and dotted lines, respectively. Nucleotides of *AtMIR390a* and *OsMIR390* precursors are in black and grey, respectively. Nucleotides of the amiRNA guide strand, and amiRNA* strand are in blue, and green respectively. Other *OsMIR390* nucleotides that may be modified for preserving authentic *OsMIR390* precursor secondary structure are in red. Rules for assigning identity to positions 1 and 9 of amiRNA* are indicated.

Figure S3 (Cont.) (b) Diagram of the steps for generating constructs for expressing amiRNAs from chimeric *OsMIR390-AtL* precursors. The amiRNA insert obtained after annealing the two overlapping oligonucleotides has 5'CTTG and 5'CATG overhangs and is directly inserted in a directional manner into an *OsMIR390-B/c* vector previously linearized with *Bsa*I. Nucleotides of the *Bsa*I sites and those arbitrarily chosen and used as spacers between the *Bsa*I recognition sites and the *OsMIR390* sequence are in purple and light brown, respectively. Other details are as described in (a).

(c) Flow chart of the steps from amiRNA construct generation to plant transformation.
