# Supplementary material for: Highly specific gene silencing in a monocot species by artificial microRNAs derived from chimeric miRNA precursors
Source: Plant J. 2015 May 20;82(6):1061–75. doi: 10.1111/tpj.12835 (PMC4464980; doi:10.1111/tpj.12835)
Supplement: Supplementary file 5 — Figure S5. Base‐pairing of amiRNAs and Brachypodium target mRNAs. [file TPJ-82-1061-s005.pdf]

```
amiR-BdBri1     5'  TCGCAATCTTCCGCCTTGCTC  3'
                    ||||||||||||||||||||
BdBRI1
(Bradi2g48280)  3'  AGCGTTAGAAGGCGGAACGAC  5'

amiR-BdCad1     5'  TCGATCTGAGAAGTAAGCCCA  3'
                    ||||||||||||||||||||
BdCAD1
(Bradi3g06480)  3'  AGCTAGACTCTTCATTCGGGA  5'

amiR-BdCao      5'  TCTGCATGGATTGTAAACCCA  3'
                    ||||||||||||||||||||
BdCAO
(Bradi2g61500)  3'  AGACGTACCTAACATTTGGGA  5'

amiR-BdSpl11    5'  TTAGCAACACTACAAGGGCAC  3'
                    ||||||||||||||||||||
BdSPL11
(Bradi4g04270)  3'  AATCGTTGTGATGTTCCCGTC  5'
```

Figure S5. Base-pairing of amiRNAs and Brachypodium target mRNAs. amiRNA and mRNA target nucleotides are in blue and brown, respectively.
