# Supplementary material for: Highly specific gene silencing in a monocot species by artificial microRNAs derived from chimeric miRNA precursors
Source: Plant J. 2015 May 20;82(6):1061–75. doi: 10.1111/tpj.12835 (PMC4464980; doi:10.1111/tpj.12835)
Supplement: Supplementary file 6 — Figure S6. Plant height and seed length analyses in Brachypodium T0 transgenic plants expressing amiR‐BdBri1 from authentic OsMIR390 or chimeric OsMIR390‐AtL precursors. [file TPJ-82-1061-s006.pdf]

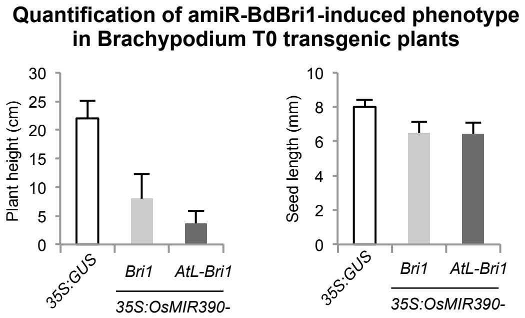

Figure S6. Plant height and seed length analyses in *Brachypodium distachyon* T0 transgenic plants expressing amiR-BdBri1 from authentic *OsMIR390* or chimeric *OsMIR390-AtL* precursors.
