# Supplementary material for: Highly specific gene silencing in a monocot species by artificial microRNAs derived from chimeric miRNA precursors
Source: Plant J. 2015 May 20;82(6):1061–75. doi: 10.1111/tpj.12835 (PMC4464980; doi:10.1111/tpj.12835)
Supplement: Supplementary file 7 — Figure S7. Quantification of amiR‐BdCao‐induced phenotype in Brachypodium 35S:OsMIR390‐AtL‐Cao, 35S:OsMIR390‐Cao and 35S:GUS T0 transgenic lines. [file TPJ-82-1061-s007.pdf]

(a)

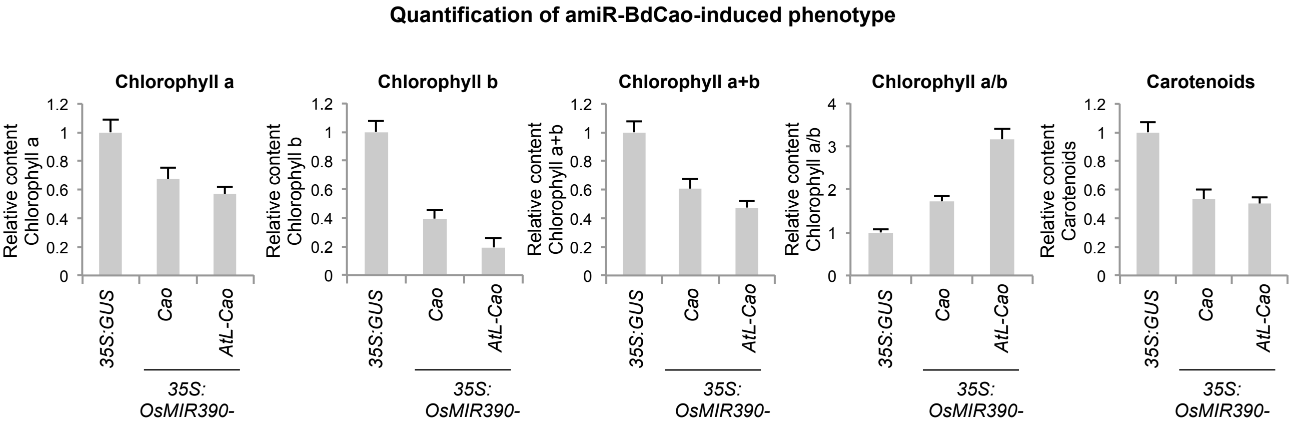

(b)

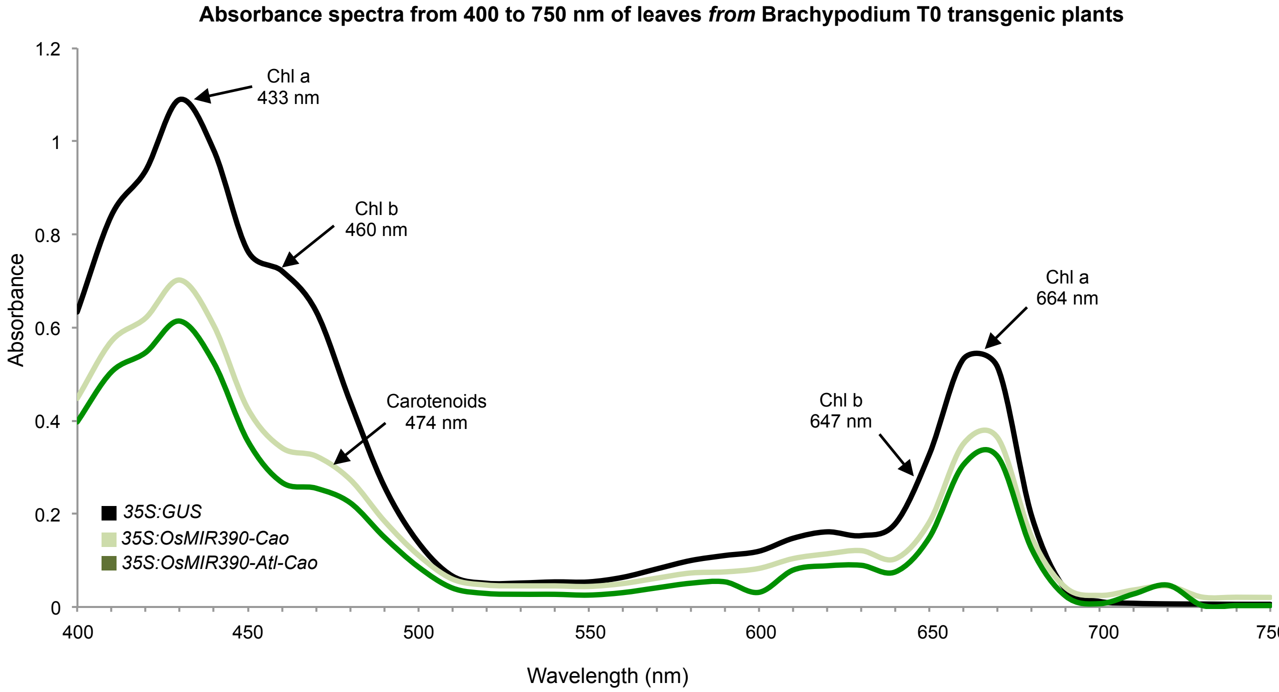

Figure S7. Quantification of amiR-BdCao-induced phenotype in *Brachypodium distachyon 35S:OsMIR390-AtL-Cao*, *35S:OsMIR390-Cao* and *35S:GUS* T0 transgenic lines.

(a) Quantification of chlorophyll a, chlorophyll b, chlorophyll a+b, chlorophyll a/b, and carotenoid content.

(b) Absorbance spectra from 400 to 750 nm of leaves from Brachypodium transgenic lines. Arrows indicate absorbance wavelengths of chlorophyll a (Chl a), chlorophyll b (Chl b), and carotenoids.
