# Supplementary material for: Highly specific gene silencing in a monocot species by artificial microRNAs derived from chimeric miRNA precursors
Source: Plant J. 2015 May 20;82(6):1061–75. doi: 10.1111/tpj.12835 (PMC4464980; doi:10.1111/tpj.12835)
Supplement: Supplementary file 8 — Figure S8. Comparative analyses of the accumulation and processing of several amiRNAs derived from AtMIR390a, AtMIR390a‐OsL, OsMIR390 and OsMIR390‐AtL precursors in Nicotiana benthamiana leaves. [file TPJ-82-1061-s008.pdf]

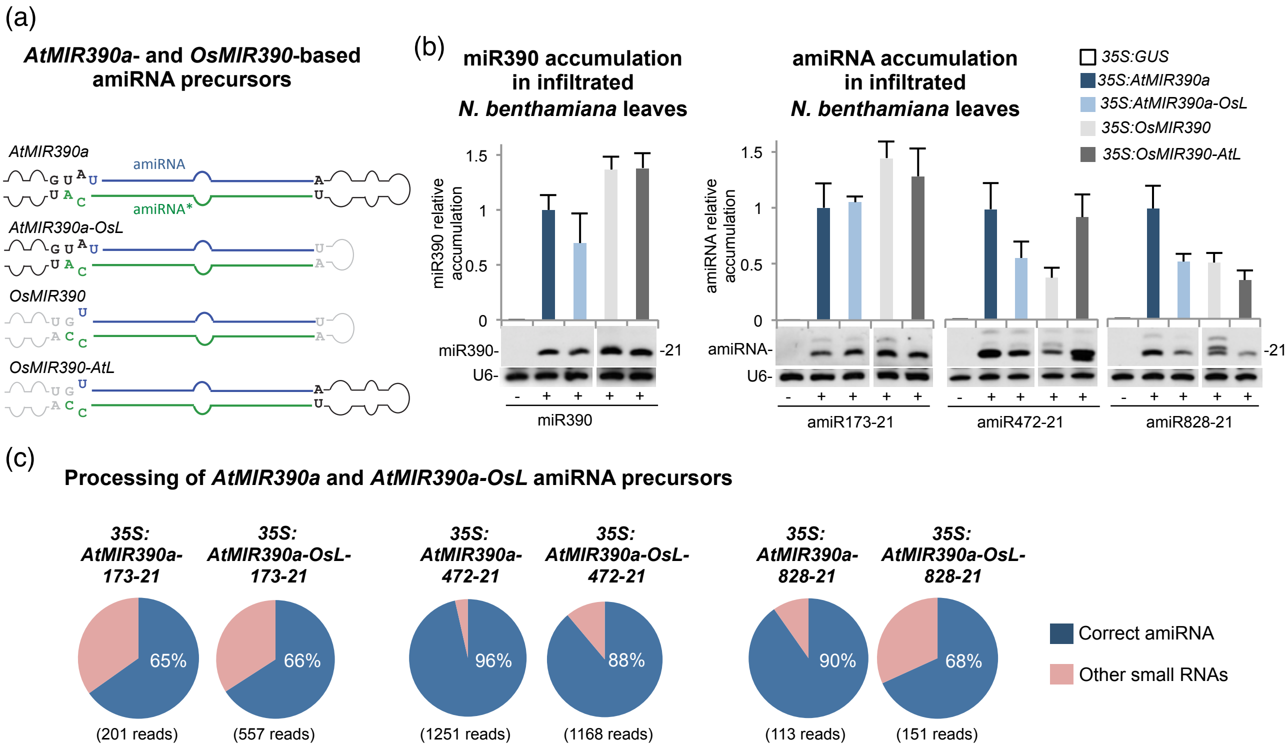

Figure S8. Comparative analysis of the accumulation and processing of several amiRNAs produced from *AtMIR390a*, *AtMIR390a-OsL*, *OsMIR390* and *OsMIR390-AtL* based precursors in *Nicotiana benthamiana* leaves.

(a) Diagrams of *AtMIR390a*, *AtMIR390a-OsL*, *OsMIR390* and *OsMIR390a-AtL* precursors. Nucleotides corresponding to the miRNA guide strand are in blue, and nucleotides of the miRNA* strand are in green. Other nucleotides from the *AtMIR390a* and *OsMIR390* precursors are in black and grey, respectively. Shapes of the *AtMIR390a* and *OsMIR390* precursors are in black and grey, respectively.

(b) Accumulation of miR390 (left) and of several 21-nucleotide amiRNAs (right) expressed from the *AtMIR390a*, *AtMIR390a-OsL*, *OsMIR390* or *OsMIR390-AtL* precursors in *N. benthamiana* leaves. Mean (n=3) relative amiRNA levels + s.d. when expressed from the *AtMIR390a* (dark blue, amiRNA level =1.0). Only one blot from three biological replicates is shown. U6 RNA blot is shown as loading control.

(c) Processing analysis of *AtMIR390a* and *AtMIR390-OsL* amiRNA precursors. Pie charts show the percentage of reads corresponding to accurately processed 21-nt mature amiRNAs (blue sectors) or to other small RNAs (pink sectors).
