# Supplementary material for: Highly specific gene silencing in a monocot species by artificial microRNAs derived from chimeric miRNA precursors
Source: Plant J. 2015 May 20;82(6):1061–75. doi: 10.1111/tpj.12835 (PMC4464980; doi:10.1111/tpj.12835)
Supplement: Supplementary file 9 — Figure S9. Base‐pairing of amiRNAs and Arabidopsis target transcripts. [file TPJ-82-1061-s009.pdf]

```
amiR-AtFt     5'  TTGGTTATAAAGGAAGAGGCC  3'
                  |||||||||||||||||:|||
target mRNA   3'  AACCAATATTTCCTTCTTCGG  5'
                          AtFT

amiR-AtCh42   5'  TTAAGTGTCACGGAAATCCCT  3'
                   |||||||||||||||||| |
target mRNA   3'  CATTCACAGTGCCTTTAGGAA  5'
                          AtCH42

amiR-AtTrich  5'  TCCCATTCGATACTGCTCGCC  3'
                  ||||||||||||||||||:|
target mRNA   3'  AGGGTAAGCTATGACGAGTGA  5'
                          AtTRY

              5'  TCCCATTCGATACTGCTCGCC  3'
                  ||||||||||||||:|||:||
              3'  AGGGTAAGCTATGATGAGTGG  5'
                          AtCPC

              5'  TCCCATTCGATACTGCTCGCC  3'
                  ||||||||||| ||:|||:||
              3'  AGGGTAAGCTACGATGAGTGA  5'
                          AtETC2
```

Figure S9. Base-pairing of amiRNAs and Arabidopsis target mRNAs. amiRNA and mRNA target nucleotides are in blue and brown, respectively.
