# Supplementary material for: Highly specific gene silencing in a monocot species by artificial microRNAs derived from chimeric miRNA precursors
Source: Plant J. 2015 May 20;82(6):1061–75. doi: 10.1111/tpj.12835 (PMC4464980; doi:10.1111/tpj.12835)
Supplement: Supplementary file 10 — Figure S10. Functionality in Arabidopsis T1 transgenic plants of amiRNAs derived from AtMIR390a‐based chimeric precursors containing Oryza sativa distal stem‐loop sequences (AtMIR390a‐OsL). [file TPJ-82-1061-s010.pdf]

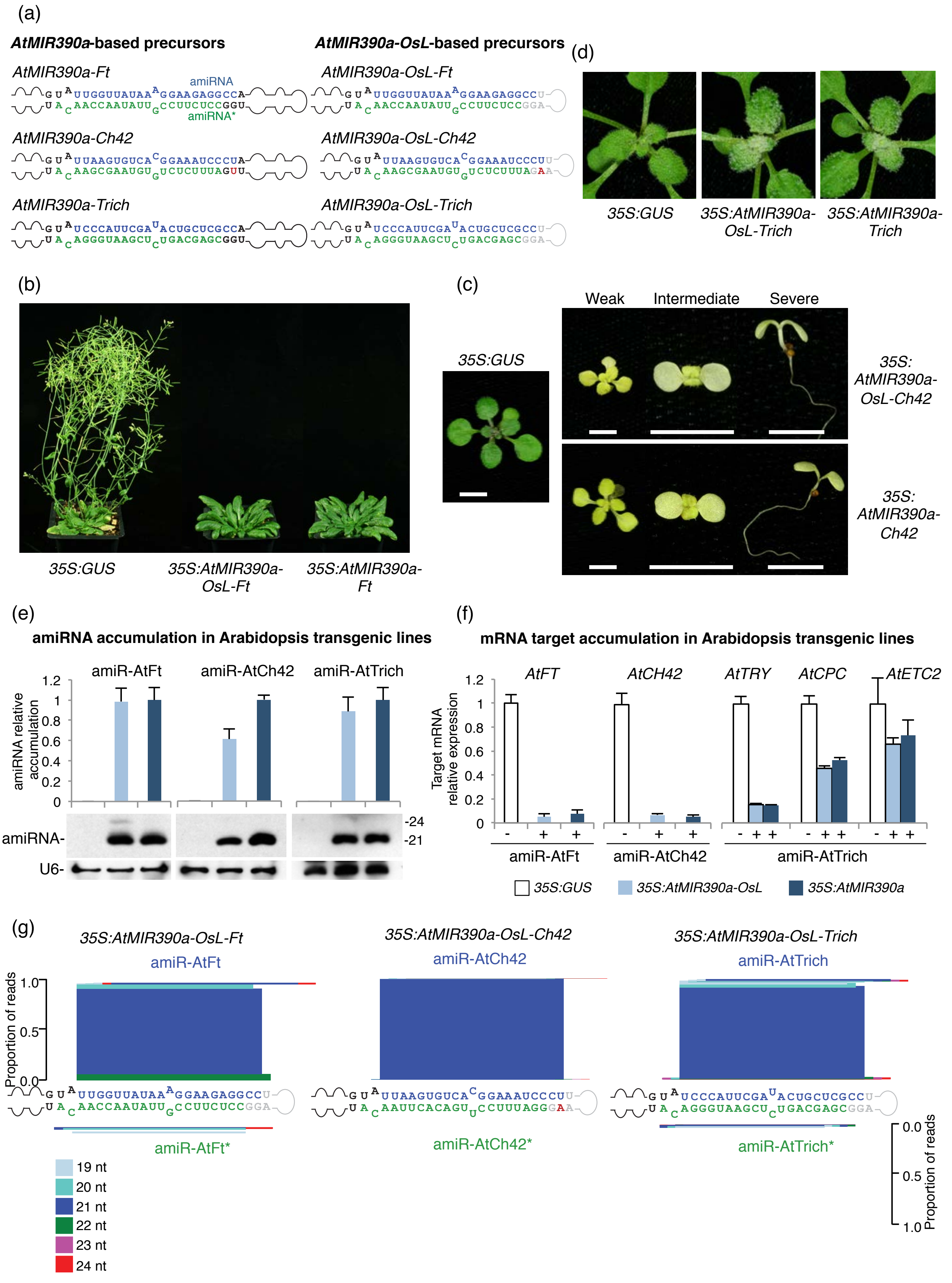

(a)
AtMIR390a-based precursors
AtMIR390a-OsL-based precursors
AtMIR390a-Ft
amiRNA
amiRNA*
AtMIR390a-Ch42
AtMIR390a-Trich
AtMIR390a-OsL-Ft
AtMIR390a-OsL-Ch42
AtMIR390a-OsL-Trich
(d)
35S:GUS
35S:AtMIR390a-OsL-Trich
35S:AtMIR390a-Trich
(b)
35S:GUS
35S:AtMIR390a-OsL-Ft
35S:AtMIR390a-Ft
(c)
Weak
Intermediate
Severe
35S:GUS
35S:AtMIR390a-OsL-Ch42
35S:AtMIR390a-Ch42
(e)
amiRNA accumulation in Arabidopsis transgenic lines
amiR-AtFt
amiR-AtCh42
amiR-AtTrich
amiRNA relative accumulation
amiRNA-
U6-
-24
-21
(f)
mRNA target accumulation in Arabidopsis transgenic lines
AtFT
AtCH42
AtTRY
AtCPC
AtETC2
Target mRNA relative expression
amiR-AtFt
amiR-AtCh42
amiR-AtTrich
35S:GUS
35S:AtMIR390a-OsL
35S:AtMIR390a
(g)
35S:AtMIR390a-OsL-Ft
35S:AtMIR390a-OsL-Ch42
35S:AtMIR390a-OsL-Trich
amiR-AtFt
amiR-AtCh42
amiR-AtTrich
Proportion of reads
amiR-AtFt*
amiR-AtCh42*
amiR-AtTrich*
19 nt
20 nt
21 nt
22 nt
23 nt
24 nt

Figure S10. Functionality in Arabidopsis T1 transgenic plants of amiRNAs derived from *AtMIR390a*-based chimeric precursors containing *Oryza sativa* distal stem-loop sequences (*AtMIR390a-OsL*).

(a) *AtMIR390a*- and *AtMIR390a-OsL*-based precursors containing Ft-, Ch42- and Trich-amiRNAs. Nucleotides corresponding to the miRNA guide and miRNA* strands are in blue and green, respectively; nucleotides from the *AtMIR390a* or *OsMIR390* precursors are in black or grey, respectively, except those that were modified to preserve authentic *AtMIR390a* or *OsMIR390* precursor secondary structures that are in red.

(b-d) Representative images of plants expressing amiRNAs from *AtMIR390a-OsL* or *AtMIR390a-OsL* precursors.

(b) Adult control plant (*35S:GUS*) or plants expressing *35S:AtMIR390a-Ft-OsL* or *35S:AtMIR390a-Ft* plant with a delayed flowering phenotype.

(c) Ten days-old seedlings expressing *35S:AtMIR390a-OsL-Ch42* or *35S:AtMIR390a-Ch42* and showing bleaching phenotypes.

(d) Fifteen days-old control seedling (*35S:GUS*), or seedling expressing *35S:AtMIR390a-OsL-Trich* or *35S:AtMIR390a-Trich* with increased number of trichomes.

(e) Accumulation of amiRNAs in transgenic plants. One blot from three biological replicates is shown. Each biological replicate is a pool of at least 8 independent plants. U6 RNA blot is shown as a loading control.

(f) Mean relative level +/- s.e. of *A. thaliana FT*, *CH42*, *TRY*, *CPC* and *ETC2* mRNAs after normalization to *ACT2*, *CPB20*, *SAND* and *UBQ10*, as determined by quantitative real-time RT-PCR (*35S:GUS* = 1.0 in all comparisons).

(g) Mapping of amiRNA reads from *AtMIR390a-OsL* precursors expressed in transgenic plants. Analysis of amiRNA and amiRNA* reads in plants expressing amiR-AtFt (left), amiR-AtCh42 (center) and amiR-AtTrich (right), respectively. amiRNA guide and amiRNA* strands are highlighted in blue and green, respectively. Nucleotides from *AtMIR390a* or *OsMIR390* precursors are in black and grey, respectively, except those that were modified to preserve the corresponding authentic precursor secondary structure that are in red. Proportion of small RNA reads are plotted as stacked bar graphs. Small RNAs are color-coded by size
