# Supplementary material for: Highly specific gene silencing in a monocot species by artificial microRNAs derived from chimeric miRNA precursors
Source: Plant J. 2015 May 20;82(6):1061–75. doi: 10.1111/tpj.12835 (PMC4464980; doi:10.1111/tpj.12835)
Supplement: Supplementary file 11 — Figure S11. AmiRNA‐induced phenotype quantification in Arabidopsis transgenic plants expressing amiR‐AtFt (left) and amiR‐AtCh42 (right) from AtMIR390a or chimeric AtMIR390a‐OsL precursors. [file TPJ-82-1061-s011.pdf]

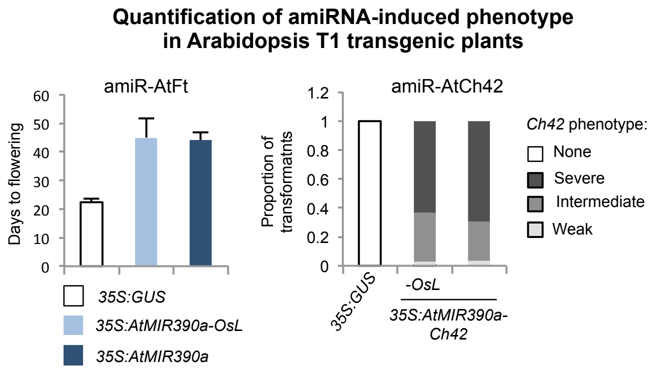

Figure S11. Quantification of amiRNA-induced phenotypes in Arabidopsis transgenic plants expressing amiR-AtFt (left) and amiR-AtCh42 (right) from *AtMIR390a* or chimeric *AtMIR390a-OsL* precursors.
