# Supplementary material for: Highly specific gene silencing in a monocot species by artificial microRNAs derived from chimeric miRNA precursors
Source: Plant J. 2015 May 20;82(6):1061–75. doi: 10.1111/tpj.12835 (PMC4464980; doi:10.1111/tpj.12835)
Supplement: Supplementary file 12 — Figure S12. Target accumulation determined by RNA‐Seq analysis in transgenic Brachypodium plants including 35S:OsMIR390‐AtL‐based or 35S:GUS constructs. [file TPJ-82-1061-s012.pdf]

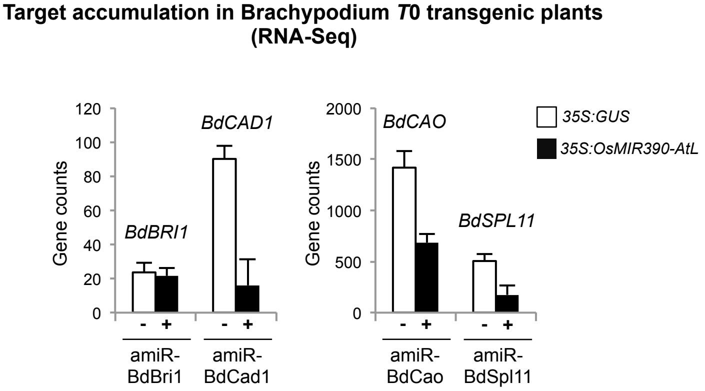

Figure S12. Target accumulation determined by RNA-Seq analysis in transgenic Brachypodium plants including *35S:OsMIR390-AtL*-based or *35S:GUS* constructs.
