# Supplementary material for: Highly specific gene silencing in a monocot species by artificial microRNAs derived from chimeric miRNA precursors
Source: Plant J. 2015 May 20;82(6):1061–75. doi: 10.1111/tpj.12835 (PMC4464980; doi:10.1111/tpj.12835)
Supplement: Supplementary file 13 — Table S1. MiRbase locus identifiers of Orzya sativa conserved MIRNA precursors. [file TPJ-82-1061-s013.doc]

| **Table S1.** MiRbase locus identifiers of *Orzya sativa* conserved *MIRNA* precursors. | |
| --- | --- |
| *MIRNA* precursor | Locus  Identifier |
| osa-MIR156a | [MI0000653](http://www.mirbase.org/cgi-bin/mirna_entry.pl?acc=MI0000653) |
| osa-MIR156b | [MI0000654](http://www.mirbase.org/cgi-bin/mirna_entry.pl?acc=MI0000654) |
| osa-MIR156c | [MI0000655](http://www.mirbase.org/cgi-bin/mirna_entry.pl?acc=MI0000655) |
| osa-MIR156d | [MI0000656](http://www.mirbase.org/cgi-bin/mirna_entry.pl?acc=MI0000656) |
| osa-MIR156e | [MI0000657](http://www.mirbase.org/cgi-bin/mirna_entry.pl?acc=MI0000657) |
| osa-MIR156f | [MI0000658](http://www.mirbase.org/cgi-bin/mirna_entry.pl?acc=MI0000658) |
| osa-MIR156g | [MI0000659](http://www.mirbase.org/cgi-bin/mirna_entry.pl?acc=MI0000659) |
| osa-MIR156h | [MI0000660](http://www.mirbase.org/cgi-bin/mirna_entry.pl?acc=MI0000660) |
| osa-MIR156i | [MI0000661](http://www.mirbase.org/cgi-bin/mirna_entry.pl?acc=MI0000661) |
| osa-MIR156j | [MI0000662](http://www.mirbase.org/cgi-bin/mirna_entry.pl?acc=MI0000662) |
| osa-MIR156k | [MI0001090](http://www.mirbase.org/cgi-bin/mirna_entry.pl?acc=MI0001090) |
| osa-MIR156l | [MI0001091](http://www.mirbase.org/cgi-bin/mirna_entry.pl?acc=MI0001091) |
| osa-MIR159a.1 | [MIMAT0001022](http://www.mirbase.org/cgi-bin/mature.pl?mature_acc=MIMAT0001022) |
| osa-MIR159b | [MI0001093](http://www.mirbase.org/cgi-bin/mirna_entry.pl?acc=MI0001093) |
| osa-MIR159c | [MI0001094](http://www.mirbase.org/cgi-bin/mirna_entry.pl?acc=MI0001094) |
| osa-MIR159d | [MI0001095](http://www.mirbase.org/cgi-bin/mirna_entry.pl?acc=MI0001095) |
| osa-MIR159e | [MI0001096](http://www.mirbase.org/cgi-bin/mirna_entry.pl?acc=MI0001096) |
| osa-MIR159f | [MI0001097](http://www.mirbase.org/cgi-bin/mirna_entry.pl?acc=MI0001097) |
| osa-MIR160a | [MI0000663](http://www.mirbase.org/cgi-bin/mirna_entry.pl?acc=MI0000663) |
| osa-MIR160b | [MI0000664](http://www.mirbase.org/cgi-bin/mirna_entry.pl?acc=MI0000664) |
| osa-MIR160c | [MI0000665](http://www.mirbase.org/cgi-bin/mirna_entry.pl?acc=MI0000665) |
| osa-MIR160d | [MI0000666](http://www.mirbase.org/cgi-bin/mirna_entry.pl?acc=MI0000666) |
| osa-MIR160e | [MI0001100](http://www.mirbase.org/cgi-bin/mirna_entry.pl?acc=MI0001100) |
| osa-MIR160f | [MI0001101](http://www.mirbase.org/cgi-bin/mirna_entry.pl?acc=MI0001101) |
| osa-MIR162a | [MI0000667](http://www.mirbase.org/cgi-bin/mirna_entry.pl?acc=MI0000667) |
| osa-MIR162b | [MI0001102](http://www.mirbase.org/cgi-bin/mirna_entry.pl?acc=MI0001102) |
| osa-MIR164a | [MI0000668](http://www.mirbase.org/cgi-bin/mirna_entry.pl?acc=MI0000668) |
| osa-MIR164b | [MI0000669](http://www.mirbase.org/cgi-bin/mirna_entry.pl?acc=MI0000669) |
| osa-MIR164c | [MI0001103](http://www.mirbase.org/cgi-bin/mirna_entry.pl?acc=MI0001103) |
| osa-MIR164d | [MI0001104](http://www.mirbase.org/cgi-bin/mirna_entry.pl?acc=MI0001104) |
| osa-MIR164e | [MI0001105](http://www.mirbase.org/cgi-bin/mirna_entry.pl?acc=MI0001105) |
| osa-MIR164f | [MI0001159](http://www.mirbase.org/cgi-bin/mirna_entry.pl?acc=MI0001159) |
| osa-MIR166a | [MI0000670](http://www.mirbase.org/cgi-bin/mirna_entry.pl?acc=MI0000670) |
| osa-MIR166b | [MI0000671](http://www.mirbase.org/cgi-bin/mirna_entry.pl?acc=MI0000671) |
| osa-MIR166c | [MI0000672](http://www.mirbase.org/cgi-bin/mirna_entry.pl?acc=MI0000672) |
| osa-MIR166d | [MI0000673](http://www.mirbase.org/cgi-bin/mirna_entry.pl?acc=MI0000673) |
| osa-MIR166e | [MI0000674](http://www.mirbase.org/cgi-bin/mirna_entry.pl?acc=MI0000674) |
| osa-MIR166f | [MI0000675](http://www.mirbase.org/cgi-bin/mirna_entry.pl?acc=MI0000675) |
| *MIRNA* precursor | Locus  Identifier |
| osa-MIR166g | [MI0001142](http://www.mirbase.org/cgi-bin/mirna_entry.pl?acc=MI0001142) |
| osa-MIR166h | [MI0001143](http://www.mirbase.org/cgi-bin/mirna_entry.pl?acc=MI0001143) |
| osa-MIR166i | [MI0001144](http://www.mirbase.org/cgi-bin/mirna_entry.pl?acc=MI0001144) |
| osa-MIR166j | [MI0001158](http://www.mirbase.org/cgi-bin/mirna_entry.pl?acc=MI0001158) |
| osa-MIR166k | [MI0001107](http://www.mirbase.org/cgi-bin/mirna_entry.pl?acc=MI0001107) |
| osa-MIR166l | [MI0001108](http://www.mirbase.org/cgi-bin/mirna_entry.pl?acc=MI0001108) |
| osa-MIR166m | [MI0001157](http://www.mirbase.org/cgi-bin/mirna_entry.pl?acc=MI0001157) |
| osa-MIR166n | [MIMAT0001088](http://www.mirbase.org/cgi-bin/mature.pl?mature_acc=MIMAT0001088) |
| osa-MIR167a | [MI0000676](http://www.mirbase.org/cgi-bin/mirna_entry.pl?acc=MI0000676) |
| osa-MIR167b | [MI0000677](http://www.mirbase.org/cgi-bin/mirna_entry.pl?acc=MI0000677) |
| osa-MIR167c | [MI0000678](http://www.mirbase.org/cgi-bin/mirna_entry.pl?acc=MI0000678) |
| osa-MIR167d | [MI0001109](http://www.mirbase.org/cgi-bin/mirna_entry.pl?acc=MI0001109) |
| osa-MIR167e | [MI0001110](http://www.mirbase.org/cgi-bin/mirna_entry.pl?acc=MI0001110) |
| osa-MIR167f | [MI0001111](http://www.mirbase.org/cgi-bin/mirna_entry.pl?acc=MI0001111) |
| osa-MIR167g | [MI0001112](http://www.mirbase.org/cgi-bin/mirna_entry.pl?acc=MI0001112) |
| osa-MIR167h | [MI0001113](http://www.mirbase.org/cgi-bin/mirna_entry.pl?acc=MI0001113) |
| osa-MIR167i | [MI0001114](http://www.mirbase.org/cgi-bin/mirna_entry.pl?acc=MI0001114) |
| osa-MIR167j | [MI0001156](http://www.mirbase.org/cgi-bin/mirna_entry.pl?acc=MI0001156) |
| osa-MIR168a | [MI0001115](http://www.mirbase.org/cgi-bin/mirna_entry.pl?acc=MI0001115) |
| osa-MIR169a | [MI0000679](http://www.mirbase.org/cgi-bin/mirna_entry.pl?acc=MI0000679) |
| osa-MIR169b | [MI0001117](http://www.mirbase.org/cgi-bin/mirna_entry.pl?acc=MI0001117) |
| osa-MIR169c | [MI0001118](http://www.mirbase.org/cgi-bin/mirna_entry.pl?acc=MI0001118) |
| osa-MIR169d | [MI0001119](http://www.mirbase.org/cgi-bin/mirna_entry.pl?acc=MI0001119) |
| osa-MIR169e | [MI0001120](http://www.mirbase.org/cgi-bin/mirna_entry.pl?acc=MI0001120) |
| osa-MIR169f | [MI0001121](http://www.mirbase.org/cgi-bin/mirna_entry.pl?acc=MI0001121) |
| osa-MIR169g | [MI0001122](http://www.mirbase.org/cgi-bin/mirna_entry.pl?acc=MI0001122) |
| osa-MIR169h | [MI0001123](http://www.mirbase.org/cgi-bin/mirna_entry.pl?acc=MI0001123) |
| osa-MIR169i | [MI0001124](http://www.mirbase.org/cgi-bin/mirna_entry.pl?acc=MI0001124) |
| osa-MIR169j | [MI0001125](http://www.mirbase.org/cgi-bin/mirna_entry.pl?acc=MI0001125) |
| osa-MIR169k | [MI0001126](http://www.mirbase.org/cgi-bin/mirna_entry.pl?acc=MI0001126) |
| osa-MIR169l | [MI0001127](http://www.mirbase.org/cgi-bin/mirna_entry.pl?acc=MI0001127) |
| osa-MIR169m | [MI0001128](http://www.mirbase.org/cgi-bin/mirna_entry.pl?acc=MI0001128) |
| osa-MIR169n | [MI0001129](http://www.mirbase.org/cgi-bin/mirna_entry.pl?acc=MI0001129) |
| osa-MIR169o | [MI0001130](http://www.mirbase.org/cgi-bin/mirna_entry.pl?acc=MI0001130) |
| osa-MIR169p | [MI0001131](http://www.mirbase.org/cgi-bin/mirna_entry.pl?acc=MI0001131) |
| osa-MIR169q | [MI0001132](http://www.mirbase.org/cgi-bin/mirna_entry.pl?acc=MI0001132) |
| osa-MIR171a | [MI0000680](http://www.mirbase.org/cgi-bin/mirna_entry.pl?acc=MI0000680) |
| osa-MIR171b | [MI0001133](http://www.mirbase.org/cgi-bin/mirna_entry.pl?acc=MI0001133) |
| osa-MIR171c | [MI0001134](http://www.mirbase.org/cgi-bin/mirna_entry.pl?acc=MI0001134) |
| osa-MIR171d | [MI0001135](http://www.mirbase.org/cgi-bin/mirna_entry.pl?acc=MI0001135) |
| *MIRNA* precursor | Locus  Identifier |
| osa-MIR171e | [MI0001136](http://www.mirbase.org/cgi-bin/mirna_entry.pl?acc=MI0001136) |
| osa-MIR171f | [MI0001137](http://www.mirbase.org/cgi-bin/mirna_entry.pl?acc=MI0001137) |
| osa-MIR171g | [MI0001138](http://www.mirbase.org/cgi-bin/mirna_entry.pl?acc=MI0001138) |
| osa-MIR171h | [MI0001147](http://www.mirbase.org/cgi-bin/mirna_entry.pl?acc=MI0001147) |
| osa-MIR171i | [MI0001155](http://www.mirbase.org/cgi-bin/mirna_entry.pl?acc=MI0001155) |
| osa-MIR172a | [MI0001139](http://www.mirbase.org/cgi-bin/mirna_entry.pl?acc=MI0001139) |
| osa-MIR172b | [MI0001140](http://www.mirbase.org/cgi-bin/mirna_entry.pl?acc=MI0001140) |
| osa-MIR172c | [MI0001141](http://www.mirbase.org/cgi-bin/mirna_entry.pl?acc=MI0001141) |
| osa-MIR172d | [MI0001154](http://www.mirbase.org/cgi-bin/mirna_entry.pl?acc=MI0001154) |
| osa-MIR319a | [MI0001098](http://www.mirbase.org/cgi-bin/mirna_entry.pl?acc=MI0001098) |
| osa-MIR319b | [MI0001099](http://www.mirbase.org/cgi-bin/mirna_entry.pl?acc=MI0001099) |
| osa-MIR390 | [MI0001690](http://www.mirbase.org/cgi-bin/mirna_entry.pl?acc=MI0001690) |
| osa-MIR393 | [MI0001026](http://www.mirbase.org/cgi-bin/mirna_entry.pl?acc=MI0001026) |
| osa-MIR393b | [MI0001148](http://www.mirbase.org/cgi-bin/mirna_entry.pl?acc=MI0001148) |
| osa-MIR394 | [MI0001027](http://www.mirbase.org/cgi-bin/mirna_entry.pl?acc=MI0001027) |
| osa-MIR395a | [MI0001042](http://www.mirbase.org/cgi-bin/mirna_entry.pl?acc=MI0001042) |
| osa-MIR395b | [MI0001028](http://www.mirbase.org/cgi-bin/mirna_entry.pl?acc=MI0001028) |
| osa-MIR395c | [MI0001041](http://www.mirbase.org/cgi-bin/mirna_entry.pl?acc=MI0001041) |
| osa-MIR395d | [MI0001029](http://www.mirbase.org/cgi-bin/mirna_entry.pl?acc=MI0001029) |
| osa-MIR395e | [MI0001030](http://www.mirbase.org/cgi-bin/mirna_entry.pl?acc=MI0001030) |
| osa-MIR395f | [MI0001043](http://www.mirbase.org/cgi-bin/mirna_entry.pl?acc=MI0001043) |
| osa-MIR395g | [MI0001031](http://www.mirbase.org/cgi-bin/mirna_entry.pl?acc=MI0001031) |
| osa-MIR395h | [MI0001032](http://www.mirbase.org/cgi-bin/mirna_entry.pl?acc=MI0001032) |
| osa-MIR395i | [MI0001033](http://www.mirbase.org/cgi-bin/mirna_entry.pl?acc=MI0001033) |
| osa-MIR395j | [MI0001034](http://www.mirbase.org/cgi-bin/mirna_entry.pl?acc=MI0001034) |
| osa-MIR395k | [MI0001035](http://www.mirbase.org/cgi-bin/mirna_entry.pl?acc=MI0001035) |
| osa-MIR395l | [MI0001036](http://www.mirbase.org/cgi-bin/mirna_entry.pl?acc=MI0001036) |
| osa-MIR395m | [MI0005084](http://www.mirbase.org/cgi-bin/mirna_entry.pl?acc=MI0005084) |
| osa-MIR395n | [MI0005085](http://www.mirbase.org/cgi-bin/mirna_entry.pl?acc=MI0005085) |
| osa-MIR395o | [MI0005086](http://www.mirbase.org/cgi-bin/mirna_entry.pl?acc=MI0005086) |
| osa-MIR395p | [MI0005087](http://www.mirbase.org/cgi-bin/mirna_entry.pl?acc=MI0005087) |
| osa-MIR395q | [MI0005088](http://www.mirbase.org/cgi-bin/mirna_entry.pl?acc=MI0005088) |
| osa-MIR395r | [MI0005092](http://www.mirbase.org/cgi-bin/mirna_entry.pl?acc=MI0005092) |
| osa-MIR395s | [MI0001037](http://www.mirbase.org/cgi-bin/mirna_entry.pl?acc=MI0001037) |
| osa-MIR395t | [MI0001038](http://www.mirbase.org/cgi-bin/mirna_entry.pl?acc=MI0001038) |
| osa-MIR395u | [MI0001044](http://www.mirbase.org/cgi-bin/mirna_entry.pl?acc=MI0001044) |
| osa-MIR395v | [MI0005090](http://www.mirbase.org/cgi-bin/mirna_entry.pl?acc=MI0005090) |
| osa-MIR395w | [MI0005091](http://www.mirbase.org/cgi-bin/mirna_entry.pl?acc=MI0005091) |
| osa-MIR396a | [MI0001046](http://www.mirbase.org/cgi-bin/mirna_entry.pl?acc=MI0001046) |
| osa-MIR396b | [MI0001047](http://www.mirbase.org/cgi-bin/mirna_entry.pl?acc=MI0001047) |
| *MIRNA* precursor | Locus  Identifier |
| osa-MIR396c | [MI0001048](http://www.mirbase.org/cgi-bin/mirna_entry.pl?acc=MI0001048) |
| osa-MIR396d | [MI0013049](http://www.mirbase.org/cgi-bin/mirna_entry.pl?acc=MI0013049) |
| osa-MIR396e | [MI0001703](http://www.mirbase.org/cgi-bin/mirna_entry.pl?acc=MI0001703) |
| osa-MIR396f | [MI0010563](http://www.mirbase.org/cgi-bin/mirna_entry.pl?acc=MI0010563) |
| osa-MIR396h | [MI0013048](http://www.mirbase.org/cgi-bin/mirna_entry.pl?acc=MI0013048) |
| osa-MIR397a | [MI0001049](http://www.mirbase.org/cgi-bin/mirna_entry.pl?acc=MI0001049) |
| osa-MIR397b | [MI0001050](http://www.mirbase.org/cgi-bin/mirna_entry.pl?acc=MI0001050) |
| osa-MIR398a | [MI0001051](http://www.mirbase.org/cgi-bin/mirna_entry.pl?acc=MI0001051) |
| osa-MIR398b | [MI0001052](http://www.mirbase.org/cgi-bin/mirna_entry.pl?acc=MI0001052) |
| osa-MIR399a | [MI0001053](http://www.mirbase.org/cgi-bin/mirna_entry.pl?acc=MI0001053) |
| osa-MIR399b | [MI0001054](http://www.mirbase.org/cgi-bin/mirna_entry.pl?acc=MI0001054) |
| osa-MIR399c | [MI0001055](http://www.mirbase.org/cgi-bin/mirna_entry.pl?acc=MI0001055) |
| osa-MIR399d | [MI0001056](http://www.mirbase.org/cgi-bin/mirna_entry.pl?acc=MI0001056) |
| osa-MIR399e | [MI0001057](http://www.mirbase.org/cgi-bin/mirna_entry.pl?acc=MI0001057) |
| osa-MIR399f | [MI0001058](http://www.mirbase.org/cgi-bin/mirna_entry.pl?acc=MI0001058) |
| osa-MIR399g | [MI0001059](http://www.mirbase.org/cgi-bin/mirna_entry.pl?acc=MI0001059) |
| osa-MIR399h | [MI0001060](http://www.mirbase.org/cgi-bin/mirna_entry.pl?acc=MI0001060) |
| osa-MIR399i | [MI0001061](http://www.mirbase.org/cgi-bin/mirna_entry.pl?acc=MI0001061) |
| osa-MIR399j | [MI0001062](http://www.mirbase.org/cgi-bin/mirna_entry.pl?acc=MI0001062) |
| osa-MIR399k | [MI0001063](http://www.mirbase.org/cgi-bin/mirna_entry.pl?acc=MI0001063) |
| osa-MIR408 | [MI0001149](http://www.mirbase.org/cgi-bin/mirna_entry.pl?acc=MI0001149) |
| osa-MIR528 | [MI0003201](http://www.mirbase.org/cgi-bin/mirna_entry.pl?acc=MI0003201) |
| osa-MIR827 | [MI0010490](http://www.mirbase.org/cgi-bin/mirna_entry.pl?acc=MI0010490) |
