# Supplementary material for: Highly specific gene silencing in a monocot species by artificial microRNAs derived from chimeric miRNA precursors
Source: Plant J. 2015 May 20;82(6):1061–75. doi: 10.1111/tpj.12835 (PMC4464980; doi:10.1111/tpj.12835)
Supplement: Supplementary file 14 — Table S2. MiRbase locus identifiers of plant MIR390 precursors. [file TPJ-82-1061-s014.doc]

| **Table S2.** MiRbase locus identifiers of plant *MIR390* precursors. | |
| --- | --- |
| *MIRNA* precursor | Locus  Identifier |
| aly-MIR390a | [MI0014569](http://www.mirbase.org/cgi-bin/mirna_entry.pl?acc=MI0014569) |
| aly-MIR390b | [MI0014570](http://www.mirbase.org/cgi-bin/mirna_entry.pl?acc=MI0014570) |
| ath-MIR390a | [MI0001000](http://www.mirbase.org/cgi-bin/mirna_entry.pl?acc=MI0001000) |
| ath-MIR390b | [MI0001001](http://www.mirbase.org/cgi-bin/mirna_entry.pl?acc=MI0001001) |
| bna-MIR390a | [MI0006447](http://www.mirbase.org/cgi-bin/mirna_entry.pl?acc=MI0006447) |
| bna-MIR390b | [MI0006448](http://www.mirbase.org/cgi-bin/mirna_entry.pl?acc=MI0006448) |
| bna-MIR390c | [MI0006449](http://www.mirbase.org/cgi-bin/mirna_entry.pl?acc=MI0006449) |
| cca-MIR390 | [MI0021077](http://www.mirbase.org/cgi-bin/mirna_entry.pl?acc=MI0021077) |
| cme-MIR390a | [MI0023238](http://www.mirbase.org/cgi-bin/mirna_entry.pl?acc=MI0023238) |
| cme-MIR390b | [MI0018164](http://www.mirbase.org/cgi-bin/mirna_entry.pl?acc=MI0018164) |
| cme-MIR390c | [MI0023239](http://www.mirbase.org/cgi-bin/mirna_entry.pl?acc=MI0023239) |
| cme-MIR390d | [MI0023237](http://www.mirbase.org/cgi-bin/mirna_entry.pl?acc=MI0023237) |
| csi-MIR390 | [MI0013317](http://www.mirbase.org/cgi-bin/mirna_entry.pl?acc=MI0013317) |
| ghr-MIR390a | [MI0005647](http://www.mirbase.org/cgi-bin/mirna_entry.pl?acc=MI0005647) |
| ghr-MIR390b | [MI0005648](http://www.mirbase.org/cgi-bin/mirna_entry.pl?acc=MI0005648) |
| ghr-MIR390c | [MI0005649](http://www.mirbase.org/cgi-bin/mirna_entry.pl?acc=MI0005649) |
| gma-MIR390a | [MI0007214](http://www.mirbase.org/cgi-bin/mirna_entry.pl?acc=MI0007214) |
| gma-MIR390b | [MI0007215](http://www.mirbase.org/cgi-bin/mirna_entry.pl?acc=MI0007215) |
| gma-MIR390c | [MI0017845](http://www.mirbase.org/cgi-bin/mirna_entry.pl?acc=MI0017845) |
| gma-MIR390d | [MI0021700](http://www.mirbase.org/cgi-bin/mirna_entry.pl?acc=MI0021700) |
| gma-MIR390e | [MI0021701](http://www.mirbase.org/cgi-bin/mirna_entry.pl?acc=MI0021701) |
| gma-MIR390f | [MI0021702](http://www.mirbase.org/cgi-bin/mirna_entry.pl?acc=MI0021702) |
| gma-MIR390g | [MI0021703](http://www.mirbase.org/cgi-bin/mirna_entry.pl?acc=MI0021703) |
| hex-MIR390a | [MI0022249](http://www.mirbase.org/cgi-bin/mirna_entry.pl?acc=MI0022249) |
| hex-MIR390b | [MI0022250](http://www.mirbase.org/cgi-bin/mirna_entry.pl?acc=MI0022250) |
| mdm-MIR390a | [MI0023073](http://www.mirbase.org/cgi-bin/mirna_entry.pl?acc=MI0023073) |
| mdm-MIR390b | [MI0023074](http://www.mirbase.org/cgi-bin/mirna_entry.pl?acc=MI0023074) |
| mdm-MIR390c | [MI0023075](http://www.mirbase.org/cgi-bin/mirna_entry.pl?acc=MI0023075) |
| mdm-MIR390d | [MI0023076](http://www.mirbase.org/cgi-bin/mirna_entry.pl?acc=MI0023076) |
| mdm-MIR390e | [MI0023077](http://www.mirbase.org/cgi-bin/mirna_entry.pl?acc=MI0023077) |
| mdm-MIR390f | [MI0023078](http://www.mirbase.org/cgi-bin/mirna_entry.pl?acc=MI0023078) |
| mtr-MIR390 | [MI0005586](http://www.mirbase.org/cgi-bin/mirna_entry.pl?acc=MI0005586) |
| nta-MIR390a | [MI0021391](http://www.mirbase.org/cgi-bin/mirna_entry.pl?acc=MI0021391) |
| nta-MIR390b | [MI0021392](http://www.mirbase.org/cgi-bin/mirna_entry.pl?acc=MI0021392) |
| nta-MIR390c | [MI0021393](http://www.mirbase.org/cgi-bin/mirna_entry.pl?acc=MI0021393) |
| pde-MIR390 | [MI0022095](http://www.mirbase.org/cgi-bin/mirna_entry.pl?acc=MI0022095) |
| pta-MIR390 | [MI0005787](http://www.mirbase.org/cgi-bin/mirna_entry.pl?acc=MI0005787) |
| ptc-MIR390a | [MI0002305](http://www.mirbase.org/cgi-bin/mirna_entry.pl?acc=MI0002305) |
| *MIRNA* precursor | Locus  Identifier |
| ptc-MIR390b | [MI0002306](http://www.mirbase.org/cgi-bin/mirna_entry.pl?acc=MI0002306) |
| ptc-MIR390c | [MI0002307](http://www.mirbase.org/cgi-bin/mirna_entry.pl?acc=MI0002307) |
| ptc-MIR390d | [MI0002308](http://www.mirbase.org/cgi-bin/mirna_entry.pl?acc=MI0002308) |
| rco-MIR390a | [MI0013410](http://www.mirbase.org/cgi-bin/mirna_entry.pl?acc=MI0013410) |
| rco-MIR390b | [MI0013411](http://www.mirbase.org/cgi-bin/mirna_entry.pl?acc=MI0013411) |
| tcc-MIR390a | [MI0017503](http://www.mirbase.org/cgi-bin/mirna_entry.pl?acc=MI0017503) |
| tcc-MIR390b | [MI0017504](http://www.mirbase.org/cgi-bin/mirna_entry.pl?acc=MI0017504) |
| vvi-MIR390 | [MI0006552](http://www.mirbase.org/cgi-bin/mirna_entry.pl?acc=MI0006552) |
