# Supplementary material for: Highly specific gene silencing in a monocot species by artificial microRNAs derived from chimeric miRNA precursors
Source: Plant J. 2015 May 20;82(6):1061–75. doi: 10.1111/tpj.12835 (PMC4464980; doi:10.1111/tpj.12835)
Supplement: Supplementary file 15 — Table S3. AmiRNA phenotypic penetrance in Brachypodium T0 transgenic plants. [file TPJ-82-1061-s015.doc]

| **Table S3**: AmiRNA phenotypic penetrance in Brachypodium T0 transgenic plants. | | |
| --- | --- | --- |
| Construct | T0 analyzed | Phenotypic penetrance^a^ |
| *35S:OsMIR390-Bri1* | 11 | 64% |
| *35S:OsMIR390-AtL-Bri1* | 20 | 80% |
| *UBI:OsMIR390-AtL-Bri1* | 22 | 32% |
| *35S:OsMIR390-Cad1* | 52 | 94% |
| *35S:OsMIR390-AtL-Cad1* | 27 | 100% |
| *35S:OsMIR390-Cao* | 12 | 100% |
| *35S:OsMIR390-AtL-Cao* | 27 | 100% |
| *UBI:OsMIR390-AtL-Cao* | 32 | 53% |
| *35S:OsMIR390-Spl11* | 22 | 95% |
| *35S:OsMIR390-AtL-Spl11* | 43 | 91% |
| *UBI:OsMIR390-AtL-Spl11* | 13 | 61% |
| ^a^The Bri1 phenotype was defined as a shorter height and presence of splindly leaves in amiR-Bri1 transformants when compared to transformants of the *35S:GUS* control set.  The Cad1 phenotype was defined as the presence of brown to red colorations in stems and nodes in amiR-Cad transformants.  The Cao phenotype was defined as a lighter green color amiR-Cao1 transformants when compared to transformants of the *35S:GUS* control set.  The Spl11 phenotype was defined as the presence of necrotic areas in leaves from amiR-Spl11 transformants. | | |
