# Supplementary material for: Highly specific gene silencing in a monocot species by artificial microRNAs derived from chimeric miRNA precursors
Source: Plant J. 2015 May 20;82(6):1061–75. doi: 10.1111/tpj.12835 (PMC4464980; doi:10.1111/tpj.12835)
Supplement: Supplementary file 16 — Table S4. AmiRNA phenotypic penetrance in Brachypodium T1 transgenic plants. [file TPJ-82-1061-s016.doc]

| **Table S4**: AmiRNA phenotypic penetrance in Brachypodium T1 transgenic plants. | | |
| --- | --- | --- |
| Construct | T1 analyzed | Phenotypic penetrance^a^ |
| *35S:OsMIR390-Bri1* | 1 | 100% |
| *35S:OsMIR390-AtL-Bri1* | 2 | 50% |
| *35S:OsMIR390-AtL-Cad1* | 6 | 100% |
| *35S:OsMIR390-AtL-Cao* | 2 | 100% |
| *35S:OsMIR390-AtL-Spl11* | 4 | 100% |
| *UBI:OsMIR390-AtL-Spl11* | 4 | 100% |
| ^a^The Bri1 phenotype was defined as a shorter height and presence of splindly leaves in amiR-Bri1 transformants when compared to transformants of the *35S:GUS* control set.  The Cao1 phenotype was defined as a lighter green color amiR-Cao1 transformants when compared to transformants of the *35S:GUS* control set.  The Cad phenotype was defined as the presence of brown to red colorations in stems and nodes in amiR-Cad transformants.  The Spl11 phenotype was defined as the presence of necrotic areas in leaves from amiR-Spl11 transformants. | | |
