# Supplementary material for: Highly specific gene silencing in a monocot species by artificial microRNAs derived from chimeric miRNA precursors
Source: Plant J. 2015 May 20;82(6):1061–75. doi: 10.1111/tpj.12835 (PMC4464980; doi:10.1111/tpj.12835)
Supplement: Supplementary file 17 — Table S5. AmiRNA phenotypic penetrance in Arabidopsis T1 transgenic plants. [file TPJ-82-1061-s017.doc]

| **Table S5**: AmiRNA phenotypic penetrance in Arabidopsis T1 transgenic plants. | | |
| --- | --- | --- |
| Construct | T1 analyzed | Phenotypic penetrance^a^ |
| *35S:AtMIR390a-Ft* | 64 | 100% |
| *35S:AtMIR390a-OsL-Ft* | 44 | 100% |
| *35S:AtMIR390a-Ch42* | 406 | 100%  3% weak  28% intermediate  69% severe |
| *35S:AtMIR390a-OsL-Ch42* | 267 | 98%  3% weak  33% intermediate  64% severe |
| *35S:AtMIR390a-Trich* | 45 | 93%  12% *try cpc* type |
| *35S:AtMIR390a-OsL-Trich* | 69 | 99%  9% *try cpc* type |
| ^a^The Ft phenotype was defined as a higher ‘days to flowering’ value when compared to the average ‘days to flowering’ value of the *35S:GUS* control set.  The Ch42 phenotype was scored in 10 days-old seedling and was considered ‘weak’, ‘intermediate’ or ‘severe’ if seedlings have >2 leaves, exactly 2 leaves or no leaves (only 2 cotyledons), respectively.  The Trich phenotype was defined as a higher number of trichomes when compared to transformants of the 3*5S:GUS* control set. Plants with a Trich phenotype were considered *‘try cpc* type’ if they resembled the Arabidopsis *try cpc* double mutant. | | |
