# Supplementary material for: Highly specific gene silencing in a monocot species by artificial microRNAs derived from chimeric miRNA precursors
Source: Plant J. 2015 May 20;82(6):1061–75. doi: 10.1111/tpj.12835 (PMC4464980; doi:10.1111/tpj.12835)
Supplement: Supplementary file 18 — Table S6. AmiRNA phenotypic penetrance in Arabidopsis T2 transgenic plants. [file TPJ-82-1061-s018.doc]

| **Table S6**: AmiRNA phenotypic penetrance in Arabidopsis T2 transgenic plants. | | |
| --- | --- | --- |
| Construct | T2 analyzed | Phenotypic penetrance^a^ |
| *35S:AtMIR390a-Ft* | 5 | 100% |
| *35S:AtMIR390a-OsL-Ft* | 5 | 100% |
| *35S:AtMIR390a-Trich* | 10 | 90% |
| *35S:AtMIR390a-OsL-Trich* | 10 | 90% |
| ^a^The Ft phenotype was defined as a higher ‘days to flowering’ value when compared to the average ‘days to flowering’ value of the *35S:GUS* control set.  The Trich phenotype was defined as a higher number of trichomes when compared to transformants of the 3*5S:GUS* control set. | | |
