# Supplementary material for: Highly specific gene silencing in a monocot species by artificial microRNAs derived from chimeric miRNA precursors
Source: Plant J. 2015 May 20;82(6):1061–75. doi: 10.1111/tpj.12835 (PMC4464980; doi:10.1111/tpj.12835)
Supplement: Supplementary file 19 — Table S7. DNA, LNA and RNA oligonucleotides. [file TPJ-82-1061-s019.doc]

| **Table S7.** DNA, LNA and RNA oligonucleotides^1^. | |
| --- | --- |
| Oligonucleotide Name | Sequence |
| 3'PCR primer i1 | CAAGCAGAAGACGGCATACGAACATCGATTGATGGTGCCTACAG |
| 3'PCR primer i2 | CAAGCAGAAGACGGCATACGAGTGATCATTGATGGTGCCTACAG |
| 3'PCR primer i3 | CAAGCAGAAGACGGCATACGACATCTGATTGATGGTGCCTACAG |
| 3'PCR primer i4 | CAAGCAGAAGACGGCATACGAAACGTAATTGATGGTGCCTACAG |
| 3'PCR primer i5 | CAAGCAGAAGACGGCATACGATGGTAAATTGATGGTGCCTACAG |
| 3'PCR primer i6 | CAAGCAGAAGACGGCATACGATACAGTATTGATGGTGCCTACAG |
| 3'PCR primer i7 | CAAGCAGAAGACGGCATACGACGTGATATTGATGGTGCCTACAG |
| 3'PCR primer i8 | CAAGCAGAAGACGGCATACGAACAAGTATTGATGGTGCCTACAG |
| 3'PCR primer i10 | CAAGCAGAAGACGGCATACGACTAGCAATTGATGGTGCCTACAG |
| 3'PCR primer i11 | CAAGCAGAAGACGGCATACGATACAAGATTGATGGTGCCTACAG |
| 5'PCR primer P5 | AATGATACGGCGACCACCGACAGGTTCAGAGTTCTACAGTCCGA |
| Adaptor 1 | ACACTCTTTCCCTACACGACGCTCTTCCGATC*T |
| Adaptor 2 | /5Phos/G*ATCGGAAGAGCGGTTCAGCAGGAATGCCGAG |
| AtMIR390a-OsL-F | TGTAAAGCTCAGGAGGGATAGCGCCTCGAAATCAAACTAGGCGCTATCCATCCTGAGTTT |
| AtMIR390a-OsL-R | AATGAAACTCAGGATGGATAGCGCCTAGTTTGATTTCGAGGCGCTATCCCTCCTGAGCTT |
| AtMIR390a-OsL-173-21-F | TGTATTCGCTTGCAGAGAGAAATCATCGAAATCAAACTATGATTTCTCTGTGTAAGCGAA |
| AtMIR390a-OsL-173-21-R | AATGTTCGCTTACACAGAGAAATCATAGTTTGATTTCGATGATTTCTCTCTGCAAGCGAA |
| AtMIR390a-OsL-472-21-F | TGTATTTTTCCTACTCCGCCCATACTCGAAATCAAACTAGTATGGGCGGCGTAGGAAAAA |
| AtMIR390a-OsL-472-21-R | AATGTTTTTCCTACGCCGCCCATACTAGTTTGATTTCGAGTATGGGCGGAGTAGGAAAAA |
| AtMIR390a-OsL-828-21-F | TGTATCTTGCTTAAATGAGTATTCCTCGAAATCAAACTAGGAATACTCAGTTAAGCAAGA |
| AtMIR390a-OsL-828-21-R | AATGTCTTGCTTAACTGAGTATTCCTAGTTTGATTTCGAGGAATACTCATTTAAGCAAGA |
| AtMIR390a-OsL-AtCh42-F | TGTATTAAGTGTCACGGAAATCCCTTCGAAATCAAACTAAGGGATTTCCTTGACACTTAA |
| AtMIR390a-OsL-AtCh42-R | AATGTTAAGTGTCAAGGAAATCCCTTAGTTTGATTTCGAAGGGATTTCCGTGACACTTAA |
| AtMIR390a-OsL-AtFt-F | TGTATTGGTTATAAAGGAAGAGGCCTCGAAATCAAACTAGGCCTCTTCCGTTATAACCAA |
| AtMIR390a-OsL-AtFt-R | AATGTTGGTTATAACGGAAGAGGCCTAGTTTGATTTCGAGGCCTCTTCCTTTATAACCAA |
| AtMIR390a-OsL-AtTrich-F | TGTATCCCATTCGATACTGCTCGCCTCGAAATCAAACTAGGCGAGCAGTCTCGAATGGGA |
| AtMIR390a-OsL-AtTrich-R | AATGTCCCATTCGAGACTGCTCGCCTAGTTTGATTTCGAGGCGAGCAGTATCGAATGGGA |
| Bradi1g30690-510-F | ACCAAAATTACCGAGACGAGCAGCAG |
| Bradi1g30690-666-R | AGGCCTGTCATGTGATGGTTCTTGC |
| Bradi1g41825-987-F | CCGTGCTAAAACACTTGCAAGGAAGC |
| Bradi1g41825-1180-R | CCTCACCAGGTGCCAACGATACATT |
| Bradi1g54680-821-F | TCTCATCATCATCCTGTCGGTGTGC |
| Bradi1g54680-1010-R | CACGACATTAGGACACCCGGATCA |
| Bradi1g61790-2634-F | GAACTTCTCCGCCATCGTGGAGTCT |
| Bradi1g61790-2876-R | CATTGATGGGCAACTCCCTGTCTCTC |
| Bradi1g62572-1091-F | ACGACTGCCCGCCCTCATCTACT |
| Bradi1g62572-1221-R | CAGCAAAGGAAGCCCGCTGAATTAGT |
| Bradi1g72485-602-F | AACGAAGGAGAAGGGTCTGCGTCTG |
| Bradi1g72485-847-R | CTGCACCTCCTCCCTCACCATCTC |
| Bradi2g48280-2698-F | GGGGTAAAACTGAACTGGCCAGCAA |
| Bradi2g48280-2884-R | CCACACTCATCATCCTCGCCATACC |
| Bradi2g61500-1136-F | CCATCCCTTCTCTGCTGCCTCCTT |
| Bradi2g61500-1335-R | CCCTTGGAGCCCAGAAGTAGGTGTC |
| Bradi3g06480-1047-F | TGCGTCGAGAAAGGGCTTACTTCTCA |
| Bradi3g06480-1248-R | CACGCACGCACGCACTCTACCTA |
| Bradi3g07850-1195-F | TGTGCAGATACAATGGTGGGTGACAG |
| Bradi3g07850-1334-R | GAGCTGTCCAGACCGGTGGAGATTT |
| Bradi4g04270-1581-F | TGATTATCGGGGGAACAGGGGCTAT |
| Bradi4g04270-1750-R | CACCAGACCCATGATTAGTGGCACA |
| Bradi4g09648-1378-F | GATGGCTTGTCTCAGCTCCCATGTTT |
| Bradi4g09648-1579-R | CTTGCTCCTCCCACTCCCACTCTTC |
| Bradi4g17230-1460-F | GTTGCAAGCTGCTGGTGAAGTCGAT |
| Bradi4g17230-1581-R | CACGGACGTACGACGACACATACAAA |
| Bradi4g21000-201-F | TCCGTATCCAGAAAGCCAAAGCTCAC |
| Bradi4g21000-490-R | TTGCTGAACTGGAGGAGGAAGACGA |
| BsaI-OsMIR390-F | CACCGAGCTCGAGATGTTTTGAGGAAGGGTATGGAACAATCCTTGAGAGACCGGTCTCACATGGTTTGTTCTTACCACACGACCAATTAAATCGAGCTC |
| BsaI-OsMIR390-R | GAGCTCGATTTAATTGGTCGTGTGGTAAGAACAAACCATGTGAGACCGGTCTCTCAAGGATTGTTCCATACCCTTCCTCAAAACATCTCGAGCTCGGTG |
| GeneRacer 3' Primer | GGACACTGACATGGACTGAAGGAGTA |
| GeneRacer 5' Nested Primer | GGACACTGACATGGACTGAAGGAGTA |
| GeneRacer 5' primer | CGACTGGAGCACGAGGACACTGA |
| Oligonucleotide Name | Sequence |
| GeneRacer Oligo dT Primer | GCTGTCAACGATACGCTACGTAACGGCATGACAGTG(T)_24_ |
| GeneRacer RNA Oligo | CGACUGGAGCACGAGGACACUGACAUGGACUGAAGGAGUAGAAA |
| OsMIR390-F | CTTGAAGCTCAGGAGGGATAGCGCCTCGAAATCAAACTAGGCGCTATCTATCCTGAGCTC |
| OsMIR390-R | CATGGAGCTCAGGATAGATAGCGCCTAGTTTGATTTCGAGGCGCTATCCCTCCTGAGCTT |
| OsMIR390-AtL-F | CTTGAAGCTCAGGAGGGATAGCGCCATGATGATCACATTCGTTATCTATTTTTTGGCGCTATCTATCCTGAGCTC |
| OsMIR390-AtL-R | CATGGAGCTCAGGATAGATAGCGCCAAAAAATAGATAACGAATGTGATCATCATGGCGCTATCCCTCCTGAGCTT |
| OsMIR390-173-21-F | CTTGTTCGCTTGCAGAGAGAAATCATCGAAATCAAACTATGATTTCTCTGTGTAAGCGAC |
| OsMIR390-173-21-R | CATGGTCGCTTACACAGAGAAATCATAGTTTGATTTCGATGATTTCTCTCTGCAAGCGAA |
| OsMIR390-AtL-173-21-F | CTTGTTCGCTTGCAGAGAGAAATCAATGATGATCACATTCGTTATCTATTTTTTTGATTTCTCTGTGTAAGCGAC |
| OsMIR390-AtL-173-21-R | CATGGTCGCTTACACAGAGAAATCAAAAAAATAGATAACGAATGTGATCATCATTGATTTCTCTCTGCAAGCGAA |
| OsMIR390-472-21-F | CTTGTTTTTCCTACTCCGCCCATACTCGAAATCAAACTAGTATGGGCGGCGTAGGAAAAC |
| OsMIR390-472-21-R | CATGGTTTTCCTACGCCGCCCATACTAGTTTGATTTCGAGTATGGGCGGAGTAGGAAAAA |
| OsMIR390-AtL-472-21-F | CTTGTTTTTCCTACTCCGCCCATACATGATGATCACATTCGTTATCTATTTTTTGTATGGGCGGCGTAGGAAAAC |
| OsMIR390-AtL-472-21-R | CATGGTTTTCCTACGCCGCCCATACAAAAAATAGATAACGAATGTGATCATCATGTATGGGCGGAGTAGGAAAAA |
| OsMIR390-828-21-F | CTTGTCTTGCTTAAATGAGTATTCCTCGAAATCAAACTAGGAATACTCAGTTAAGCAAGC |
| OsMIR390-828-21-R | CATGGCTTGCTTAACTGAGTATTCCTAGTTTGATTTCGAGGAATACTCATTTAAGCAAGA |
| OsMIR390-AtL-828-21-F | CTTGTCTTGCTTAAATGAGTATTCCATGATGATCACATTCGTTATCTATTTTTTGGAATACTCAGTTAAGCAAGC |
| OsMIR390-AtL-828-21-R | CATGGCTTGCTTAACTGAGTATTCCAAAAAATAGATAACGAATGTGATCATCATGGAATACTCATTTAAGCAAGA |
| OsMIR390-AtL-BdBri1-F | CTTGTCGCAATCTTCCGCCTTGCTCATGATGATCACATTCGTTATCTATTTTTTGAGCAAGGCGTAAGATTGCGC |
| OsMIR390-AtL-BdBri1-R | CATGGCGCAATCTTACGCCTTGCTCAAAAAATAGATAACGAATGTGATCATCATGAGCAAGGCGGAAGATTGCGA |
| OsMIR390-AtL-BdCad1-F | CTTGTCGATCTGAGAAGTAAGCCCAATGATGATCACATTCGTTATCTATTTTTTTGGGCTTACTGCTCAGATCGC |
| OsMIR390-AtL-BdCad1-R | CATGGCGATCTGAGCAGTAAGCCCAAAAAAATAGATAACGAATGTGATCATCATTGGGCTTACTTCTCAGATCGA |
| OsMIR390-AtL-BdCao-F | CTTGTCTGCATGGATTGTAAACCCAATGATGATCACATTCGTTATCTATTTTTTTGGGTTTACACTCCATGCAGC |
| OsMIR390-AtL-BdCao-R | CATGGCTGCATGGAGTGTAAACCCAAAAAAATAGATAACGAATGTGATCATCATTGGGTTTACAATCCATGCAGA |
| OsMIR390-AtL-BdSpl11-F | CTTGTTAGCAACACTACAAGGGCACATGATGATCACATTCGTTATCTATTTTTTGTGCCCTTGTCGTGTTGCTAC |
| OsMIR390-AtL-BdSpl11-R | CATGGTAGCAACACGACAAGGGCACAAAAAATAGATAACGAATGTGATCATCATGTGCCCTTGTAGTGTTGCTAA |
| OsMIR390-BdBri1-F | CTTGTCGCAATCTTCCGCCTTGCTCTCGAAATCAAACTAGAGCAAGGCGTAAGATTGCGC |
| OsMIR390-BdBri1-R | CATGGCGCAATCTTACGCCTTGCTCTAGTTTGATTTCGAGAGCAAGGCGGAAGATTGCGA |
| OsMIR390-BdCad1-F | CTTGTCGATCTGAGAAGTAAGCCCATCGAAATCAAACTATGGGCTTACTGCTCAGATCGC |
| OsMIR390-BdCad1-R | CATGGCGATCTGAGCAGTAAGCCCATAGTTTGATTTCGATGGGCTTACTTCTCAGATCGA |
| OsMIR390-BdCao-F | CTTGTCTGCATGGATTGTAAACCCATCGAAATCAAACTATGGGTTTACACTCCATGCAGC |
| OsMIR390-BdCao-R | CATGGCTGCATGGAGTGTAAACCCATAGTTTGATTTCGATGGGTTTACAATCCATGCAGA |
| OsMIR390-BdSpl11-F | CTTGTTAGCAACACTACAAGGGCACTCGAAATCAAACTAGTGCCCTTGTCGTGTTGCTAC |
| OsMIR390-BdSpl11-R | CATGGTAGCAACACGACAAGGGCACTAGTTTGATTTCGAGTGCCCTTGTAGTGTTGCTAA |
| PE Primer-F | AATGATACGGCGACCACCGAGATCTACACTCTTTCCCTACACGACGCTCTTCCGATCT |
| PE-Primer-R-N701 | CAAGCAGAAGACGGCATACGAGATTCGCCTTAGTGACTGGAGTTCAGACGTGT |
| PE-Primer-R-N702 | CAAGCAGAAGACGGCATACGAGATCTAGTACGGTGACTGGAGTTCAGACGTGT |
| PE-Primer-R-N703 | CAAGCAGAAGACGGCATACGAGATTTCTGCCTGTGACTGGAGTTCAGACGTGT |
| PE-Primer-R-N704 | CAAGCAGAAGACGGCATACGAGATGCTCAGGAGTGACTGGAGTTCAGACGTGT |
| PE-Primer-R-N705 | CAAGCAGAAGACGGCATACGAGATGGACTCCTGTGACTGGAGTTCAGACGTGT |
| PE-Primer-R-N706 | CAAGCAGAAGACGGCATACGAGATTAGGCATGGTGACTGGAGTTCAGACGTGT |
| PE-Primer-R-N707 | CAAGCAGAAGACGGCATACGAGATCTCTCTACGTGACTGGAGTTCAGACGTGT |
| PE-Primer-R-N708 | CAAGCAGAAGACGGCATACGAGATCAGAGAGGGTGACTGGAGTTCAGACGTGT |
| PE-Primer-R-N709 | CAAGCAGAAGACGGCATACGAGATGCTACGCTGTGACTGGAGTTCAGACGTGT |
| PE-Primer-R-N710 | CAAGCAGAAGACGGCATACGAGATCGAGGCTGGTGACTGGAGTTCAGACGTGT |
| Probe-amiR-173 | GTGATTTCTCTCTGCAAGCGAA |
| Probe-amiR-828 | T+GGA+ATA+CTC+ATT+TAA+GCA+AGA |
| Probe-amiR-BdBri1 | G+AGC+AAG+GCG+GAA+GAT+TGC+GA |
| Probe-amiR-BdCad1 | TGGGCTTACTTCTCAGATCGA |
| Probe-amiR-BdCao | T+GGG+TTT+ACA+ATC+CAT+GCA+GA |
| Probe-amiR-AtCh42 | AGGGATTTCCGTGACACTTAA |
| Probe-amiR-AtFt | GGCCTCTTCCTTTATAACCAA |
| Probe-amiR-BdSpl11 | GTGCCCTTGTAGTGTTGCTAA |
| Probe-amiR-AtTrich | GGCGAGCAGTATCGAATGGGA |
| Probe-U6 | AGGGGCCATGCTAATCTTCTC |
| qAtACT2-F | AAAAATGGCTGAGGCTGATGA |
| qAtACT2-R | GAAAAACAGCCCTGGGAGC |
| qAtCBP20-F | AGCTGCGCCAACGAATTATG |
| qAtCBP20-R | TCCATGGCGATTTTGTCCTC |
| qAtCH42-CS-F | CATGCACAAGTAGGGACGGTT |
| qAtCH42-CS-R | GTCACGGAAATCCTTTGGGTT |
| qAtCPC-CS-F | TCGAATGGGAAGCTGTGAAGA |
| qAtCPC-CS-R | GCGATCAACTCCCACCTGTC |
| Oligonucleotide Name | Sequence |
| qAtETC2-CS-F | GCGGTCCCAGTCTTAGGCA |
| qAtETC2-CS-R | TTCGATGCTACTCACTTCTTCAGAGT |
| qAtFT-F | TGGAACAACCTTTGGCAATG |
| qAtFT-R | CGACACGATGAATTCCTGCA |
| qAtSAND-F | CTCAAAGATTGCAGGGTACGC |
| qAtSAND-R | TCTTCAACACGCATTCCACCT |
| qAtTRY-CS-F | ACACAAAATCGCCCTCCATG |
| qAtTRY-CS-R | TCAAATCCCACCTATCACCGA |
| qAtUBQ10-F | CGCCTGCAAAGTGACTCGA |
| qAtUBQ10-R | CCAACAGCTCAACACTTTCGC |
| qBdBRI1-F | TGCACGACCGGAAAAAGATC |
| qBdBRI1-R | TGGAGAAATGCCAATCCTCG |
| qBdCAD1-CS-F | CGGAGGAGGTGCTCAAGTTC |
| qBdCAD1-CS-R | GAGCGCCTCGTTGAGGTAGT |
| qBdCAO-F | TCATGGGTGGGAGTATTCGAC |
| qBdCAO-R | TGCGCACATTGAGCATCTTT |
| qBdSAMDC-F | TGTACGAAGCTCCCCTCGG |
| qBdSAMDC-R | GCAGTTCGAGTACGCAGCAG |
| qBd-SPL11-F | AGACGTACGAGCGGACATGC |
| qBdSPL11-R | GTGTCAATGTCGTGTTCGCC |
| qBdUBC-F | CATTATCCCATGGAGGCACCT |
| qBdUBC-R | GCGGGTGACCAGGAGTCATA |
| qBdUBI4-F | GCTGTTGGAACTGCTGCTATACCT |
| qBdUBI4-R | TTGCACCAAACCAACACACACCAG |
| qBdUBI10-F | TGGACTTGCTTCTGTCTGGGTTCA |
| qBdUBI10-R | TGGTACACAGGCATAACACTGACG |

^1^ * - Phosphorothioate bond;

/5Phos/ - 5' phosphorylation
