# Supplementary material for: Highly specific gene silencing in a monocot species by artificial microRNAs derived from chimeric miRNA precursors
Source: Plant J. 2015 May 20;82(6):1061–75. doi: 10.1111/tpj.12835 (PMC4464980; doi:10.1111/tpj.12835)
Supplement: Supplementary file 20 — Table S8. Sequences and predicted targets for all amiRNAs analyzed. [file TPJ-82-1061-s020.doc]

| **Table S8.** Sequences and predicted targets for all amiRNAs analyzed. | | | | |
| --- | --- | --- | --- | --- |
| amiRNA name | amiRNA sequence (5'->3') | Predicted target(s) | Plant specie | Reference |
| amiR173-21 | UUCGCUUGCAGAGAGAAAUCA | *TAS1a, TAS1b, TAS1c, TAS2* | *Arabidopsis* *thaliana* | Cuperus *et al.*, 2010 |
| amiR472-21 | UUUUUCCUACUCCGCCCAUAC | *RFL1, RPS5, CC-NBS-LRR, NBS* | *Arabidopsis thaliana* | Cuperus *et al.*, 2010 |
| amiR828-21 | UCUUGCUUAAAUGAGUAUUCC | *MYB113, MYB82, TAS4* | *Arabidopsis thaliana* | Cuperus *et al.*, 2010 |
| amiR-AtCh42 | UUAAGUGUCACGGAAAUCCCU | *CH42* | *Arabidopsis thaliana* | Felippes and Weigel, 2009  Carbonell *et al.*, 2014 |
| amiR-AtFt | UUGGUUAUAAAGGAAGAGGCC | *FT* | *Arabidopsis thaliana* | Schwabb *et al.*, 2006  Carbonell *et al.*, 2014 |
| amiR-AtTrich | UCCCAUUCGAUACUGCUCGCC | *TRY*, *CPC*, *ETC2* | *Arabidopsis thaliana* | Schwabb *et al.*, 2006  Carbonell *et al.*, 2014 |
| amiR-BdBri1 | UCGCAAUCUUCCGCCUUGCUC | *BRI1* | *Brachypodium distachyon* | This work |
| amiR-BdCad1 | UCGAUCUGAGAAGUAAGCCCA | *CAD1* | *Brachypodium distachyon* | This work |
| amiR-BdCao | UCUGCAUGGAUUGUAAACCCA | *CAO* | *Brachypodium distachyon* | This work |
| amiR-BdSpl11 | UUAGCAACACUACAAGGGCAC | *SPL11* | *Brachypodium distachyon* | This work |
