# Supplementary material for: Highly specific gene silencing in a monocot species by artificial microRNAs derived from chimeric miRNA precursors
Source: Plant J. 2015 May 20;82(6):1061–75. doi: 10.1111/tpj.12835 (PMC4464980; doi:10.1111/tpj.12835)
Supplement: Supplementary file 21 — Table S9. High‐throughput small RNA libraries from Arabidopsis, Brachypodium or Nicotiana benthamiana plants. [file TPJ-82-1061-s021.doc]

| **Table S9.**  High-throughput small RNA libraries from Arabidopsis, Brachypodium or *Nicotiana benthamiana* plants. | | | | | | | |
| --- | --- | --- | --- | --- | --- | --- | --- |
| Sample ID | Construct | Species | Tissue | 3'PCR primer | Barcode Sequence | Adaptor-parsed reads | SRA Identifier |
| 1 | *35S:AtMIR390a-173-21* | *N. benthamiana* | Leaf | i1 | CGATGT | 25,652,072 | [SRR1771846](http://www.ncbi.nlm.nih.gov/sra/?term=SRR1771846) |
| 2 | *35S:AtMIR390a-472-21* | *N. benthamiana* | Leaf | i3 | CAGATG | 23,512,059 | [SRR1771847](http://www.ncbi.nlm.nih.gov/sra/?term=SRR1771847) |
| 3 | *35S:AtMIR390a-828-21* | *N. benthamiana* | Leaf | i5 | TTACCA | 26,746,930 | [SRR1771848](http://www.ncbi.nlm.nih.gov/sra/?term=SRR1771848) |
| 4 | *35S:AtMIR390a-OsL-173-21* | *N. benthamiana* | Leaf | i1 | CGATGT | 42,522,405 | [SRR1771851](http://www.ncbi.nlm.nih.gov/sra/?term=SRR1771851) |
| 5 | *35S:AtMIR390a-OsL-472-21* | *N. benthamiana* | Leaf | i2 | GATCAC | 47,332,026 | [SRR1771852](http://www.ncbi.nlm.nih.gov/sra/?term=SRR1771852) |
| 6 | *35S:AtMIR390a-OsL-828-21* | *N. benthamiana* | Leaf | i3 | CAGATG | 52,048,606 | [SRR1771853](http://www.ncbi.nlm.nih.gov/sra/?term=SRR1771853) |
| 7 | *35S:OsMIR390-173-21* | *B. distachyon* | Callus | i1 | CGATGT | 14,756,652 | [SRR1771445](http://www.ncbi.nlm.nih.gov/sra/?term=SRR1771445) |
| 8 | *35S:OsMIR390-472-21* | *B. distachyon* | Callus | i3 | CAGATG | 69,380,781 | [SRR1771511](http://www.ncbi.nlm.nih.gov/sra/?term=SRR1771511) |
| 9 | *35S:OsMIR390-828-21* | *B. distachyon* | Callus | i5 | TTACCA | 60,437,057 | [SRR1771523](http://www.ncbi.nlm.nih.gov/sra/?term=SRR1771523) |
| 10 | *35S:OsMIR390-AtL-173-21* | *B. distachyon* | Callus | i2 | GATCAC | 17,972,261 | [SRR1771539](http://www.ncbi.nlm.nih.gov/sra/?term=SRR1771539) |
| 11 | *35S:OsMIR390a-AtL-472-21* | *B. distachyon* | Callus | i4 | TACGTT | 25,830,535 | [SRR1771545](http://www.ncbi.nlm.nih.gov/sra/?term=SRR1771545) |
| 12 | *35S:OsMIR390a-AtL-828-21* | *B. distachyon* | Callus | i6 | ACTGTA | 25,129,002 | [SRR1771546](http://www.ncbi.nlm.nih.gov/sra/?term=SRR1771546) |
| 13 | *35S:AtMIR390a-OsL-AtCh42* | *A. thaliana* | Seedling | i10 | TGCTAG | 10,429,854 | [SRR1842772](http://www.ncbi.nlm.nih.gov/sra/?term=SRR1842772) |
| 14 | *35S:AtMIR390a-OsL-AtFt* | *A. thaliana* | Inflorescence | i11 | CTTGTA | 32,295,617 | [SRR1842774](http://www.ncbi.nlm.nih.gov/sra/?term=SRR1842774) |
| 15 | *35S:AtMIR390a-OsL-AtTrich* | *A. thaliana* | Inflorescence | i4 | TACGTT | 51,516,926 | [SRR1842775](http://www.ncbi.nlm.nih.gov/sra/?term=SRR1842775) |
| 16 | *35S:OsMIR390-BdBri1* | *B. distachyon* | Leaf | i1 | CGATGT | 19,319,670 | [SRR1771782](http://www.ncbi.nlm.nih.gov/sra/?term=SRR1771782) |
| 17 | *35S:OsMIR390-AtL-BdBri1* | *B. distachyon* | Leaf | i2 | GATCAC | 20,856,916 | [SRR1771775](http://www.ncbi.nlm.nih.gov/sra/?term=SRR1771775) |
| 18 | *35S:OsMIR390-BdCad1* | *B. distachyon* | Leaf | i5 | TTACCA | 21,308,138 | [SRR1771776](http://www.ncbi.nlm.nih.gov/sra/?term=SRR1771776) |
| 19 | *35S:OsMIR390-AtL-BdCad1* | *B. distachyon* | Leaf | I6 | ACTGTA | 22,929,175 | [SRR1771777](http://www.ncbi.nlm.nih.gov/sra/?term=SRR1771777) |
| 20 | *35S:OsMIR390-BdCao* | *B. distachyon* | Leaf | i3 | CAGATG | 21,930,111 | [SRR1771778](http://www.ncbi.nlm.nih.gov/sra/?term=SRR1771778) |
| 21 | *35S:OsMIR390-AtL-BdCao* | *B. distachyon* | Leaf | i4 | TACGTT | 22,199,088 | [SRR1771779](http://www.ncbi.nlm.nih.gov/sra/?term=SRR1771779) |
| 22 | *35S:OsMIR390-BdSpl11* | *B. distachyon* | Leaf | i7 | ATCACG | 21,231,525 | [SRR1771780](http://www.ncbi.nlm.nih.gov/sra/?term=SRR1771780) |
| 23 | *35S:OsMIR390-AtL-BdSpl11* | *B. distachyon* | Leaf | i8 | ACTTGT | 24,735,881 | [SRR1771781](http://www.ncbi.nlm.nih.gov/sra/?term=SRR1771781) |
