# Supplementary material for: Highly specific gene silencing in a monocot species by artificial microRNAs derived from chimeric miRNA precursors
Source: Plant J. 2015 May 20;82(6):1061–75. doi: 10.1111/tpj.12835 (PMC4464980; doi:10.1111/tpj.12835)
Supplement: Supplementary file 22 — Table S10. High‐throughput strand‐specific transcript RNA libraries from independent Brachypodium T0 transgenic lines. [file TPJ-82-1061-s022.doc]

| **Table S10.**  High-throughput strand-specific transcript RNA libraries from independent Brachypodium T0 transgenic lines. | | | | | |
| --- | --- | --- | --- | --- | --- |
| Sample ID | Construct | PE Primer-R Index | Index Sequence | Adaptor-parsed reads | SRA Identifier |
| 1 | *35S:GUS* | N707 | GTAGAGA | 16,779,027 | [SRR1850587](http://www.ncbi.nlm.nih.gov/sra/?term=SRR1850587) |
| 2 | *35S:GUS* | N708 | CCTCTCT | 20,182,946 | [SRR1850670](http://www.ncbi.nlm.nih.gov/sra/?term=SRR1850670) |
| 3 | *35S:GUS* | N709 | AGCGTAG | 19,472,243 | [SRR1850671](http://www.ncbi.nlm.nih.gov/sra/?term=SRR1850671) |
| 4 | *35S:GUS* | N710 | CAGCCTC | 19,128,516 | [SRR1850716](http://www.ncbi.nlm.nih.gov/sra/?term=SRR1850716) |
| 5 | *35S:OsMIR390-AtL-BdBri1* | N701 | TAAGGCG | 17,265,195 | [SRR1772223](http://www.ncbi.nlm.nih.gov/sra/?term=SRR1772223) |
| 6 | *35S:OsMIR390-AtL-BdBri1* | N702 | CGTACTA | 16,300,588 | [SRR1772224](http://www.ncbi.nlm.nih.gov/sra/?term=SRR1772224) |
| 7 | *35S:OsMIR390-AtL-BdBri1* | N703 | AGGCAGA | 15,724,668 | [SRR1772225](http://www.ncbi.nlm.nih.gov/sra/?term=SRR1772225) |
| 8 | *35S:OsMIR390-AtL-BdBri1* | N704 | TCCTGAG | 18,807,736 | [SRR1772226](http://www.ncbi.nlm.nih.gov/sra/?term=SRR1772226) |
| 9 | *35S:OsMIR390-AtL-BdCad1* | N709 | AGCGTAG | 22,853,726 | [SRR1772227](http://www.ncbi.nlm.nih.gov/sra/?term=SRR1772227) |
| 10 | *35S:OsMIR390-AtL-BdCad1* | N710 | CAGCCTC | 22,562,039 | [SRR1772228](http://www.ncbi.nlm.nih.gov/sra/?term=SRR1772228) |
| 11 | *35S:OsMIR390-AtL-BdCad1* | N701 | TAAGGCG | 16,877,134 | [SRR1772229](http://www.ncbi.nlm.nih.gov/sra/?term=SRR1772229) |
| 12 | *35S:OsMIR390-AtL-BdCad1* | N702 | CGTACTA | 17,142,684 | [SRR1772230](http://www.ncbi.nlm.nih.gov/sra/?term=SRR1772230) |
| 13 | *35S:OsMIR390-AtL-BdCao* | N705 | AGGAGTC | 18,778,386 | [SRR1772231](http://www.ncbi.nlm.nih.gov/sra/?term=SRR1772231) |
| 14 | *35S:OsMIR390-AtL-BdCao* | N706 | CATGCCT | 19,333,658 | [SRR1772232](http://www.ncbi.nlm.nih.gov/sra/?term=SRR1772232) |
| 15 | *35S:OsMIR390-AtL-BdCao* | N707 | GTAGAGA | 19,648,254 | [SRR1772233](http://www.ncbi.nlm.nih.gov/sra/?term=SRR1772233) |
| 16 | *35S:OsMIR390-AtL-BdCao* | N708 | CCTCTCT | 20,379,073 | [SRR1772234](http://www.ncbi.nlm.nih.gov/sra/?term=SRR1772234) |
| 17 | *35S:OsMIR390-AtL-BdSpl11* | N703 | AGGCAGA | 16,234,590 | [SRR1772235](http://www.ncbi.nlm.nih.gov/sra/?term=SRR1772235) |
| 18 | *35S:OsMIR390-AtL-BdSpl11* | N704 | TCCTGAG | 15,407,203 | [SRR1772236](http://www.ncbi.nlm.nih.gov/sra/?term=SRR1772236) |
| 19 | *35S:OsMIR390-AtL-BdSpl11* | N705 | AGGAGTC | 21,167,509 | [SRR1772237](http://www.ncbi.nlm.nih.gov/sra/?term=SRR1772237) |
| 20 | *35S:OsMIR390-AtL-BdSpl11* | N706 | CATGCCT | 19,068,045 | [SRR1772238](http://www.ncbi.nlm.nih.gov/sra/?term=SRR1772238) |
