# Supplementary material for: Highly specific gene silencing in a monocot species by artificial microRNAs derived from chimeric miRNA precursors
Source: Plant J. 2015 May 20;82(6):1061–75. doi: 10.1111/tpj.12835 (PMC4464980; doi:10.1111/tpj.12835)
Supplement: Supplementary file 26 — Appendix S1. Characterization of AtMIR390a‐OsL‐based amiRNAs in eudicots. [file TPJ-82-1061-s026.doc]

**Appendix S1.** Characterization of *AtMIR390a-OsL*-based amiRNAs in eudicots

**Accumulation and processing of amiRNAs produced from *AtMIR390a*- or *OsMIR390*-based precursors in *Nicotiana benthamiana***

A key feature of the *AtMIR390a-B/c*-based cloning system to produce amiRNA constructs for eudicots is that the amiRNA insert can be synthesized by annealing two relatively short 75 bases-long oligonucleotides (Carbone*ll et a*l., 2014). Because the oligonucleotides containing *OsMIR390* distal stem-loop sequences are even shorter (60 bases), we first tested if amiRNAs derived from precursors including *OsMIR390* distal stem-loop sequences could be expressed efficiently in eudicot species. This would reduce the synthesis cost of the oligonucleotides required for generating *AtMIR390a*-based amiRNA constructs, and benefit the generation of large amiRNA construct libraries for gene knockdown in eudicots such as those reported recently (Haus*er et a*l., 2013; Jover-G*il et a*l., 2014).

To test the functionality of authentic *OsMIR390* precursors to produce high levels of accurately processed small RNAs, miR390 and three different amiRNA sequences (amiR173-21, amiR472-21 and amiR828-21) (Cuper*us et a*l., 2010) were directly cloned into *pMDC32B-OsMIR390-B/c* (Figure S1, Table I) and expressed transiently in *N. benthamiana* leaves (Figure S5). The same small RNA sequences were also expressed from the chimeric *AtMIR390a-OsL* precursor including *AtMIR390a* basal stem and *OsMIR390* distal stem-loop sequences (Figure S4, Figure S8a). For comparative purposes, the same small RNA sequences were expressed from the authentic *AtMIR390a* precursor or from a chimeric precursor including *OsMIR390* basal stem and *AtMIR390a* stem-loop sequences (*OsMIR390-AtL*) (Figure S3, Figure S8a). Samples expressing the ß-glucuronidase transcript from the *35S:GUS* construct were used as negative controls.

MiR390 accumulated to similar levels when expressed from each of the different precursors (Figure S8b). In each case, amiRNAs expressed from *AtMIR390a-OsL* precursors did not accumulate to significantly different levels than did the corresponding amiRNAs produced from authentic *AtMIR390a* precursors (P> 0.11 for all pairwise *t*-test comparisons) (Figure S8b). *AtMIR390a-OsL*-derived amiRNAs accumulated predominantly to 21 nt species, suggesting that the chimeric amiRNA precursors were likely processed accurately (Figure S8b). Finally, amiRNAs produced from either authentic *OsMIR390* or chimeric *OsMIR390-AtL* precursors did not always accumulated as 21 nt species (e.g. miR828-21 and amiR472-21 from *OsMIR390* or *OsMIR390-AtL* precursors, respectively) (Figure S8b). Therefore, further analyses focused on characterizing *AtMIR390a-OsL*-based amiRNAs.

To more accurately assess processing of the amiRNA populations produced from *AtMIR390a-OsL* precursors, small RNA libraries were prepared and sequenced. For comparative purposes, small RNA libraries from samples containing *AtMIR390a*-derived amiRNAs were also analyzed. In each case, the majority of reads from either the chimeric *AtMIR390a-OsL* or authentic *AtMIR390a* precursors corresponded to correctly processed, 21 nt amiRNA (Figure S8c).

**Gene Silencing in Arabidopsis by amiRNAs derived from chimeric precursors**

To test the functionality of *AtMIR390a-OsL* based amiRNAs in repressing target transcripts, three different amiRNA constructs were introduced into *A. thaliana* Col-0 plants. For comparative purposes, the same three amiRNA sequences were also expressed from authentic *AtMIR390a* precursors as reported before (Carbone*ll et a*l., 2014). In particular, amiR-AtFt, and amiR-AtCh42 each targeted a single gene transcript [*FLOWERING LOCUS T* (*FT)* and *CHLORINA 42* (*CH42*), respectively], and amiR-AtTrich targeted three *MYB* transcripts [*TRIPTYCHON* (*TRY*), *CAPRICE* (*CPC*) and *ENHANCER OF TRIPTYCHON AND CAPRICE2* (*ETC2*)] (Figure S9). Plants including *35S:GUS* were used as negative controls. Plant phenotypes, amiRNA accumulation, mapping of amiRNA reads in *AtMIR390a-OsL* precursors and target mRNA accumulation were measured in Arabidopsis T1 transgenic lines.

Each of the 44 transformants containing *35S:AtMIR390a-OsL-Ft* was significantly delayed in flowering time compared to control plants not expressing the amiRNA (P < 0.01 two sample *t*-test, Figure S10b, Figure S11, Table S5), as previously observed in amiRNA knockdown lines (Schw*ab et a*l., 2006; Lia*ng et a*l., 2012; Carbone*ll et a*l., 2014) and *ft* mutants (Koornne*ef et a*l., 1991). Two hundred and sixty-six out of 267 transgenic lines containing *35S:AtMIR390a-OsL-Ch42* were smaller than controls and had bleached leaves and cotyledons (Figure S10c, Figure S11, Table S5), as consequence of defective chlorophyll biosynthesis and loss of Ch42 magnesium chelatase (Kon*cz et a*l., 1990; Felippes and Weigel, 2009). One hundred and seventy of these plants had a severe bleached phenotype with a lack of visible true leaves at 14 days after plating (Figure S10c, Figure S11, Table S5). Finally, 68 out of 69 lines containing *35S:AtMIR390a-OsL-Trich* had increased number of trichomes in rosette leaves; six lines had highly clustered trichomes on leaf blades like *try cpc* double mutants (Schellma*nn et a*l., 2002) or other amiR-Trich overexpressor transgenic lines (Schw*ab et a*l., 2006; Lia*ng et a*l., 2012; Carbone*ll et a*l., 2014) (Figure S10d, Table S5). The delayed flowering and trichome phenotypes were maintained in the Arabidopsis T2 progeny expressing amiR-Ft and amiR-Trich, respectively, from chimeric *AtMIR390a-OsL* precursors (Table S6). No obvious phenotypic differences were observed between plants expressing the amiRNAs from the *AtMIR390a-OsL* or *AtMIR390a* precursors in either T1 or T2 generations (Figure S10b-d, Figure S11, Tables S5 and S6). In summary, *AtMIR390-OsL*-based amiRNAs conferred a high proportion of expected and heritable target-knockdown phenotypes in transgenic plants.

The accumulation of all three amiRNAs produced from chimeric *AtMIR390-OsL* or authentic *AtMIR390a* precursors was confirmed by RNA blot analysis in T1 transgenic lines showing amiRNA-induced phenotypes (Figure S10e). In all cases, *AtMIR390-OsL*- and *AtMIR390a*-derived amiRNAs accumulated to similarly high levels and as a single species of 21 nt (Figure S10e), suggesting that *AtMIR390a-OsL*-based amiRNAs were as accurately processed as *AtMIR390a*-based amiRNAs. To more precisely assess processing and accumulation of the *AtMIR390a-OsL*-based amiRNA populations, small RNA libraries from samples containing each of the *AtMIR390a-OsL*-based constructs were prepared. In each case, the majority of reads from *AtMIR390a-OsL* precursors corresponded to correctly processed, 21 nt amiRNA while reads from the amiRNA* strands were always relatively under-represented (Figure S10g) as observed before with the same amiRNAs expressed from *AtMIR390a* precursors (Carbone*ll et a*l., 2014).

Finally, accumulation of target mRNAs in *A. thaliana* transgenic lines expressing *AtMIR390a-OsL*- or *AtMIR390a*-based amiRNAs was analyzed by quantitative real time RT-PCR assay. The expression of all target mRNAs was significantly reduced compared to control plants (P < 0.023 for all pairwise *t*-test comparisons, Figure S10f) when the specific amiRNA was expressed. No significant differences were observed in target mRNA expression between lines expressing *AtMIR390a-OsL*- or *AtMIR390a*-based amiRNAs.

Collectively, all these results indicate that amiRNAs produced from chimeric *AtMIR390a-OsL* precursors are highly expressed, accurately processed and highly effective in target gene knockdown. Therefore, the use of chimeric *AtMIR390a-OsL* precursors is an attractive alternative to express effective amiRNAs in eudicots in a cost-optimized manner.

**REFERENCES**

**Carbonell, A., Takeda, A., Fahlgren, N., Johnson, S.C., Cuperus, J.T. and Carrington, J.C.** (2014) New generation of artificial MicroRNA and synthetic trans-acting small interfering RNA vectors for efficient gene silencing in Arabidopsis. *Plant Physiol.* **165**, 15-29.

**Cuperus, J.T., Carbonell, A., Fahlgren, N., Garcia-Ruiz, H., Burke, R.T., Takeda, A., Sullivan, C.M., Gilbert, S.D., Montgomery, T.A. and Carrington, J.C.** (2010) Unique functionality of 22-nt miRNAs in triggering RDR6-dependent siRNA biogenesis from target transcripts in Arabidopsis. *Nat. Struct. Mol. Biol.* **17**, 997-1003.

**Felippes, F.F. and Weigel, D.** (2009) Triggering the formation of tasiRNAs in Arabidopsis thaliana: the role of microRNA miR173. *EMBO Rep.* **10**, 264-270.

**Hauser, F., Chen, W., Deinlein, U., Chang, K., Ossowski, S., Fitz, J., Hannon, G.J. and Schroeder, J.I.** (2013) A genomic-scale artificial microRNA library as a tool to investigate the functionally redundant gene space in Arabidopsis. *Plant Cell* **25**, 2848-2863.

**Jover-Gil, S., Paz-Ares, J., Micol, J.L. and Ponce, M.R.** (2014) Multi-gene silencing in Arabidopsis: a collection of artificial microRNAs targeting groups of paralogs encoding transcription factors. *Plant J.* **80**, 149-160.

**Koncz, C., Mayerhofer, R., Koncz-Kalman, Z., Nawrath, C., Reiss, B., Redei, G.P. and Schell, J.** (1990) Isolation of a gene encoding a novel chloroplast protein by T-DNA tagging in Arabidopsis thaliana. *EMBO J.* **9**, 1337-1346.

**Koornneef, M., Hanhart, C.J. and van der Veen, J.H.** (1991) A genetic and physiological analysis of late flowering mutants in Arabidopsis thaliana. *Mol. Gen. Genet.* **229**, 57-66.

**Liang, G., He, H., Li, Y. and Yu, D.** (2012) A new strategy for construction of artificial miRNA vectors in Arabidopsis. *Planta* **235**, 1421-1429.

**Schellmann, S., Schnittger, A., Kirik, V., Wada, T., Okada, K., Beermann, A., Thumfahrt, J., Jurgens, G. and Hulskamp, M.** (2002) TRIPTYCHON and CAPRICE mediate lateral inhibition during trichome and root hair patterning in Arabidopsis. *EMBO J.* **21**, 5036-5046.

**Schwab, R., Ossowski, S., Riester, M., Warthmann, N. and Weigel, D.** (2006) Highly specific gene silencing by artificial microRNAs in Arabidopsis. *Plant Cell* **18**, 1121-1133.
