# Supplementary material for: Highly specific gene silencing in a monocot species by artificial microRNAs derived from chimeric miRNA precursors
Source: Plant J. 2015 May 20;82(6):1061–75. doi: 10.1111/tpj.12835 (PMC4464980; doi:10.1111/tpj.12835)
Supplement: Supplementary file 28 — Appendix S3. FASTA sequences of all amiRNA‐producing MIRNA precursors analyzed. [file TPJ-82-1061-s028.doc]

**Appendix S3.** FASTA sequences of all amiRNA-producing *MIRNA* precursors analyzed.

**(a) Sequences of *OsMIR390*-based amiRNA precursors**

Sequences unique to the pri-miRNA, pre-miRNA, miRNA/amiRNA guide strand and miRNA*/amiRNA* strand sequences are highlighted in grey, white, blue and green, respectively. Bases of the pre-*OsMIR390* that had to be modified to preserve the authentic *OsMIR390* precursor structure are highlighted in red.

>**OsMIR390**

GAGATGTTTTGAGGAAGGGTATGGAACAATCCTTGAAGCTCAGGAGGGATAGCGCCTCGAAATCAAACTAGGCGCTATCTATCCTGAGCTCCATGGTTTGTTCTTACCACACGACCAATTAAATC

**>OsMIR390-AtL**

GAGATGTTTTGAGGAAGGGTATGGAACAATCCTTGAAGCTCAGGAGGGATAGCGCCATGATGATCACATTCGTTATCTATTTTTTGGCGCTATCTATCCTGAGCTCCATGGTTTGTTCTTACCACACGACCAATTAAATC

**>OsMIR390-173-21**

GAGATGTTTTGAGGAAGGGTATGGAACAATCCTTGTTCGCTTGCAGAGAGAAATCATCGAAATCAAACTATGATTTCTCTGTGTAAGCGAACATGGTTTGTTCTTACCACACGACCAATTAAATC

**>OsMIR390-AtL-173-21**

GAGATGTTTTGAGGAAGGGTATGGAACAATCCTTGTTCGCTTGCAGAGAGAAATCAATGATGATCACATTCGTTATCTATTTTTTTGATTTCTCTGTGTAAGCGAACATGGTTTGTTCTTACCACACGACCAATTAAATC

**OsMIR390-472-21**

GAGATGTTTTGAGGAAGGGTATGGAACAATCCTTGTTTTTCCTACTCCGCCCATACTCGAAATCAAACTAGTATGGGCGGCGTAGGAAAAACATGGTTTGTTCTTACCACACGACCAATTAAATC

**>OsMIR390-AtL-472-21**

GAGATGTTTTGAGGAAGGGTATGGAACAATCCTTGTTTTTCCTACTCCGCCCATACATGATGATCACATTCGTTATCTATTTTTTGTATGGGCGGCGTAGGAAAAACATGGTTTGTTCTTACCACACGACCAATTAAATC

**>OsMIR390-828-21**

GAGATGTTTTGAGGAAGGGTATGGAACAATCCTTGTCTTGCTTAAATGAGTATTCCTCGAAATCAAACTAGGAATACTCAGTTAAGCAAGACATGGTTTGTTCTTACCACACGACCAATTAAATC

**>OsMIR390-AtL-828-21**

GAGATGTTTTGAGGAAGGGTATGGAACAATCCTTGTCTTGCTTAAATGAGTATTCCATGATGATCACATTCGTTATCTATTTTTTGGAATACTCAGTTAAGCAAGACATGGTTTGTTCTTACCACACGACCAATTAAATC

**>OsMIR390-Bri1**

GAGATGTTTTGAGGAAGGGTATGGAACAATCCTTGTCGCAATCTTCCGCCTTGCTCTCGAAATCAAACTAGAGCAAGGCGTAAGATTGCGCCATGGTTTGTTCTTACCACACGACCAATTAAATC

**>OsMIR390-AtL-Bri1**

GAGATGTTTTGAGGAAGGGTATGGAACAATCCTTGTCGCAATCTTCCGCCTTGCTCATGATGATCACATTCGTTATCTATTTTTTGAGCAAGGCGTAAGATTGCGCCATGGTTTGTTCTTACCACACGACCAATTAAATC

**>OsMIR390-Cad1**

GAGATGTTTTGAGGAAGGGTATGGAACAATCCTTGTCGATCTGAGAAGTAAGCCCATCGAAATCAAACTATGGGCTTACTGCTCAGATCGCCATGGTTTGTTCTTACCACACGACCAATTAAATC

**>OsMIR390-AtL-Cad1**

GAGATGTTTTGAGGAAGGGTATGGAACAATCCTTGTCGATCTGAGAAGTAAGCCCAATGATGATCACATTCGTTATCTATTTTTTTGGGCTTACTGCTCAGATCGCCATGGTTTGTTCTTACCACACGACCAATTAAATC

**>OsMIR390-Cao**

GAGATGTTTTGAGGAAGGGTATGGAACAATCCTTGTCTGCATGGATTGTAAACCCATCGAAATCAAACTATGGGTTTACACTCCATGCAGCCATGGTTTGTTCTTACCACACGACCAATTAAATC

**>OsMIR390-AtL-Cao**

GAGATGTTTTGAGGAAGGGTATGGAACAATCCTTGTCTGCATGGATTGTAAACCCAATGATGATCACATTCGTTATCTATTTTTTTGGGTTTACACTCCATGCAGCCATGGTTTGTTCTTACCACACGACCAATTAAATC

**>OsMIR390-Spl11**

GAGATGTTTTGAGGAAGGGTATGGAACAATCCTTGTTAGCAACACTACAAGGGCACTCGAAATCAAACTAGTGCCCTTGTCGTGTTGCTACCATGGTTTGTTCTTACCACACGACCAATTAAATC

**>OsMIR390-AtL-Spl11**

GAGATGTTTTGAGGAAGGGTATGGAACAATCCTTGTTAGCAACACTACAAGGGCACATGATGATCACATTCGTTATCTATTTTTTGTGCCCTTGTCGTGTTGCTACCATGGTTTGTTCTTACCACACGACCAATTAAATC

**(b) Sequences of *AtMIR390a*-based amiRNA precursors**

Sequence unique to the pri-*AtMIR390a* sequence is highlighted in black. Bases of the pre-*AtMIR390a* that had to be modified to preserve the authentic *AtMIR390a* precursor structure are highlighted in red. Other details as in (a).

**>AtMIR390a**

TATAGGGGGGAAAAAAAGGTAGTCATCAGATATATATTTTGGTAAGAAAATATAGAAATGAATAATTTCACGTTTAACGAAGAGGAGATGACGTGTGTTCCTTCGAACCCGAGTTTTGTTCGTCTATAAATAGCACCTTCTCTTCTCCTTCTTCCTCACTTCCATCTTTTTAGCTTCACTATCTCTCTATAATCGGTTTTATCTTTCTCTAAGTCACAACCCAAAAAAACAAAGTAGAGAAGAATCTGTAAAGCTCAGGAGGGATAGCGCCATGATGATCACATTCGTTATCTATTTTTTGGCGCTATCCATCCTGAGTTTCATTGGCTCTTCTTACTACAATGAAAAAGGCCGAGGCAAAACGCCTAAAATCACTTGAGAATCAATTCTTTTTACTGTCCATTTAAGCTATCTTTTATAAACGTGTCTTATTTTCTATCTCTTTTGTTTAAACTAAGAAACTATAGTATTTTGTCTAAAACAAAACATGAAAGAACAGATTAGATCTCATCTTTAGTCTC

**>AtMIR390a-OsL**

TATAGGGGGGAAAAAAAGGTAGTCATCAGATATATATTTTGGTAAGAAAATATAGAAATGAATAATTTCACGTTTAACGAAGAGGAGATGACGTGTGTTCCTTCGAACCCGAGTTTTGTTCGTCTATAAATAGCACCTTCTCTTCTCCTTCTTCCTCACTTCCATCTTTTTAGCTTCACTATCTCTCTATAATCGGTTTTATCTTTCTCTAAGTCACAACCCAAAAAAACAAAGTAGAGAAGAATCTGTAAAGCTCAGGAGGGATAGCGCCTCGAAATCAAACTAGGCGCTATCCATCCTGAGTTTCATTGGCTCTTCTTACTACAATGAAAAAGGCCGAGGCAAAACGCCTAAAATCACTTGAGAATCAATTCTTTTTACTGTCCATTTAAGCTATCTTTTATAAACGTGTCTTATTTTCTATCTCTTTTGTTTAAACTAAGAAACTATAGTATTTTGTCTAAAACAAAACATGAAAGAACAGATTAGATCTCATCTTTAGTCTC

**>AtMIR390a-173-21**

TATAGGGGGGAAAAAAAGGTAGTCATCAGATATATATTTTGGTAAGAAAATATAGAAATGAATAATTTCACGTTTAACGAAGAGGAGATGACGTGTGTTCCTTCGAACCCGAGTTTTGTTCGTCTATAAATAGCACCTTCTCTTCTCCTTCTTCCTCACTTCCATCTTTTTAGCTTCACTATCTCTCTATAATCGGTTTTATCTTTCTCTAAGTCACAACCCAAAAAAACAAAGTAGAGAAGAATCTGTATTCGCTTGCAGAGAGAAATCAATGATGATCACATTCGTTATCTATTTTTTTGATTTCTCTGTGTAAGCGAACATTGGCTCTTCTTACTACAATGAAAAAGGCCGAGGCAAAACGCCTAAAATCACTTGAGAATCAATTCTTTTTACTGTCCATTTAAGCTATCTTTTATAAACGTGTCTTATTTTCTATCTCTTTTGTTTAAACTAAGAAACTATAGTATTTTGTCTAAAACAAAACATGAAAGAACAGATTAGATCTCATCTTTAGTCTC

**>AtMIR390a-OsL-173-21**

TATAGGGGGGAAAAAAAGGTAGTCATCAGATATATATTTTGGTAAGAAAATATAGAAATGAATAATTTCACGTTTAACGAAGAGGAGATGACGTGTGTTCCTTCGAACCCGAGTTTTGTTCGTCTATAAATAGCACCTTCTCTTCTCCTTCTTCCTCACTTCCATCTTTTTAGCTTCACTATCTCTCTATAATCGGTTTTATCTTTCTCTAAGTCACAACCCAAAAAAACAAAGTAGAGAAGAATCTGTATTCGCTTGCAGAGAGAAATCATCGAAATCAAACTATGATTTCTCTGTGTAAGCGAACATTGGCTCTTCTTACTACAATGAAAAAGGCCGAGGCAAAACGCCTAAAATCACTTGAGAATCAATTCTTTTTACTGTCCATTTAAGCTATCTTTTATAAACGTGTCTTATTTTCTATCTCTTTTGTTTAAACTAAGAAACTATAGTATTTTGTCTAAAACAAAACATGAAAGAACAGATTAGATCTCATCTTTAGTCTC

**AtMIR390a-472-21**

TATAGGGGGGAAAAAAAGGTAGTCATCAGATATATATTTTGGTAAGAAAATATAGAAATGAATAATTTCACGTTTAACGAAGAGGAGATGACGTGTGTTCCTTCGAACCCGAGTTTTGTTCGTCTATAAATAGCACCTTCTCTTCTCCTTCTTCCTCACTTCCATCTTTTTAGCTTCACTATCTCTCTATAATCGGTTTTATCTTTCTCTAAGTCACAACCCAAAAAAACAAAGTAGAGAAGAATCTGTATTTTTCCTACTCCGCCCATACATGATGATCACATTCGTTATCTATTTTTTGTATGGGCGGCGTAGGAAAAACATTGGCTCTTCTTACTACAATGAAAAAGGCCGAGGCAAAACGCCTAAAATCACTTGAGAATCAATTCTTTTTACTGTCCATTTAAGCTATCTTTTATAAACGTGTCTTATTTTCTATCTCTTTTGTTTAAACTAAGAAACTATAGTATTTTGTCTAAAACAAAACATGAAAGAACAGATTAGATCTCATCTTTAGTCTC

**AtMIR390a-OsL-472-21**

TATAGGGGGGAAAAAAAGGTAGTCATCAGATATATATTTTGGTAAGAAAATATAGAAATGAATAATTTCACGTTTAACGAAGAGGAGATGACGTGTGTTCCTTCGAACCCGAGTTTTGTTCGTCTATAAATAGCACCTTCTCTTCTCCTTCTTCCTCACTTCCATCTTTTTAGCTTCACTATCTCTCTATAATCGGTTTTATCTTTCTCTAAGTCACAACCCAAAAAAACAAAGTAGAGAAGAATCTGTATTTTTCCTACTCCGCCCATACTCGAAATCAAACTAGTATGGGCGGCGTAGGAAAAACATTGGCTCTTCTTACTACAATGAAAAAGGCCGAGGCAAAACGCCTAAAATCACTTGAGAATCAATTCTTTTTACTGTCCATTTAAGCTATCTTTTATAAACGTGTCTTATTTTCTATCTCTTTTGTTTAAACTAAGAAACTATAGTATTTTGTCTAAAACAAAACATGAAAGAACAGATTAGATCTCATCTTTAGTCTC

**>AtMIR390a-828-21**

TATAGGGGGGAAAAAAAGGTAGTCATCAGATATATATTTTGGTAAGAAAATATAGAAATGAATAATTTCACGTTTAACGAAGAGGAGATGACGTGTGTTCCTTCGAACCCGAGTTTTGTTCGTCTATAAATAGCACCTTCTCTTCTCCTTCTTCCTCACTTCCATCTTTTTAGCTTCACTATCTCTCTATAATCGGTTTTATCTTTCTCTAAGTCACAACCCAAAAAAACAAAGTAGAGAAGAATCTGTATCTTGCTTAAATGAGTATTCCATGATGATCACATTCGTTATCTATTTTTTGGAATACTCAGTTAAGCAAGACATTGGCTCTTCTTACTACAATGAAAAAGGCCGAGGCAAAACGCCTAAAATCACTTGAGAATCAATTCTTTTTACTGTCCATTTAAGCTATCTTTTATAAACGTGTCTTATTTTCTATCTCTTTTGTTTAAACTAAGAAACTATAGTATTTTGTCTAAAACAAAACATGAAAGAACAGATTAGATCTCATCTTTAGTCTC

**>AtMIR390a-OsL-828-21**

TATAGGGGGGAAAAAAAGGTAGTCATCAGATATATATTTTGGTAAGAAAATATAGAAATGAATAATTTCACGTTTAACGAAGAGGAGATGACGTGTGTTCCTTCGAACCCGAGTTTTGTTCGTCTATAAATAGCACCTTCTCTTCTCCTTCTTCCTCACTTCCATCTTTTTAGCTTCACTATCTCTCTATAATCGGTTTTATCTTTCTCTAAGTCACAACCCAAAAAAACAAAGTAGAGAAGAATCTGTATCTTGCTTAAATGAGTATTCCTCGAAATCAAACTAGGAATACTCAGTTAAGCAAGACATTGGCTCTTCTTACTACAATGAAAAAGGCCGAGGCAAAACGCCTAAAATCACTTGAGAATCAATTCTTTTTACTGTCCATTTAAGCTATCTTTTATAAACGTGTCTTATTTTCTATCTCTTTTGTTTAAACTAAGAAACTATAGTATTTTGTCTAAAACAAAACATGAAAGAACAGATTAGATCTCATCTTTAGTCTC

**>AtMIR390a-Ch42**

TATAGGGGGGAAAAAAAGGTAGTCATCAGATATATATTTTGGTAAGAAAATATAGAAATGAATAATTTCACGTTTAACGAAGAGGAGATGACGTGTGTTCCTTCGAACCCGAGTTTTGTTCGTCTATAAATAGCACCTTCTCTTCTCCTTCTTCCTCACTTCCATCTTTTTAGCTTCACTATCTCTCTATAATCGGTTTTATCTTTCTCTAAGTCACAACCCAAAAAAACAAAGTAGAGAAGAATCTGTATTAAGTGTCACGGAAATCCCTATGATGATCACATTCGTTATCTATTTTTTAGGGATTTCCTTGACACTTAACATTGGCTCTTCTTACTACAATGAAAAAGGCCGAGGCAAAACGCCTAAAATCACTTGAGAATCAATTCTTTTTACTGTCCATTTAAGCTATCTTTTATAAACGTGTCTTATTTTCTATCTCTTTTGTTTAAACTAAGAAACTATAGTATTTTGTCTAAAACAAAACATGAAAGAACAGATTAGATCTCATCTTTAGTCTC

**>AtMIR390a-OsL-Ch42**

TATAGGGGGGAAAAAAAGGTAGTCATCAGATATATATTTTGGTAAGAAAATATAGAAATGAATAATTTCACGTTTAACGAAGAGGAGATGACGTGTGTTCCTTCGAACCCGAGTTTTGTTCGTCTATAAATAGCACCTTCTCTTCTCCTTCTTCCTCACTTCCATCTTTTTAGCTTCACTATCTCTCTATAATCGGTTTTATCTTTCTCTAAGTCACAACCCAAAAAAACAAAGTAGAGAAGAATCTGTATTAAGTGTCACGGAAATCCCTTCGAAATCAAACTAAGGGATTTCCTTGACACTTAACATTGGCTCTTCTTACTACAATGAAAAAGGCCGAGGCAAAACGCCTAAAATCACTTGAGAATCAATTCTTTTTACTGTCCATTTAAGCTATCTTTTATAAACGTGTCTTATTTTCTATCTCTTTTGTTTAAACTAAGAAACTATAGTATTTTGTCTAAAACAAAACATGAAAGAACAGATTAGATCTCATCTTTAGTCTC

**>AtMIR390a-Ft**

TATAGGGGGGAAAAAAAGGTAGTCATCAGATATATATTTTGGTAAGAAAATATAGAAATGAATAATTTCACGTTTAACGAAGAGGAGATGACGTGTGTTCCTTCGAACCCGAGTTTTGTTCGTCTATAAATAGCACCTTCTCTTCTCCTTCTTCCTCACTTCCATCTTTTTAGCTTCACTATCTCTCTATAATCGGTTTTATCTTTCTCTAAGTCACAACCCAAAAAAACAAAGTAGAGAAGAATCTGTATTGGTTATAAAGGAAGAGGCCATGATGATCACATTCGTTATCTATTTTTTGGCCTCTTCCGTTATAACCAACATTGGCTCTTCTTACTACAATGAAAAAGGCCGAGGCAAAACGCCTAAAATCACTTGAGAATCAATTCTTTTTACTGTCCATTTAAGCTATCTTTTATAAACGTGTCTTATTTTCTATCTCTTTTGTTTAAACTAAGAAACTATAGTATTTTGTCTAAAACAAAACATGAAAGAACAGATTAGATCTCATCTTTAGTCTC

**>AtMIR390a-OsL-Ft**

TATAGGGGGGAAAAAAAGGTAGTCATCAGATATATATTTTGGTAAGAAAATATAGAAATGAATAATTTCACGTTTAACGAAGAGGAGATGACGTGTGTTCCTTCGAACCCGAGTTTTGTTCGTCTATAAATAGCACCTTCTCTTCTCCTTCTTCCTCACTTCCATCTTTTTAGCTTCACTATCTCTCTATAATCGGTTTTATCTTTCTCTAAGTCACAACCCAAAAAAACAAAGTAGAGAAGAATCTGTATTGGTTATAAAGGAAGAGGCCTCGAAATCAAACTAGGCCTCTTCCGTTATAACCAACATTGGCTCTTCTTACTACAATGAAAAAGGCCGAGGCAAAACGCCTAAAATCACTTGAGAATCAATTCTTTTTACTGTCCATTTAAGCTATCTTTTATAAACGTGTCTTATTTTCTATCTCTTTTGTTTAAACTAAGAAACTATAGTATTTTGTCTAAAACAAAACATGAAAGAACAGATTAGATCTCATCTTTAGTCTC

**>AtMIR390a-Trich**

TATAGGGGGGAAAAAAAGGTAGTCATCAGATATATATTTTGGTAAGAAAATATAGAAATGAATAATTTCACGTTTAACGAAGAGGAGATGACGTGTGTTCCTTCGAACCCGAGTTTTGTTCGTCTATAAATAGCACCTTCTCTTCTCCTTCTTCCTCACTTCCATCTTTTTAGCTTCACTATCTCTCTATAATCGGTTTTATCTTTCTCTAAGTCACAACCCAAAAAAACAAAGTAGAGAAGAATCTGTATCCCATTCGATACTGCTCGCCATGATGATCACATTCGTTATCTATTTTTTGGCGAGCAGTCTCGAATGGGACATTGGCTCTTCTTACTACAATGAAAAAGGCCGAGGCAAAACGCCTAAAATCACTTGAGAATCAATTCTTTTTACTGTCCATTTAAGCTATCTTTTATAAACGTGTCTTATTTTCTATCTCTTTTGTTTAAACTAAGAAACTATAGTATTTTGTCTAAAACAAAACATGAAAGAACAGATTAGATCTCATCTTTAGTCTC

**>AtMIR390a-OsL-Trich**

TATAGGGGGGAAAAAAAGGTAGTCATCAGATATATATTTTGGTAAGAAAATATAGAAATGAATAATTTCACGTTTAACGAAGAGGAGATGACGTGTGTTCCTTCGAACCCGAGTTTTGTTCGTCTATAAATAGCACCTTCTCTTCTCCTTCTTCCTCACTTCCATCTTTTTAGCTTCACTATCTCTCTATAATCGGTTTTATCTTTCTCTAAGTCACAACCCAAAAAAACAAAGTAGAGAAGAATCTGTATCCCATTCGATACTGCTCGCCTCGAAATCAAACTAGGCGAGCAGTCTCGAATGGGACATTGGCTCTTCTTACTACAATGAAAAAGGCCGAGGCAAAACGCCTAAAATCACTTGAGAATCAATTCTTTTTACTGTCCATTTAAGCTATCTTTTATAAACGTGTCTTATTTTCTATCTCTTTTGTTTAAACTAAGAAACTATAGTATTTTGTCTAAAACAAAACATGAAAGAACAGATTAGATCTCATCTTTAGTCTC
