# Supplementary material for: Highly specific gene silencing in a monocot species by artificial microRNAs derived from chimeric miRNA precursors
Source: Plant J. 2015 May 20;82(6):1061–75. doi: 10.1111/tpj.12835 (PMC4464980; doi:10.1111/tpj.12835)
Supplement: Supplementary file 29 — Appendix S4. Protocol to clone amiRNAs in BsaI/ccdB‐based (‘B/c’) vectors including the OsMIR390 precursor. [file TPJ-82-1061-s029.doc]

**Appendix S4**

Protocol to clone amiRNAs in *Bsa*I/*ccd*B-based (‘B/c’) vectors including the *OsMIR390* precursor.

*Notes:*

*-Available OsMIR390 B/c vectors are listed in Table I at the end of this protocol.*

*-*OsMIR390-B/c*-based vectors must be propagated in a* ccd*B resistant* E. coli *strain such as DB3.1.*

-Alternatively, Bsa*I digestion of the B/c vector and subsequent ligation of the amiRNA oligonucleotide insert can be done in separate reactions*

3.1. Oligonucleotide annealing

-Dilute sense oligonucleotide and antisense oligonucleotide in sterile H2O to a final concentration of 100 μM.

-Prepare Oligo Annealing Buffer:

60 mM Tris-HCl (pH 7.5)

500 mM NaCl

60 mM MgCl_2_

10 mM DTT

***Note:*** *Prepare 1 ml aliquots of Oligo Annealing Buffer and store at -20^0^C.*

-Assemble the annealing reaction in a PCR tube as described below:

Forward oligonucleotide (100 μM) 2 μL

Reverse oligonucleotide (100 μM) 2 μL

Oligo Annealing Buffer 46 μL

Total volume 50 μL

The final concentration of each oligonucleotide is 4 μM.

-Use a thermocycler to heat the annealing reaction 5 min at 94°C and then cool down (0.05ºC/sec) to 20°C.

-Dilute the annealed oligonucleotides just prior to assembling the digestion-ligation reaction as described below:

Annealed oligonucleotides 3 μL

dH_2_O 37 μL

Total volume 40 μL

The final concentration of each oligonucleotide is 0.15 μM.

***Note****: Do not store the diluted oligonucleotides.*

3.2. Digestion-ligation reaction

- Assemble the digestion-ligation reaction as described below:

B/c vector (x ug/uL) Y μL (50 ng)

Diluted annealed oligonucleotides 1 μL

10x T4 DNA ligase buffer 1 μL

T4 DNA ligase (400 U/μL) 1 μL

*Bsa*I (10U/ μL, NEB) 1 μL

dH_2_O to 10 μL

Total volume 10 μL

Prepare a negative control reaction lacking *Bsa*I.

-Mix the reactions by pipetting. Incubate the reactions for 5 minutes at 37°C.

3.3. *E.coli* transformation and analysis of transformants

-Transform 1-5 ul of the digestion-ligation reaction into an *E. coli* strain that doesn't have *ccd*B resistance (e.g. DH10B, TOP10, …) to do counter-selection.

-Pick two colonies/construct, grow LB-Kan (100 mg/ml) cultures and purify plasmids.

-Sequence with appropriate primers: M13-F (CCCAGTCACGACGTTGTAAAACGACGG) and M13-R (CAGAGCTGCCAGGAAACAGCTATGACC) for *pENTR*-based vectors; attB1 (ACAAGTTTGTACAAAAAAGCAGGCT) and attB2 (ACCACTTTGTACAAGAAAGCTGGGT) primers for *pMDC32B*-, *pMDC123SB*- or *pH7WG2B*-based vectors).

| **Table 1:** *OsMIR390-BsaI/ccdB* (‘B/c’) vectors for direct cloning of amiRNAs. | | | | | | | | | | | | | | |
| --- | --- | --- | --- | --- | --- | --- | --- | --- | --- | --- | --- | --- | --- | --- |
| Vector | | Bacterial antibiotic resistance | | Plant antibiotic resistance | | GATEWAY use | Backbone | | Promoter | Terminator | | Plant species tested | | Addgene ID |
| *pENTR-OsMIR390-B/c* | Kanamycin | | - | | Donor | | *pENTR* | - | | - | - | | 61468 | |
| *pMDC123SB-OsMIR390-B/c* | Kanamycin | | BASTA | | - | | *pMDC123* | *CaMV* 2x35S | | *nos* | *Nicotiana benthamiana* | | 61466 | |
| *pMDC32B-OsMIR390-B/c* | Kanamycin  Hygromycin | | Hygromycin | | - | | *pMDC32* | *CaMV* 2x35S | | *nos* | *Nicotiana benthamiana*  *Brachypodium distachyon* | | 61467 | |
| *pH7WG2B-OsMIR390-B/c* | Spectinomycin | | Hygromycin | | - | | *pH7WG2* | *Os Ubiquitin* | | *CaMV* | *Brachypodium distachyon* | | 61465 | |
