# Supplementary material for: Highly specific gene silencing in a monocot species by artificial microRNAs derived from chimeric miRNA precursors
Source: Plant J. 2015 May 20;82(6):1061–75. doi: 10.1111/tpj.12835 (PMC4464980; doi:10.1111/tpj.12835)
Supplement: Supplementary file 30 [file TPJ-82-1061-s030.doc]

**LEGENDS TO SUPPORTING INFORMATION**

**Figure S1.** *OsMIR390-B/c* vectors for direct cloning of amiRNAs.

(a) Diagram of an *OsMIR390-B/c* Gateway-compatible entry vector (*pENTR-OsMIR390-B/c)*.

(b) Diagrams of *OsMIR390-B/c*-based binary vectors for expression of amiRNAs in monocot species (*pMDC32B-OsMIR390-B/c*, *pMDC123SB-OsMIR390-B/c* and *pH7WG2B-OsMIR390-B/c*). RB: right border; 35S: *Cauliflower mosaic virus* promoter; OsUbi: *Oryza sativa* ubiquitin 2 promoter; *Bsa*I: *Bsa*I recognition site, *ccd*B: gene encoding the *ccd*B toxin; LB: left border; attL1 and attL2: gateway recombination sites. *Kan^R^*: kanamycin resistance gene; *Hyg^R^*: hygromycin resistance gene; *Basta^R^*: glufosinate resistance gene; *Spec^R^*: spectinomycin resistance gene. Undesired *Bsa*I sites removed from the plasmid are crossed out.

**Figure S2.** Generation of constructs to express amiRNAs from authentic *OsMIR390* precursors.

(a) Design of the two overlapping oligonucleotides required for amiRNA cloning into *OsMIR390*-based vectors. Sequences covered by the forward and reverse oligonucleotides are represented with solid and dotted lines, respectively. Nucleotides of *OsMIR390* precursor, amiRNA guide strand, and amiRNA* strand are in grey, blue, and green respectively. Other *OsMIR390* nucleotides that may be modified for preserving authentic *OsMIR390* precursor secondary structure are in red. Rules for assigning identity to positions 1 and 9 of amiRNA* are indicated.

(b) Diagram of the steps for amiRNA cloning in *OsMIR390* precursors. The amiRNA insert obtained after annealing the two overlapping oligonucleotides has 5’CTTG and 5’CATG overhangs and is directly inserted in a directional manner into an *OsMIR390-B/c* vector previously linearized with *Bsa*I. Nucleotides of the *Bsa*I sites and those arbitrarily chosen and used as spacers between the *Bsa*I recognition sites and the *OsMIR390* sequence are in purple and light brown, respectively. Other details are as described in A. C, flow chart of the steps from amiRNA construct generation to plant transformation.

**Figure S3.** Generation of constructs to express amiRNAs from chimeric *OsMIR390-AtL* precursors.

(a) Design of the two overlapping oligonucleotides containing *OsMIR390aa* and *AtMIR390a* basal stem and distal stem loop sequences, respectively. Sequences covered by the forward and reverse oligonucleotides are represented with solid and dotted lines, respectively. Nucleotides of *AtMIR390a* and *OsMIR390* precursors are in black and grey, respectively. Nucleotides of the amiRNA guide strand, and amiRNA* strand are in blue, and green respectively. Other *OsMIR390* nucleotides that may be modified for preserving authentic *OsMIR390* precursor secondary structure are in red. Rules for assigning identity to positions 1 and 9 of amiRNA* are indicated.

(b) Diagram of the steps for generating constructs for expressing amiRNAs from chimeric *OsMIR390-AtL* precursors. The amiRNA insert obtained after annealing the two overlapping oligonucleotides has 5’CTTG and 5’CATG overhangs and is directly inserted in a directional manner into an *OsMIR390-B/c* vector previously linearized with *Bsa*I. Nucleotides of the *Bsa*I sites and those arbitrarily chosen and used as spacers between the *Bsa*I recognition sites and the *OsMIR390* sequence are in purple and light brown, respectively. Other details are as described in (a).

(c) Flow chart of the steps from amiRNA construct generation to plant transformation.

**Figure S4.** Generation of constructs to express amiRNAs from chimeric *AtMIR390a-OsL* precursors.

(a) Design of the two overlapping oligonucleotides containing *AtMIR390a* and *OsMIR390* basal stem and distal stem loop sequences, respectively. Sequences covered by the forward and reverse oligonucleotides are represented with solid and dotted lines, respectively. Nucleotides of *AtMIR390a* and *OsMIR390* precursors are in black and grey, respectively. Nucleotides of the amiRNA guide strand, and amiRNA* strand are in blue, and green respectively. Other *AtMIR390a* nucleotides that may be modified for preserving authentic *AtMIR390a* precursor secondary structure are in red. Rules for assigning identity to position 9 of amiRNA* are indicated.

(b) Diagram of the steps for generating constructs for expressing amiRNAs from chimeric *AtMIR390a-OsL* precursors. The amiRNA insert obtained after annealing the two overlapping oligonucleotides has 5’TGTA and 5’AATG overhangs and is directly inserted in a directional manner into an *AtMIR390a-B/c* vector previously linearized with *Bsa*I. Nucleotides of the *Bsa*I sites and those arbitrarily chosen and used as spacers between the *Bsa*I recognition sites and the *AtMIR390a* sequence are in purple and light brown, respectively. Other details are as described in (a).

(c) Flow chart of the steps from miRNA construct generation to plant transformation.

**Figure S5**. Base-pairing of amiRNAs and Brachypodium target mRNAs. amiRNA and mRNA target nucleotides are in blue and brown, respectively.

**Figure S6**. Plant height and seed length analyses in *Brachypodium distachyon* T0 transgenic plants expressing amiR-BdBri1 from authentic *OsMIR390* or chimeric *OsMIR390-AtL* precursors.

**Figure S7**. Quantification of amiR-BdCao-induced phenotype in *Brachypodium distachyon* *35S:OsMIR390-AtL-Cao*, *35S:OsMIR390-Cao* and *35S:GUS* T0 transgenic lines.

(a) Quantification of chlorophyll a, chlorophyll b, chlorophyll a+b, chlorophyll a/b, and carotenoid content.

(b) Absorbance spectra from 400 to 750 nm of leaves from Brachypodium transgenic lines. Arrows indicate absorbance wavelengths of chlorophyll a (Chl a), chlorophyll b (Chl b), and carotenoids.

**Figure S8**. Comparative analysis of the accumulation and processing of several amiRNAs produced from *AtMIR390a*, *AtMIR390a-OsL*, *OsMIR390* and *OsMIR390-AtL* based precursors in *Nicotiana benthamiana* leaves.

(a) Diagrams of *AtMIR390a*, *AtMIR390a-OsL*, *OsMIR390* and *OsMIR390a-AtL* precursors. Nucleotides corresponding to the miRNA guide strand are in blue, and nucleotides of the miRNA* strand are in green. Other nucleotides from the *AtMIR390a* and *OsMIR390* precursors are in black and grey, respectively. Shapes of the *AtMIR390a* and *OsMIR390* precursors are in black and grey, respectively.

(b) Accumulation of miR390 (left) and of several 21-nucleotide amiRNAs (right) expressed from the *AtMIR390a*, *AtMIR390a-OsL*, *OsMIR390* or *OsMIR390-AtL* precursors in *N. benthamiana* leaves. Mean (n=3) relative amiRNA levels + s.d. when expressed from the *AtMIR390a* (dark blue, amiRNA level =1.0). Only one blot from three biological replicates is shown. U6 RNA blot is shown as loading control.

(c) Processing analysis of *AtMIR390a* and *AtMIR390-OsL* amiRNA precursors. Pie charts show the percentage of reads corresponding to accurately processed 21-nt mature amiRNAs (blue sectors) or to other small RNAs (pink sectors).

**Figure S9.** Base-pairing of amiRNAs and Arabidopsis target transcripts. amiRNA and mRNA target nucleotides are in blue and brown, respectively.

**Figure S10.** Functionality in Arabidopsis T1 transgenic plants of amiRNAs derived from *AtMIR390a*-based chimeric precursors containing *Oryza sativa* distal stem-loop sequences (*AtMIR390a-OsL*).

(a) *AtMIR390a*- and *AtMIR390a-OsL*-based precursors containing Ft-, Ch42- and Trich-amiRNAs. Nucleotides corresponding to the miRNA guide and miRNA* strands are in blue and green, respectively; nucleotides from the *AtMIR390a* or *OsMIR390* precursors are in black or grey, respectively, except those that were modified to preserve authentic *AtMIR390a* or *OsMIR390* precursor secondary structures that are in red.

(b-d) Representative images of plants expressing amiRNAs from *AtMIR390a-OsL* or *AtMIR390a-OsL* precursors.

(b) Adult control plant (*35S:GUS*) or plants expressing *35S:AtMIR390a-Ft-OsL* or *35S:AtMIR390a-Ft* plant with a delayed flowering phenotype.

(c) Ten days-old seedlings expressing *35S:AtMIR390a-OsL-Ch42* or *35S:AtMIR390a-Ch42* and showing bleaching phenotypes.

(d) Fifteen days-old control seedling (*35S:GUS*), or seedling expressing *35S:AtMIR390a-OsL-Trich* or *35S:AtMIR390a-Trich* with increased number of trichomes.

(e) Accumulation of amiRNAs in transgenic plants. One blot from three biological replicates is shown. Each biological replicate is a pool of at least 8 independent plants. U6 RNA blot is shown as a loading control.

(f) Mean relative level +/- s.e. of *A. thaliana* *FT*, *CH42*, *TRY*, *CPC* and *ETC2* mRNAs after normalization to *ACT2*, *CPB20*, *SAND* and *UBQ10*, as determined by quantitative real-time RT-PCR (*35S:GUS* = 1.0 in all comparisons).

(g) Mapping of amiRNA reads from *AtMIR390a-OsL* precursors expressed in transgenic plants. Analysis of amiRNA and amiRNA* reads in plants expressing amiR-AtFt (left), amiR-AtCh42 (center) and amiR-AtTrich (right), respectively. amiRNA guide and amiRNA* strands are highlighted in blue and green, respectively. Nucleotides from *AtMIR390a* or *OsMIR390* precursors are in black and grey, respectively, except those that were modified to preserve the corresponding authentic precursor secondary structure that are in red. Proportion of small RNA reads are plotted as stacked bar graphs. Small RNAs are color-coded by size.

**Figure S11.** AmiRNA-induced phenotype quantification in Arabidopsis transgenic plants expressing amiR-AtFt (left) and amiR-AtCh42 (right) from *AtMIR390a* or chimeric *AtMIR390a-OsL* precursors.

**Figure S12**. Target accumulation determined by RNA-Seq analysis in transgenic Brachypodium plants including *35S:OsMIR390-AtL*-based or *35S:GUS* constructs.

**Table S1.** MiRbase locus identifiers of *Orzya sativa* conserved *MIRNA* precursors.

**Table S2.** MiRbase locus identifiers of plant *MIR390* precursors.

**Table S3**: AmiRNA phenotypic penetrance in Brachypodium T0 transgenic plants.

**Table S4**: AmiRNA phenotypic penetrance in Brachypodium T1 transgenic plants.

**Table S5**: AmiRNA phenotypic penetrance in Arabidopsis T1 transgenic plants.

**Table S6**: AmiRNA phenotypic penetrance in Arabidopsis T2 transgenic plants.

**Table S7.** DNA, LNA and RNA oligonucleotides.

**Table S8.** Sequences and predicted targets for all amiRNAs analyzed.

**Table S9.** High-throughput small RNA libraries from Arabidopsis, Brachypodium or *Nicotiana benthamiana* plants.

**Table S10.** High-throughput strand-specific transcript RNA libraries from independent Brachypodium T0 transgenic lines.

**Data S1A.** Differential gene expression analysis between *35S:GUS* and *35S:OsMIR390-AtL-Bri1* Brachypodium samples.

**Data S1B.** Differential gene expression analysis between *35S:GUS* and *35S:OsMIR390-AtL-Cad1* Brachypodium samples.

**Data S1C.** Differential gene expression analysis between *35S:GUS* and *35S:OsMIR390-AtL-Cao* Brachypodium samples.

**Data S1D.** Differential gene expression analysis between *35S:GUS* and *35S:OsMIR390-AtL-Spl11* Brachypodium samples.

**Data S2.** Gene counts in RNA-Seq libraries from *35S:GUS, 35S:0sMIR390-AtL-Bri1, 35S:OsMIR390-AtL-Cad1, 35S:0sMIR390-AtL-Cao* and *35S:OsMIR390-AtL-Spl11* Brachypodium transgenic lines.

**Data S3A.** amiR-BdBri1 predicted off-targets differentially underexpressed in *35S:OsMIR390-AtL-Bri1* transgenic Brachypodium plants.

**Data S3B.** amiR-BdCad1 predicted off-targets differentially underexpressed in *35S:OsMIR390-AtL-Cad1* transgenic Brachypodium plants.

**Data S3C.** amiR-BdCao predicted off-targets differentially underexpressed in *35S:OsMIR390-AtL-Cao* transgenic Brachypodium plants.

**Data S3D.** amiR-BdSpl11 predicted off-targets differentially underexpressed in *35S:OsMIR390-AtL-Spl11* transgenic Brachypodium plants.

**Appendix S1.** Characterization of *AtMIR390a-OsL*-based amiRNAs in eudicots.

**Appendix S2**. DNA sequence of B/c vectors used for direct cloning of amiRNAs in zero-background vectors containing the *OsMIR390* sequence.

**Appendix S3.** FASTA sequences of all amiRNA-producing *MIRNA* precursors analyzed. (A) *OsMIR390* precursors. Sequences unique to the pri-miRNA, pre-miRNA, miRNA/amiRNA guide strand and miRNA*/amiRNA* strand sequences are highlighted in grey, white, blue and green, respectively. Bases of the pre-*OsMIR390* that had to be modified to preserve the authentic *OsMIR390* precursor structure are highlighted in red. (B) *AtMIR390a* precursors. Sequence unique to the pri-*AtMIR390a* sequence is highlighted in black. Bases of the pre-*AtMIR390a* that had to be modified to preserve the authentic *AtMIR390a* precursor structure are highlighted in red. Other details as in (A).

**Appendix S4.** Protocol to clone amiRNAs in *Bsa*I/*ccd*B-based (‘B/c’) vectors including the *OsMIR390* precursor.
